# Valorization of Traditional Italian Walnut (*Juglans regia* L.) Production: Genetic, Nutritional and Sensory Characterization of Locally Grown Varieties in the Trentino Region

**DOI:** 10.3390/plants11151986

**Published:** 2022-07-30

**Authors:** Erica A. Di Pierro, Pietro Franceschi, Isabella Endrizzi, Brian Farneti, Lara Poles, Domenico Masuero, Iuliia Khomenko, Francesco Trenti, Annarita Marrano, Urska Vrhovsek, Flavia Gasperi, Franco Biasioli, Graziano Guella, Luca Bianco, Michela Troggio

**Affiliations:** 1 Fruit Crop Genetics and Breeding Unit, Research and Innovation Center, Fondazione Edmund Mach, 38098 Trento, Italy; michela.troggio@fmach.it; 2 Computational Biology Unit, Research and Innovation Center, Fondazione Edmund Mach, 38098 Trento, Italy; pietro.franceschi@fmach.it (P.F.); luca.bianco@fmach.it (L.B.); 3 Sensory Quality Unit, Research and Innovation Center, Fondazione Edmund Mach, 38098 Trento, Italy; isabella.endrizzi@fmach.it (I.E.); iuliia.khomenko@fmach.it (I.K.); flavia.gasperi@fmach.it (F.G.); franco.biasioli@fmach.it (F.B.); 4 Berries Genetics and Breeding Unit, Research and Innovation Center, Fondazione Edmund Mach, 38098 Trento, Italy; brian.farneti@fmach.it; 5 Food and Environment (Di3A), Department of Agriculture, University of Catania, 95131 Catania, Italy; lara.poles@gmail.com; 6 Metabolomics Unit, Research and Innovation Center, Fondazione Edmund Mach, 38098 Trento, Italy; domenico.masuero@fmach.it (D.M.); urska.vrhovsek@fmach.it (U.V.); 7 Bioorganic Chemistry Lab, Department of Physics, University of Trento, 38123 Trento, Italy; f.trenti@unitn.it (F.T.); graziano.guella@unitn.it (G.G.); 8 Department of Plant Sciences, University of California, Davis, CA 95616, USA; annarita.marrano@umb.edu

**Keywords:** Persian walnut, food quality, varietal identification, secondary metabolites, essential fatty acids, volatile organic compounds, consumer acceptability

## Abstract

*Juglans regia* (L.) is cultivated worldwide for its nutrient-rich nuts. In Italy, despite the growing demand, walnut cultivation has gone through a strong decline in recent decades, which led to Italy being among the top five net importing countries. To promote the development of local high-quality Italian walnut production, we devised a multidisciplinary project to highlight the distinctive traits of three varieties grown in the mountainous region Trentino (northeast of Italy): the heirloom ‘Bleggiana’, a second local accession called *local Franquette* and the French cultivar ‘Lara’, recently introduced in the local production to increase yield. The genetic characterization confirmed the uniqueness of ‘Bleggiana’ and revealed *local Franquette* as a newly described autochthonous variety, thus named ‘Blegette’. The metabolic profiles highlighted a valuable nutritional composition of the local varieties, richer in polyphenols and with a lower ω-6/ω-3 ratio than the commercial ‘Lara’. ‘Blegette’ obtained the highest preference scores from consumers for both the visual aspect and tasting; however, the volatile organic compound profiles did not discriminate among the characterized cultivars. The described local varieties represent an interesting reservoir of walnut genetic diversity and quality properties, which deserve future investigation on agronomically useful traits (e.g., local adaptation and water usage) for a high-quality and sustainable production.

## 1. Introduction

Common walnut (*Juglans regia* L.), also known as English walnut or Persian walnut, is probably the most economically important member of the genus *Juglans*. Grown worldwide in temperate areas, *J. regia* varieties are cultivated primarily for nut production as varietal clones [1]. Walnuts are an important source of antioxidants and anti-inflammatory compounds, being rich in polyphenols (phenolic acids, flavonoids, tannins, etc.) and other phytochemicals [2,3]. Phenolic compounds are considered as health-promoting components in plant-derived foods, and many studies have investigated their high antioxidative and antiradical potentials [4,5]. The hydrolysable tannins, composed of gallotannins and ellagitannins, represent the major phenolic class determined in walnuts [6,7,8]. Several studies have investigated the anti-inflammatory properties of ellagitannins and their potential protective role against a wide range of pathologies and their complications [2,9,10]. For example, the ellagic acid can be converted by the gut bacteria to urolithin A and other derivatives, which can be considered key molecules in the modulation of hormone- and hormone-receptor-dependent diseases, such as, breast and prostate cancers [2].

The lipid profile of walnuts is rich in mono- (oleic, 10–20%) and poly-unsaturated fatty acids (α-linolenic, 10–18%, and linoleic, 55–70%) with well-balanced ω-6/ω-3 ratio and lower levels of saturated fatty acids [11,12,13]. The important properties of walnut nutrients are associated with many health benefits. It is well recognized that dietary ω-3 α-linolenic acid may have a number of cardioprotective actions [14,15]; moreover, epidemiological evidence has shown that the changing ratio of ω-6 vs. ω-3 fatty acids in ‘Western diets’, with an increased uptake of ω-6 fatty acids, may heavily affect relevant pathologies, such as coronary artery disease, cancer and autoimmunity [16,17].

As a consequence, walnuts are among the most widely consumed tree nuts in the world and are a highly recommended food in the human daily diet [18].

In Italy, walnuts are produced across the entire peninsula, from the Alps to Sicily, from sea level to 1000–1200 m elevation, and the species is well adapted to the range of environmental conditions characteristic of the country [19,20]. However, walnut cultivation in Italy experienced a strong decline, from 33,000 ha in the 1960s to 3441 ha in 2011, with a very slight increase to about 5000 ha in the last few years (FAO, Rome, Italy, 2022. FAOSTAT data). As a consequence, Italy has been among the top five net importing countries of in-shell walnuts since the mid-1970s (FAO, 2022. FAOSTAT data). Nevertheless, the climate and environmental conditions of the Italian peninsula are well suited for walnut cultivation [21], and still, some local unique Italian varieties have survived.

On-farm, traditionally cultivated varieties or landraces represent a significant reservoir of crop genetic diversity [22] adapted to marginal or specific agricultural ecosystems in terms of biotic and abiotic stress tolerance [23,24,25,26]. In addition, traditional crop varieties are valuable in many aspects, such as dietary, nutritional, cultural, and for promoting a sustainable regional economic development [27,28,29,30,31].

This is the case of the ‘Bleggiana’ cultivar, grown on the plateau of Bleggio, at 628 m above sea level (a.s.l.), in the mountainous region of Trentino in the northeast of Italy [32]. This area is historically suited for this cultivation, as demonstrated by the native variety ‘Bleggiana’ developed in this area decades ago by local farmers and still cultivated and propagated by grafting. The ‘Bleggiana’ walnut, registered as Slow Food Presidium [26], is characterized by small fruits, a thin shell, a light-colored kernel and an excellent flavor [32]. In addition to the walnut ‘Bleggiana’, another local accession is grown in this area, which is similar to the French variety ‘Franquette’ in terms of fruit size, kernel color and the apical-bearing habit, and thus commonly called by local farmers *local Franquette*.

Despite a growing interest in crop landraces and zero km food, in the global context, the walnut market is moved by a quantitative approach based on intensive and specialized cultivations, where China, USA and Iran are the leading producing countries (FAO, 2019. FAOSTAT data). Therefore, as for other agronomic species [23,30,33,34], the low competitiveness with products from the foreign market could lead to the abandonment, and consequent extinction, of traditional walnut varieties. Moreover, the monocultural industrial farming models are not immune from crop failures and significant reductions in yield, particularly in the current context of global climate change. Widespread cultivations of just a few uniform domesticated varieties are more vulnerable to biotic (pathogen and pests) and abiotic stresses, also resulting in the need for pesticides and other inputs that harm non-target biota [35,36]. Agro-biodiversity promotion could be at the basis of sustainable food security [37], through the identification and protection of new crops and varieties with important nutrition content and adapted to the local agro-climatic zones [36,38]. Therefore, projects for the valorization of traditional products are relevant to improve local agri-food supply chains and to build a unique value for consumers. Indeed, consumer awareness and the constantly growing demand for beneficial and high-quality foods, such as walnuts and nuts in general, are stimulating farmers to invest more in the nut sector. Accordingly, the current context is favorable to promote walnut cultivation on the Italian territory, encouraging the development of a local, high-quality market.

The current study was conducted within the ‘NoBle project’ for the valorization of traditional walnut varieties in the Italian Alpine region of Trentino, with the aim to provide a comprehensive characterization of the unique and typical walnut accessions of this territory.

Therefore, a multidisciplinary approach was applied to:

(i) genetically characterize the ‘Bleggiana’ and *local Franquette* accessions. Single sequence repeat (SSR) and single nucleotide polymorphism (SNP) markers were used to genotype the local accessions from Trentino and two popular Italian walnut landraces [19,39]. These latter ones are *Feltrina*, present only in the mountainous area of Feltre in the Veneto region, and *Sorrento*, spread across the Sorrento peninsula in the Campania region [40,41]. In order to understand how they relate to one another, both locally and globally, international commercial cultivars were also genotyped;
(ii) highlight distinctive nutritional and volatile profiles that uniquely define the walnut ‘Bleggiana’ and *local Franquette* in comparison to ‘Lara’, a commercial cultivar recently introduced in the same cultivation area to enhance yield;
(iii) explore consumers’ attitudes and preferences through a consumer acceptance test.

To our knowledge, the present work provides the first example of an extensive characterization of traditional nut fruit varieties for their value enhancement and scientifically sound promotion.

## 2. Results and Discussion

### 2.1. Italian Walnut Genetic Diversity and Cluster Analysis

Among the 33 *J. regia* accessions analyzed (Table S1), 11 are traditionally cultivated in the Italian peninsula, including genotypes from the northeast of Italy, such as the ‘Bleggiana’, the *local Franquette* and the *Feltrina* ecotype, and from the south, where the *Sorrento* landrace is mainly present. The remaining accessions represent the most popular American and French commercial cultivars.

Genetic diversity estimates for each SSR locus are given in Table 1. All loci analyzed here are polymorphic, and the most informative locus is WGA005 with ten alleles and the highest values of expected heterozygosity (H_exp_) and Shannon index (H). The 11 SSR markers distinguished 31 unique multi-locus profiles that included the established commercial cultivars, the ‘Bleggiana’ genotypes, the *local Franquette* and three *Sorrento* ecotypes, which showed to have identical SSR profiles: SoE_mast, SoE_m2, SoE_ac2 (Table S2).

Discrimination of the ‘Bleggiana’ profile within the current set of samples is possible with just one SSR marker, WGA005, due to the presence of the private allele (267 bp), while the most informative markers to discriminate the *local Franquette* profiles are WGA005, WGA069 and/or WGA118. Overall, in this study, five SSR markers are enough to discriminate between unique genotypes, as revealed by the genotype accumulation curve (Figure S1), and the five most informative SSR loci are WGA005, WGA321, WGA276, WGA069 and WGA032.

These results are consistent with those reported by Foroni et al. [19,40], which evaluated the genetic diversity of the *Sorrento* landrace in relation to the ‘Bleggiana’ and some commercial cultivars using 12 SSR markers. They also reported one private allele for the cultivar ‘Bleggiana’ at locus WGA005 (265 bp), which is confirmed in the present study.

**Figure 1:**
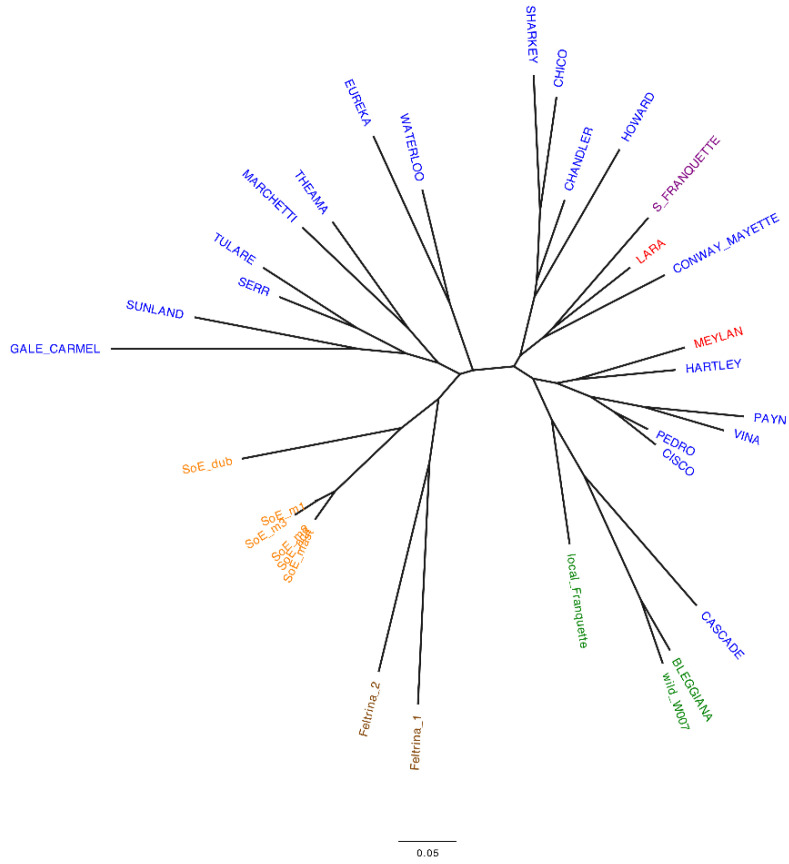
Unrooted neighbor-joining tree produced from Bruvo’s distance matrix estimated from SSR data. Labels are colored according to the geographical origin: green = northeastern Italy—Trentino; brown = northeastern Italy—Veneto; orange = south Italy—Campania; red = France; blue = USA; violet = France/USA.

**Figure 2:**
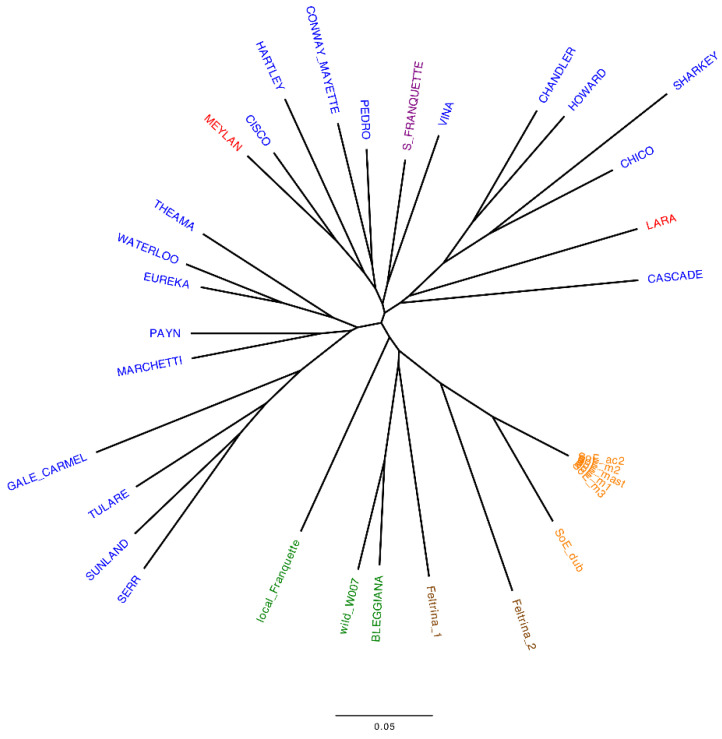
Unrooted neighbor-joining tree produced from Prevosti’s distance matrix estimated from SNP data. Labels are colored according to the geographical origin: green = northeastern Italy—Trentino; brown = northeastern Italy—Veneto; orange = south Italy—Campania; red = France; blue = USA; violet = France/USA.

The uniqueness of the Italian accessions was assessed by both SSR (Table S2) and SNP markers (File S1). The neighbor-joining (NJ) trees produced from the cluster analysis are presented in Figure 1 and Figure 2 for the SSR and SNP data, respectively.

The dendrograms efficiently summarize the genetic marker data, showing the relationships among the studied accessions [42]. For both marker types, the clustering reflects the geographic origin of the accessions, as also described in previous studies [41,43,44].

However, some differences between the two sets of markers are present. The NJ tree produced using SNP markers (Figure 2) clearly shows a unique group of Italian accessions only. Within it, all walnuts from Sorrento cluster together with the walnut genotype from Veneto, Feltrina_2, while the cultivar ‘Bleggiana’ forms a second subgroup, including the wild accession from the Bleggio area and the other walnut genotype from Veneto, Feltrina_1. The accession *local Franquette*, although included in the Italian cluster, emerges as the more distant one, whereas using SSR markers (Figure 1), both Feltrina_1 and Feltrina_2 are tightly related and come out closer to the *Sorrento* genotypes than to the other accessions from the northeast of Italy. ‘Bleggiana’, *local Franquette* and the wild accession from Bleggio form an independent group, which includes the American cultivar ‘Cascade’.

Additionally, five *Sorrento* ecotypes (SoE_mast, SoE_ac2, SoE_m1, SoE_m2, SoE_m3) are identified by SNP data as genetic duplicates, identical at 99.98%, on average. This result is in contrast to the outcome described by SSR data, where SoE_m1 and SoE_m3, although being related to SoE_mast, SoE_ac2 and SoE_m2, have different genetic profiles.

Our results confirmed the already known relationship among the economically important American and French varieties [41,42,45,46] and highlighted the more complex characterization of the *Sorrento* and *Feltrina* landraces. The mixture of the genotypes reflects the old attitude of local growers to propagate cultivars by seed as well as by grafting [40,42]. This results in the heterogeneous phenotypic variability for important commercial traits, such as fruit size and yield, of these accessions. Therefore, it is appropriate to consider *Sorrento* and *Feltrina* as landraces rather than varieties. Concerning the locally grown varieties in the Trentino region, the results obtained with both marker types in all screened individuals (Table S2, File S1) confirm both the ‘Bleggiana’ and *local Franquette* as authentic cultivars with unique genetic profiles maintained by local growers across generations through grafting. Furthermore, no relation is shown between the original ‘Franquette’ and *local Franquette*, which is closer to the accessions of the Bleggio area. This suggests a local origin of this latter variety, although it likely derives from an old controlled cross with an American cultivar. In the current study, we describe for the first time the accession *local Franquette*, which is revealed to be a new autochthonous variety of the Trentino region. Therefore, to highlight its typical genetic profile and the link to the area of cultivation, the new variety was named ‘Blegette’, and from now on in the text, we will refer to *local Franquette* as ‘Blegette’.

### 2.2. Characterization of Local Walnuts by Metabolic Profiles

In order to characterize the nutritional properties of the traditional cultivars of the Trentino region, ‘Bleggiana’ and ‘Blegette’, their polyphenol and lipid profiles were compared to a commercial variety recently introduced in the local cultivation, ‘Lara’. Five trees for each of the three walnut varieties were sampled, for a total of 15 samples, for three consecutive years.

#### 2.2.1. Phenolic Compounds and Ellagitannins

**Figure 3:**
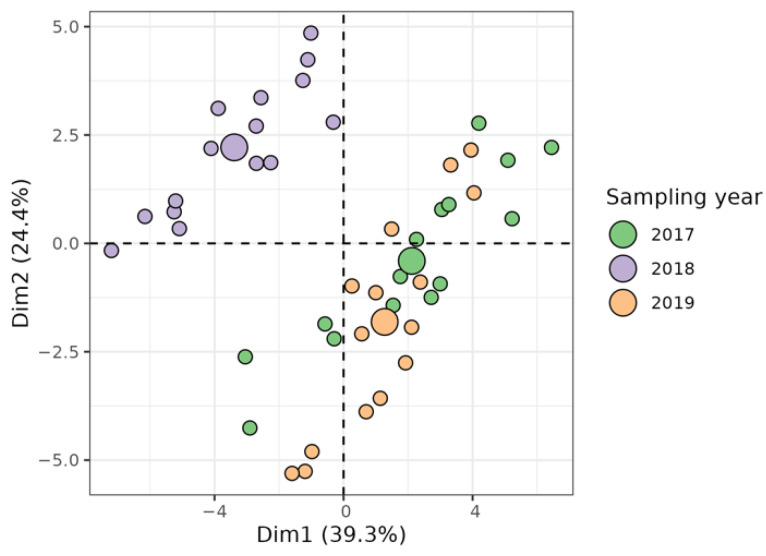
Principal component analysis (PCA) of three walnut cultivars (‘Bleggiana’, ‘Blegette’ and ‘Lara’) of three harvesting seasons, based on polyphenol composition, obtained from LC–MS/MS. The score plot shows the first two PCs, which account for 63.7% of the total variance, and a clear separation between the samples harvested in 2018 and the ones collected in 2019 and 2017. Large circles show the centroid of each sampling year cluster.

The results of the quantitative characterization of phenolics and ellagitannins in the whole raw kernel are summarized in Table S3. Principal component analysis (PCA) was used to characterize the most influential factors that affected the walnut metabolome. The results are shown as a score plot in Figure 3, where the projection of the first principal component (PC1) vs. the second principal component (PC2) accounts for 63.7% of the total variance. The plot shows a clear separation between the samples harvested in 2018 and the ones collected in 2019 and 2017. This prominent role of the year is actually expected due to the influence of the environment on the concentration of secondary metabolites [7,47].

**Figure 4:**
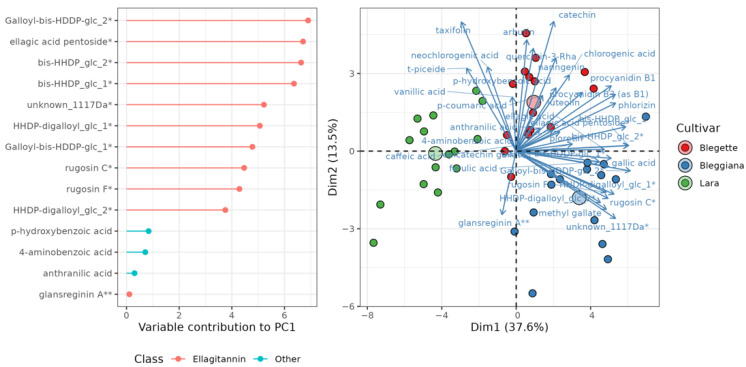
Principal component analysis (PCA) of three walnut cultivars (‘Bleggiana’, ‘Blegette’ and ‘Lara’) based on 35 polyphenolic constituents after removal of the year effect. The score plot (on the right) shows the first two PCs, which account for 50% of the total variance, and a clear separation between the three cultivars; large circles show the centroid of each cultivar cluster. The contribution of the 14 compounds that mainly discriminate among cultivars along PC1 is shown in the variable importance plot on the left; these compounds largely represent the class of ellagitannins (highlighted in red). * quantified as ellagic acid; ** quantified as gallic acid.

In order to evaluate the effect of the cultivar, the harvesting year effect was removed (see Section 3.2.4), and PCA was performed again to investigate the structure of the data matrix. The results are summarized in Figure 4, where the separation between the three varieties is clear in the PC1 vs. PC2 plane, accounting for 50% of the overall variance. PC1 is particularly important in separating the three varieties. The length and the direction of the variable arrows show that ‘Bleggiana’ and ‘Blegette’ were consistently richer in almost all phenolic metabolites detected. This suggests a longer shelf life of these varieties, where the skin covering the kernel acts as a phenolic-rich barrier to protect the fatty acids from oxidation [48,49,50].

As far as the separation between the three cultivars is concerned, ellagitannins seem to hold a prominent role, as shown by the variable importance plot (Figure 4). In this respect, ‘Bleggiana’ is expected to have the largest concentration of this class of compounds, followed by ‘Blegette’ and ‘Lara’. This type of compounds can be hydrolyzed to break down into ellagic acid molecules that can therefore be used for direct quantification of ellagitannins [7,51] (Figure 4). 

Ellagitannins are among the major phenolic classes determined in walnuts [6,7,8] and have been reported to act as strong astringents [52]. This is consistent with our results, where the low polyphenols concentration observed for the commercial cultivar ‘Lara’ supports the characteristic low level of bitterness described by Germain et al. [53] and also reported in the sensory study of Colaric et al. [54] for this cultivar.

**Figure 5:**
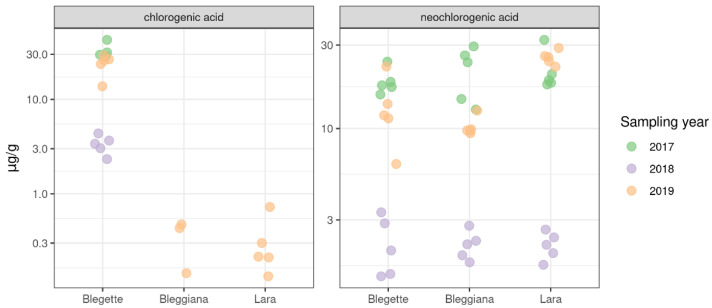
Dot plot of chlorogenic and neochlorogenic acid concentration in the samples of three walnut cultivars (‘Bleggiana’, ‘Blegette’ and ‘Lara’) collected in three different harvesting seasons (2017, 2018, 2019). Quantification was obtained by ultraperformance liquid chromatography coupled with tandem mass spectrometry (UPLC-MS/MS) in the whole raw kernel. Chlorogenic acid seems to be absent in cultivars ‘Bleggiana’ and ‘Blegette’, being undetected in 2017 and 2018, and just very close to the detection limit in 2019. Conversely, the concentration levels of neochlorogenic acid are similar in all the three cultivars and consistent across the harvesting seasons.

PCA analysis is extremely useful to give an overall view on the effect of the variety on the overall metabolic profile. In addition to this analysis, the profiles of the individual metabolites can be used to spot specific varietal differences, which could be potentially interesting for the understanding of the associations between genetic background and metabolism. In the case of walnuts, chlorogenic acid represented an intriguing example. The concentration profile of this compound across the three years of sampling is shown in Figure 5. The plot clearly shows that in all three years considered in the present investigation, chlorogenic acid was two orders of magnitude more concentrated in ‘Blegette’ than in the other two cultivars. Despite the genetic relatedness and the adaptation to a common environment of ‘Blegette’ and ‘Bleggiana’, our results suggest a different metabolic pattern in the biosynthesis or accumulation of chlorogenic acid for the cultivar ‘Blegette’.

In walnuts, the presence of chlorogenic acid (5-CQA) and its isomer neochlorogenic acids (3-CQA) was previously described in several studies on the phenolic profiles of walnut [6,7,49,55,56]. The reported content of 5-CQA ranged from undetected (below the detection limit) in the Chinese cultivar ‘Wen185’ [55] to 0.78 μg/g in the American cultivar ‘Payne’ [56], therefore consistent with those detected for ‘Bleggiana’ and ‘Lara’ in the current study (Figure 5; Table S3). When quantified [7,55], the 3-CQA was at higher levels than 5-CQA, as observed in our study for ‘Bleggiana’ and ‘Lara’(Figure 5; Table S3). In the present study, 3-CQA was detected in all three cultivars at similar concentration levels; however ‘Blegette’ was the only one to show comparable amounts of both 5-CQA and 3-CQA. The processes involved in 5-CQA isomerization are poorly understood [57]. Enzymatic trans-esterification of 5-CQA might be responsible for the production of 3-CQA [58]; however, nothing is known about the enzymes and genes involved in walnut.

#### 2.2.2. Lipid Profile

**Figure 6:**
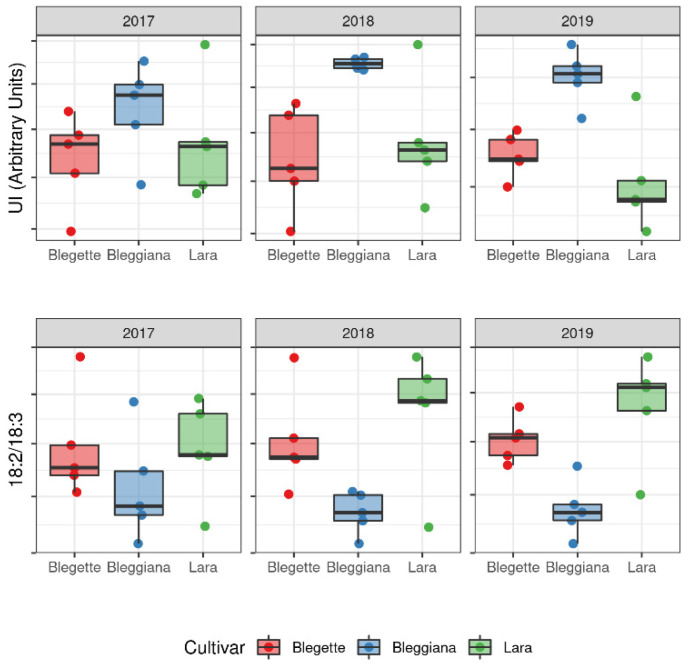
Boxplot for the unsaturation index (UI) value and ω-6/ω-3 ratio determined in three harvesting seasons (2017, 2018 and 2019) for three walnut cultivars (‘Bleggiana’, ‘Blegette’ and ‘Lara’).

In nuts, the lipid profile is important to characterize the nutritional potential of a cultivar. Most nuts contain mostly monounsaturated fatty acids (MUFA), while walnuts are highly enriched in ω-6 and ω-3 polyunsaturated fatty acids (PUFA) [59,60,61]. Among the different lipidomic parameters, the unsaturation index (UI) and the relative contribution of ω-3 and ω-6 fatty acids are particularly relevant, and the trend of these parameters across the samples included in our study is shown in Figure 6.

In terms of UI, the ‘Bleggiana’ samples show a consistently higher level across the three years of sampling, with the other two cultivars having comparable values. As far as the ω-6/ω-3 ratio is concerned, our investigation indicates that, despite a large variability, ‘Lara’ consistently showed a larger ratio of ω-6/ω-3 (about 5.6) fatty acids with respect to the ω-6/ω-3 ratio in ‘Blegette’ and ‘Bleggiana’ (about 4.1). This difference mainly derives from the significantly higher relative amount of the major ω-6 linoleic acid (18:2) in ‘Lara’ (64 ± 1%) with respect to ‘Blegette’ and ‘Bleggiana’ (55 ±1%), rather than a significant decrease in the minor linolenic acid (18:3). Nevertheless, ‘Bleggiana’ has the highest relative amount (13 ± 1%) of this ω-3 fatty acid, thus also explaining its highest UI among the cultivars investigated. Overall, the lipidomic data obtained for the cultivar ‘Bleggiana’ show the lowest relative amount (8.7 ± 0.5%) of saturated fatty acids (SFA) and the highest relative amount (13 ± 1%) of ω-3 lipids, suggesting the best qualities from a dietary and health point of view for this cultivar.

### 2.3. Walnut Volatilome Phenotyping by Proton-Transfer-Reaction Time-of-Flight Mass Spectrometry (PTR-ToF-MS)

**Figure 7:**
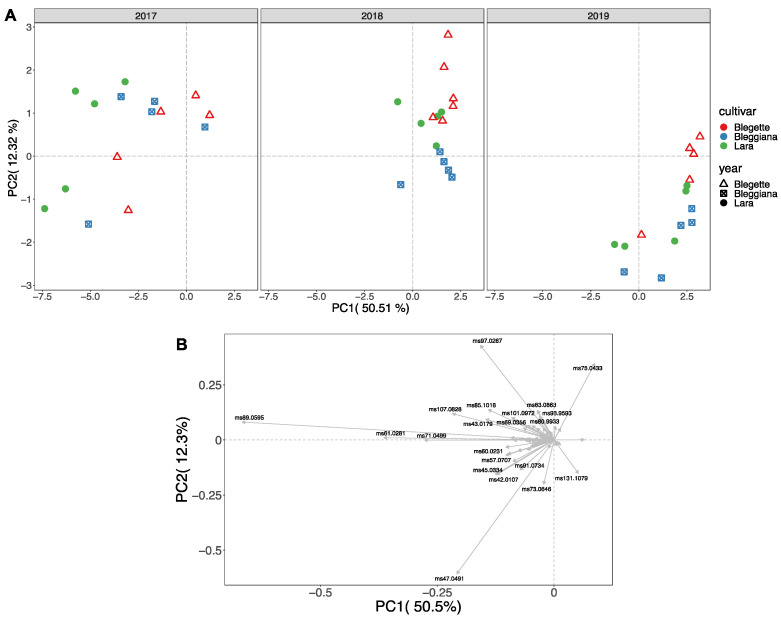
Principal component analysis (PCA) (**A**) and loading plot (**B**) of the VOC profiles, assessed by PTR-ToF-MS, of the walnut accessions ‘Bleggiana’, ‘Blegette’ and ‘Lara’ of three harvesting seasons (2017, 2018 and 2019). Each point of the PCA plot is the average of three technical replicates.

**Figure 8:**
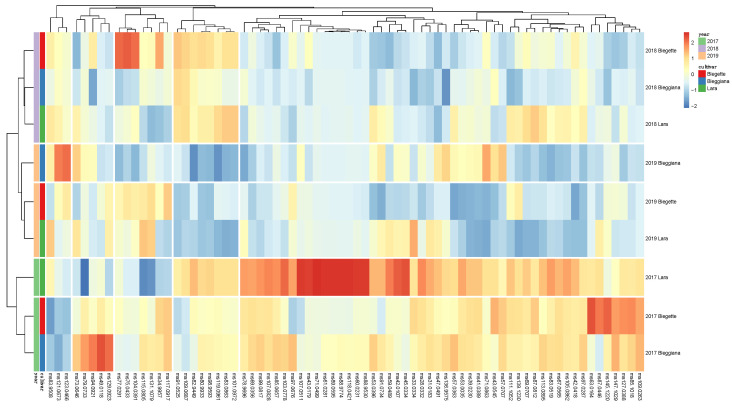
Heat map and two-dimensional hierarchical dendrograms of the VOC profile, assessed by PTR-ToF-MS, of the walnut accessions ‘Bleggiana’, ‘Blegette’ and ‘Lara’, of three harvesting seasons (2017, 2018 and 2019). Cluster analysis was performed using Ward’s method on centered and scaled data. Walnut samples are grouped and clustered by rows, while VOC compounds are organized by columns.

In our study, PTR-ToF-MS analyses allowed the detailed characterization of the metabolomics of volatile organic compounds (VOCs), known as ‘’volatilomics’’, of three walnut cultivars (‘Bleggiana’, ‘Blegette’ and ‘Lara’), revealing significant differences in VOC composition between the three years of cultivation (Figure 7 and Figure 8).

Walnut VOC profile was assessed on raw kernels in triplicate by PTR-ToF-MS analysis, as described in Di Guardo et al. [62]. VOC mass peaks from the raw PTR-ToF-MS spectra were reduced from 318 to 73, applying noise and correlation coefficient thresholds (Table S4). Tentative identification (t.i.) of each mass peak, detected by PTR-ToF-MS, was based on an in-house library of pure standards and on the literature information [63]. To our knowledge, this is the first work regarding PTR-MS application on walnut kernels. The application of direct injection mass spectrometry techniques (DIMS), such as PTR-ToF-MS, has recently been demonstrated as a powerful phenotyping tool for destructive and non-destructive assessment of nut and dried fruit volatilome in both genetic and quality related studies [62,64]. The headspace analyses carried out with DIMS techniques allow for the assessment of VOCs that are often omitted from the usual gas chromatographic measurements despite their importance for the characterization of fruit quality and freshness, such as methanol (m/z 33.033), ethanol (m/z 47.049) or acetaldehyde (m/z 45.033). There are several studies concerning the volatile and oxidative stability of walnut oil based on gas chromatographic assessments [65,66,67]. However, only few studies were carried out on the changes of volatile components and oxidation stability of walnut kernels [63,68,69].

Among these 73 mass peaks, 57 were significantly different among production years based on the two-way analysis of variance (ANOVA), while only 26 differed among accessions (Table S4). Based on recently published papers, walnut VOC profile is mostly formed by compounds originated from the decomposition of linoleic acid, such as 1-pentanol, 1-hexenol, pentanal and hexanal [63]. In particular, Crowe et al. [70] showed that the levels of hexanal increased as walnut sensory quality deteriorated due to increasing oxidation. Other linoleic acid oxidation products, such as 2-pentylfuran, 1-octen-3-ol and (E)-2-heptenal, were demonstrated to be key VOCs to distinguish walnuts of different origin. 1-penten-3-ol is another characteristic VOC formed from α-linolenic acid, the third most prevalent fatty acid of walnut [69]. In the current study, no statistically significant differences were found in the comparison of the three walnut accessions based on the concentration of compounds resulting from the oxidation of linoleic and linolenic acid, such as t.i. 1-pentanol (*m/z* 71.0863); t.i. 1-hexenol (*m/z* 83.0519); t.i. pentanal (*m/z* 87.0812); t.i. hexanal (*m/z* 101.0972); t.i. (E) -2- heptenal (*m/z* 113.0895); t.i. 1-penten-3-ol (*m/z* 69.0707); t.i. 3-methyl-1-pentanol (*m/z* 85.1018); t.i. 2-methyl-2-propanol (*m/z* 57.0707) (Figure 8 and Figure S2; Table S4). The analyses carried out on the samples of three harvest years revealed significant differences for walnuts sampled in the first year of experimentation (2017), in particular with higher values and greater biological variability between replicates.

However, PCA analysis showed that none of these compounds was among the first ten most discriminant VOCs. The variability expressed by the first two components (about 63%) is mostly described by the following masses: *m/z* 47.049 (ethanol), *m/z* 89.059 (t.i. ethyl acetate), *m/z* 61.028 (acetic acid), *m/z* 71.049 (t.i. butenal), *m/z* 97.0287 (t.i. 4-cyclopentene-1,3-dione), *m/z* 107.082 (t.i. xylene), *m/z* 75.043 (t.i. methyl acetate), *m/z* 131.107 (t.i. 2-methylbutyl acetate), *m/z* 45.033 (t.i. acetaldehyde). As for the compounds related to lipid oxidation, also these latter compounds exhibited the effect of the production year on the VOC profile variability, which was more significant than the genetic differences between accessions. These results disclosed how a comprehensive analysis of the aromatic profile of walnut is not only useful for a detailed metabolic/qualitative characterization of different accessions but, above all, also for obtaining a quality control of the product based on agronomic practices, climatic conditions and conservation practices [71]. These factors may, in fact, be more crucial for the final composition of the walnut aroma than the plant genetic variability.

Even if the aromatic composition is often not considered as a predominant component of walnut quality, sensory studies [72,73] have shown how the sensory attributes of ‘floral flavor’ and ‘flavor intensity’ play a significant role in defining the consumer perceived quality. In particular, both quality traits support the identification and discrimination of accessions based mainly on the origin differences [72].

### 2.4. Consumer Study

As part of the current study, a total of 188 subjects aged between 18 and 75 years (54% females, 46 years of mean age) took part in the consumer test that was conducted in 2018. From the analysis of socio-demographic data, it emerged that participants have a high level of education: 51% declared a bachelor’s/master’s degree or higher. Furthermore, 72% of participants reported they live with their families, and 51% did not have children. With regard to lifestyle and behavioral habits, it emerged that the involved consumers adopt, on average, a healthy lifestyle: 55% claimed they have never smoked, and 79% practiced sports up to twice a week.

Considering 30 g of nuts as equivalent to one serving, 48% of participants stated they eat three or more servings of shell fruit per week; in particular, 28% eat three or more servings of walnuts. For both shell fruit and walnuts, the purchase takes place more frequently in the supermarket, occasionally from the producer or the greengrocer and almost never at the market.

Shell fruit is a very popular product, with M > 6, where M represents the average scores for stated liking on a 9-point hedonic scale. Walnuts, hazelnuts, almonds and pistachios are the most appreciated families, while pine nuts and peanuts are less popular. Participants reported they buy mostly (70%) unshelled and (80%) non-pre-packaged fruit. ‘Origin’ and ‘Organic production’ are the factors that participants indicated as the most important in the purchase process, while ‘variety’ is indicated as the least important factor. On average, participants consider eating walnuts more appropriate for breakfast and as a snack between meals, and less suitable as a meal replacement or after meals. Fresh consumption of walnuts without any preparation is preferred to the consumption in recipes (e.g., cakes, sweets) or as an ingredient for sauces or granola. Moreover, knowing that a kilo of walnuts costs EUR 8.00 on average, consumers would be willing to pay EUR 1.5 more for an ‘organic’ or ‘zero food miles’ product and EUR 1 more for products of ‘first choice’ or of higher nutritional properties. These results are compatible with walnut consumption in Italy, which settles at 620 g per capita per year, with an annual average increase of 4% in the last 10 years [74], likely due to a greater awareness of the health benefits of walnuts among consumers.

In Table 2, the acceptability mean values on tasting and the mean rank for visual preference with the relative information about statistical homogenous groups are reported. On tasting, all four varieties are more than acceptable, with average values between 6.38 and 6.95 with a comparable variability. Walnut variety factor has a significant effect on consumer liking based on ANOVA one-way model (*F*(3, 748) = 4.926, *p* = 0.002). ‘Lara’ and ’Blegette’ obtained, on average, the highest acceptability score, statistically different from that obtained by ‘Chandler’, the least appreciated one. ‘Bleggiana’ is not statistically different for acceptability from other varieties.

In previous studies, ‘Lara’ was reported among the least bitter and astringent walnut cultivars [53,54]; conversely, the higher level of polyphenols observed for ‘Blegette’ suggests a stronger astringency [49,52]. Our results indicate a certain degree of variability in consumer perception of the taste properties of walnut kernels, as previously reported by Colarič and colleagues [54]. Similarly to our study, the evaluation was performed by untrained consumers of different ages. With regard to kernel taste in particular, they observed a major difference among consumers in the perception of astringency and bitterness.

Even for the visual preference, the four varieties are statistically different (*H* (3, 752) = 150,073 *p* < 0.0001). ‘Blegette’ and ‘Chandler’, the medium-sized walnut varieties, are chosen more frequently as the first, while ‘Bleggiana’ (the smallest) and ‘Lara’ (the largest) are less appreciated in that order. This result demonstrates how the Italian consumer seems to disagree with considering the walnut size a first quality requirement that drives the value at the sale: ‘large walnut higher price’ as reported by Mc Neil et al. [75].

## 3. Materials and Methods

### 3.1. Genetic Characterization

#### 3.1.1. Plant Material

Genetic characterization was performed on 33 *Juglans regia* L. accessions, which included the Italian varieties ‘Bleggiana’ and *local Franquette* (later ‘Blegette’), together with a wild accession, all sampled in Trentino on the Bleggio plateau, the Italian ecotypes of Feltre (2) and Sorrento (6), and 22 of the most important commercial walnut varieties from the USA and France (Table S1). The selection was made in order to evaluate the genetic diversity of the local varieties ‘Bleggiana’ and *local Franquette* (later ‘Blegette’) and to better understand their relation to the other local Italian accessions and the international commercial varieties. In total, 42 individuals were genotyped by means of SSR and SNP markers.

#### 3.1.2. SSR and SNP Genotyping

For microsatellites genotyping, young leaves of each accession were collected, each sample was lyophilized, and DNA was extracted with DNeasy Plant Kit (Qiagen, Germany) following the manufacturer’s instructions with the addition of 5 μL/sample of Proteinase K (Qiagen, Hilden, Germany) to the lysis solution. The extracted DNA was quality checked with a Nanodrop ND-8000 (ThermoScientific, Waltham, MA, USA) and diluted to 10ng/μL. Multiplex polymerase chain reactions (PCRs) were performed using 11 pairs of fluorescently labeled primers, divided into five reactions; the eleven SSR markers (Table 3) were chosen from the literature [19,40,42,76] on the basis of polymorphism and amplification success, after initial testing on our samples. The quality of the allele calls was assessed by comparison with the published profiles [19,40,42,76]. PCRs were performed in a final volume of 12.5 μL, with 0.2 μM of each primer and 0.64X of 2X Type-it Multiplex PCR Master Mix (Qiagen, Hilden, Germany) and 2 μL of each sample.

PCR amplification protocol for M1, M3 and M4 multiplex was the same as Ruiz-Garcia et al. [76], with few modifications, and it consisted of an initial step of 5 min at 95 °C, 35 cycles of 45 s at 95 °C, 45 s at 58 °C and 45 s at 72 °C, followed by a final step of 10 min at 72 °C. The annealing temperature was reduced by 0.2 °C per cycle for the next 14 cycles, while for the last 20 cycles, the annealing temperature was 55 °C. For M2A, M2B and M5 multiplex, the thermal cycle was as follows: initial denaturation step at 95 °C for 5 min followed by 30 cycles at 95 °C for 30 s, 55 °C for 90 s, 72 °C for 30 s and a final extension at 60 °C for 30 min. PCR products were diluted to 1:50, and 1 μL of the dilution was mixed with 8.95 μL Hi-Di formamide (Thermo Fisher Scientific, Waltham, MA, USA) and 0.05 μL Genescan 500 LIZ size standard (Thermo Fisher Scientific, Waltham, MA, USA). Samples were denatured at 95 °C for 15 min before being analyzed with an ABI 3730 XL sequencing system (Thermo Fisher Scientific, Waltham, MA, USA). Electropherograms were analyzed using the software GeneMapper4.0 (Thermo Fisher Scientific, Waltham, MA, USA) to size each fragment.

SNP genotyping was performed as described in Marrano et al. [41]; briefly, young leaves were collected, freeze dried, pulverized, and DNA was extracted with E-Z 96 Plant DNA Kit (Omega Bio-tek; Norcross, GA, USA) with few modifications. The accessions were genotyped using the Axiom^TM^ *J. regia* 700K SNP array (Affymetrix GenTitan platform); clustering and SNP calling were processed with the Affymetrix^®^Genotyping Console^TM^ software (GTC, v4.2). Only the 141,231 robust Poly High Resolution (PHR) SNPs, identified as described in Marrano et al. [41], were used in the current study for genetic characterization.

#### 3.1.3. Analysis of Genetic Diversity

Data analyses were performed in the R environment (R Core Team 2021) using the *poppr 2.9.3* [77,78] and *ape 5.5* [79] R packages.

Genetic diversity estimates, including total number of observed alleles (N), private alleles, expected heterozygosity (H_exp_) [80], Shannon diversity index (H) [81] and evenness (E5) [82], were calculated for each SSR locus. Additionally, a genotype accumulation curve was produced using the function ‘genotype_curve’ in *poppr* [78]. A random sampling (*n* = 10,000) of loci was performed to create the distribution; then, the number of multi-locus genotypes (MLG) for an increasing number of SSRs (from 1 to *n*–1, where *n* is the maximum number of markers) was counted. The curve reached a plateau, indicating the minimum number of markers covering all the genetic variance among samples, so that adding more markers to the analysis would not create many new genotypes.

The genetic relationships among the 33 *J. regia* accessions were assessed using the neighbor-joining (NJ) method of cluster analysis [77,83]. Dissimilarities were based on Bruvo’s distance matrix for the SSR dataset [77,84] and on Prevosti’s genetic distance for the SNP datasets [78,85]. The unrooted NJ trees produced were drawn with FigTree (v1.4.3) [86].

### 3.2. Walnut Metabolite and Volatile Compounds Analysis

#### 3.2.1. Plant Material

Walnut samples were collected from mature trees grown in two nearby orchards in the Bleggio plateau (628 m a.s.l.) under a temperate climate with the same agronomic management. In-shell nut samples were obtained from three walnut varieties: the autochthonous ‘Bleggiana’ and ‘Blegette’, and one commercial cultivar, ‘Lara’. For each variety, all the plants selected for the collection of samples were screened and their identity validated (Table S2) by means of 11 SSR markers (Table 2). The in-shell nuts fallen to the ground after tree shaking were collected at harvest time, when 90% of the nut hulls split. The collections were carried out in 2017, 2018 and 2019, between the end of September and the beginning of October. Five trees for each of the three varieties were sampled (20 walnuts/tree), for a total of 15 samples, every year. Nutritional and organoleptic properties of the three walnut varieties were investigated for the raw kernel. Right after harvest, all nuts were manually cracked and shelled, and the whole kernels of 10 walnuts were pooled and frozen in liquid nitrogen. Subsequently, the samples were ball milled into a fine powder using Mixer Mill MM400 homogenizer (Retsch GmbH, Haan, Germany) and stored at −80 °C until analysis.

#### 3.2.2. Analysis of Phenolic Compounds and Ellagitannins in Walnut Whole Kernels

The measurement of phenolic compounds was performed by ultraperformance liquid chromatography coupled with tandem mass spectrometry (UPLC-MS/MS).

Phenolics and ellagitannins extraction procedure was carried out according to Vrhovsek et al. [87]. Briefly, 0.4 mL of chloroform and 0.6 mL of methanol/water (2:1) were added to 0.1 g of each sample; a 20 µL aliquot of an internal standard solution (gentisic acid and rosmarinic acid) was added for monitoring the extraction and analytical protocols. The mixture was stirred for 15 min in an orbital shaker, centrifuged for 5 min at 15,000× *g* at 4 °C and the aqueous phase collected. The extraction process was repeated by adding 0.6 mL of methanol/water (2:1). The upper phases from the two extractions were combined. The obtained solution was then evaporated under nitrogen flow and re-suspended with 0.5 mL of methanol/water (2:1) and transferred into a vial for high-performance liquid chromatography (HPLC).

Ultraperformance liquid chromatography was performed on a Waters Acquity UPLC system (Milford, MA, USA), consisting of a binary pump, an online vacuum degasser, an auto-sampler and a column compartment. Separation of the phenolic compounds was achieved on a Waters Acquity HSS T3 column 1.8 μm, 100 mm × 2.1 mm (Milford, MA, USA), kept at 40 °C. Mass spectrometry detection was performed on a Waters Xevo TQMS (Milford, MA, USA) instrument equipped with an electrospray (ESI) source. Compounds were identified based on their standard and quantified using a calibration curve for each standard.

The measurement of ellagitannins was performed by using a high-performance liquid chromatograph coupled with an ultraviolet-visible (UV-VIS) detector and a single quadrupole mass spectrometer (HPLC-DAD_MS), following the instrumental conditions reported in Gasperotti et al. [88]. HPLC analysis was carried out using a Waters Alliance 2690 HPLC system equipped with a Waters 2996 DAD and qDA mass spectrometer (Waters Corp., Milford, MA, USA). The ellagitannins were identified by UV-VIS spectrum and mass spectrum based on the compounds reported in the literature. The quantification was performed as equivalents of gallic acid or ellagic acid using calibration curves.

#### 3.2.3. Nuclear Magnetic Resonance (NMR) Analysis of Lipid Profiles

Sample extraction followed the procedure described above for the quantification of lipids by UHPLC-MS/MS, until a dry extract was obtained. 1H-NMR spectra of the walnut lipid extracts were recorded in deuterated chloroform (CDCl_3_) at 300 K on two different nuclear magnetic resonance (NMR) spectrometers; organic extracts deriving from nuts collected in 2017–2018 were analyzed by Bruker-Avance (Bremen, Germany) operating at 400 MHz, while organic extracts deriving from nuts collected in 2019 were analyzed by Bruker-Avance (Bremen, Germany) operating at 600 MHz. In both cases, a 5 mm inverse broadband (BBI) probe with pulsed-gradient field utility was used, maintained at 300 K (±0.1 K) using a variable temperature unit (B-VT 1000; Bruker, Billerica, MA, USA). Recycle delay (d1) was set at 10 s to allow complete proton relaxation for quantitative purposes. Calibration of the chemical shift scale (δ) was carried out on the residual proton signal of the CDCl_3_ at δ_H_ 7.260 ppm. The resulting one-dimensional (1D) NMR spectra were analyzed using TopSpin 3.6.1 (Bruker, Bremen, Germany). The lipid classes from the NMR data were identified through comparisons with our previous NMR measurements carried out on commercially available lipid standards.

#### 3.2.4. Metabolic Data Analysis

The analysis of metabolomic data was performed in R. To stabilize the variance, all analyses of targeted assays were performed on log-transformed intensities. Data were manipulated and visualized by using *tydiverse* and *ggplot* packages [89]. Prior to a multivariate analysis, the missing values were imputed by using the knn algorithm provided by the *tidymodels* package [90]. PCA modeling on the scaled dataset was performed, relying on the *tidymodels*, *FactoMineR* and *factoextra* packages [90,91,92]. To remove the effect of the harvesting year on the metabolic data, all metabolites were mean centered by year.

NMR free induction decays (FIDs) from the two instruments were pre-processed by using the *PepsNMR* package [93]; spectra were aligned by using the CH_3_ triplet (t) at 0.95 ppm. Probabilistic quotient normalization (PQN) was applied to compensate for sample-to-sample variability. For each sample, the full NMR spectrum was used to calculate the amount of the most important lipid features.

The % molar fraction of α-linolenic acid (18:3, total ω-3 lipids) was obtained by the ratio of the peak area of ω-3 Me (δ_H_ = 0.97 t) with respect to the peak area of the signal at δ_H_≈2.30 broad triplet (brt), attributable to -CH_2_- group in α-position in each acyl chain. The % molar fraction of linoleic acid (18:2, total ω-6 lipids) was obtained by the ratio of the peak area of bis-allylic protons (δ_H_ = 2.77 brt) with respect to the peak area of the signal at δ_H_ ≈ 2.30 minus the contribution of the previously evaluated % molar fraction of α-linolenic acid (i.e., %18:2 = %PUFA-%18:3). The relative amount of MUFA (16:1 + 18:1, essentially) was evaluated by the peak area of allylic protons (δ_H_ = 2.01 brt) with respect to the peak area of the signal at δ_H_ ≈ 2.30 minus the contribution of the previously evaluated % molar fraction of PUFA. Finally, SFA (16:0 + 18:0 essentially) was evaluated from the total peak area of methyl protons (0.97 ≤ δ_H_ ≤ 0.89) with respect to the peak area of the signal at δ_H_ ≈ 2.30 minus the contribution of PUFA and MUFA. From these data, the average UI of the acyl chains in triglycerides (TAG) was also evaluated.

#### 3.2.5. PTR-ToF-MS Analysis of VOC Profiles

Five biological replicates of 1 g of powdered frozen sample were inserted into 20 mL glass vials equipped with PTFE/silicone septa (Agilent, Cernusco sul Naviglio, Italy) and mixed with 1 mL of deionized water with 400 mg of sodium chloride, 5 mg of ascorbic acid and 5 mg of citric acid [94]. Measurements of walnut VOCs were performed in three technical replicates with a commercial PTR-ToF–MS 8000 apparatus (Ionicon Analytik GmbH, Innsbruck, Austria). The drift tube conditions were as follows: 110 °C drift tube temperature, 2.25 mbar drift pressure, 550 V drift voltage. This led to an E/N ratio of about 140 Townsend (Td), with E corresponding to the electric field strength and N to the gas number density (1 Td = 10–17 Vcm^2^). The sampling time per channel of ToF acquisition was 0.1 ns, amounting to 350,000 channels for a mass spectrum ranging up to *m/z* = 400, which resulted in the acquisition rate of 1 spectrum/s. Each measurement was conducted automatically after 20 min of sample incubation at 40 °C by using an adapted gas chromatography (GC) autosampler (MPS Multipurpose Sampler, GERSTEL), and 1 min of time between each measurement was applied in order to prevent the memory effect. The sample headspace was withdrawn with the 2.5 mL syringe (CTC Analytics AG, Zwingen, Switzerland) and injected into the static headspace module (Ionicon Analytik GmbH, Innsbruck, Austria). The flow of zero air inside the static headspace module was 90 sccm, and the syringe was injected with the speed 250 µL/s, which provoked a 7-time dilution of the sample. An analysis time was 10 s/sample. Pure nitrogen was flushed through the syringe immediately before withdrawal to prevent the contamination of a measurement. PTR-MS performances were verified with certified calibration mixtures. Sensitivity was better than 10 cps/ppbv, and the LOD was lower than 100 pptv at an acquisition rate of 1 spectrum/s. Mass resolution was better than 4000 M/ΔM. We waited 1 min between samples to avoid any memory effect.

The analysis of PTR-ToF-MS spectra proceeded as described in Di Guardo et al. [62]. The array of masses detected with PTR-ToF-MS was reduced by applying noise and correlation coefficient thresholds, as follows: the first removed peaks that were not significantly different from blank samples; the latter excluded peaks with over 99% correlation, which mostly corresponded to isotopes of monoisotopic masses. The correction of the dilution was performed before further analysis. R.4.0.268 internal statistical functions and the external packages *mixOmics* [95], *heatmap3* [96] and *ggplot2* [97] were used for the multivariate statistical methods (PCA, heatmap, hierarchical clustering).

### 3.3. Consumer Study

**Figure 9:**
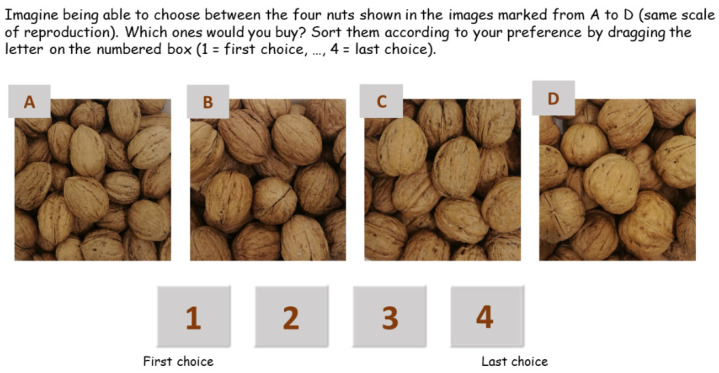
Screen used in the consumer study for evaluating walnut visual aspects. Consumers were asked to sort images of the four walnut varieties from the most preferred to least preferred on the basis of visual appearance. No information about the corresponding name of the cultivar was provided. A = ’Bleggiana’; B = ‘Chandler’; C = ‘Blegette’; D = ‘Lara’.

Within the current study, a consumer test was conducted in the Trento Expo exhibition space (Trento, Italy) on 27 and 28 October 2018 on ‘Fa la cosa giusta! 2018’, the critical consumption and sustainable lifestyles fair, inspired by ethics, sobriety and sustainability values. The responses of 188 consumers were collected in the Mobile Sensory Laboratory of Edmund Mach Foundation, compliant with the EN ISO standards 8589 (2014) [98], equipped with four mobile individual booths by using FIZZ 2.46A software (Biosystemes, Couternon, France). To explore and evaluate the consumers’ attitudes and preferences toward the locally cultivated walnut varieties, each consumer received four walnut samples (8 g of freshly shelled chopped dried kernels), one for each variety: ‘Bleggiana’, ‘Blegette’, ‘Lara’ and the most popular commercial variety ‘Chandler’. The samples were presented in plastic cups coded with random three-digit code according to a random and balanced order of presentation [99]. Consumers were asked to taste the samples in the presentation order and to rate their degree of overall liking on a 9-point scale (1 = Dislike extremely; 9 = Like extremely) under white light [100]. After tasting, participants rated their liking of the visual aspect for each walnut shell by evaluating the photographs that allowed perceiving the different varieties’ proportions (Figure 9).

In addition to socio-demographic data, participants provided information about their purchase, consumption habits and liking of different types of nuts, walnuts in particular. Furthermore, they assessed the appropriateness of consumption day moment, preparation method and the willingness to pay for an ‘organic’, ‘zero food miles’, ‘rich in ω-3’ or ‘first choice’ walnut.

None of the subjects were paid, and they voluntarily joined the test. Prior to participation, the experimental procedure was explained to all participants, and written informed consent was obtained from each, according to the European Data Protection Regulation (UE 679/2016). The consumers were asked to pay attention and to carefully read all the instructions provided during the test. Participants were provided with noise-proof earmuffs to help them concentrate in the noisy fair environment and asked to follow a rinse procedure with water and unsalted crackers to avoid possible carry-over effects between the products tested.

#### Statistical Analysis of Consumer Data

Data analysis was performed by STATISTICA v. 13.1 (Dell Inc., Tulsa, OK, USA, 2016).

In order to evaluate any hedonic difference between the four walnut varieties, the product effect (fixed factor) on overall acceptability was estimated using the one-way ANOVA [101] and Tukey’s honestly significant difference (HSD) [102] test for multi comparison. With the same aim, the corresponding non-parametric Kruskal–Wallis test [103] was used on walnut visual preference with multi-comparisons of mean ranks. Statistical differences were considered significant at *p* < 0.05.

## 4. Conclusions

The present work provides a comprehensive characterization of two traditional walnut varieties, cultivated in the Bleggio area of the Italian Alpine region of Trentino, where walnuts have been one of the typical products of the valley for centuries. The multidisciplinary approach applied in this study leads to several important outcomes. First of all, the genetic characterization confirms the genetic uniqueness of ‘Bleggiana’ and its identity as an autochthonous cultivar, and it reveals the presence of a second traditional cultivar, ‘Blegette’, initially thought to be more related to the French cultivars rather than to the local accessions. Both of these local cultivars show important nutritional content properties. In comparison to the commercial cultivar ‘Lara’, cultivated in the same area, ‘Bleggiana’ and ‘Blegette’ provide the highest intake of polyphenols. ‘Bleggiana’ also shows the best ω-6/ω-3 fatty acid ratio and the highest relative amount of ω-3 lipids. These are not absolute results, since metabolic constituents’ concentration is affected by the environment. However, concerning locally adapted varieties vs. an introduced commercial cultivar, our findings support a better nutraceutical role of the autochthonous and traditional walnut varieties. From the consumer point of view, although fruit sensory attributes, especially visual and taste properties, are among the most important, we report that the product quality, in terms of provenance, agronomic practices and nutritional value, is the main driver of preference, regardless of the cost increase. Concerning the walnut taste perception, consumer preference does not clearly discriminate among the cultivars tested. Additionally, the VOCs analysis reveals no differentiation among the cultivars studied, suggesting no clear differences in their aroma and flavors. Nevertheless, cultivation area, harvesting and production conditions can exert substantial influence on the product’s sensory quality.

In conclusion, this study underlines the great potential of a traditional and locally adapted germplasm, which deserves to be further addressed with future research on agronomically useful traits (i.e., biotic and abiotic stress tolerance) to set a promising starting point for a high-quality and sustainable local production.

Additionally, it would be worth to apply the approaches used in the present work to analyze the insight mechanisms of some important processes (e.g., ripening process, postharvest management and shelf-life) to improve the product quality.

Finally, we believe that studies like the one presented here can provide a scientifically sound approach to highlight the quality of a locally grown material.

## Figures and Tables

**Table 1:** Estimates of genetic diversity calculated for 11 SSR loci in 42 walnut genotypes. N: total number of observed alleles, H_o_: observed heterozygosity, H_exp_: expected heterozygosity, H: Shannon diversity index.

SSR Locus	N	H_o_	H_exp_	H	Evenness	Private Alleles ^1^
WGA005	10	0.69	0.82	1.85	0.79	267 bp: ‘Bleggiana’ 235 bp: Feltrina_1 271 bp: ‘Howard’
WGA032	6	0.62	0.51	0.97	0.62	172 bp: ‘Lara’
WGA069	6	0.60	0.70	1.33	0.79	163 bp: Feltrina_2
WGA089	4	0.29	0.58	0.97	0.82	
WGA118	3	0.71	0.60	1.00	0.86	
WGA202	4	0.88	0.67	1.22	0.83	
WGA276	7	0.76	0.71	1.45	0.71	181 bp: Feltrina_1
WGA321	6	0.80	0.72	1.49	0.73	
WGA331	4	0.40	0.59	0.98	0.83	269 bp: SoE_dub
WGA332	4	0.32	0.41	0.80	0.56	
WGA376	3	0.59	0.48	0.71	0.86	244 bp: ‘Cascade’
mean	5.18	0.61	0.62	1.16	0.76	

^1^ Size of a specific allele that is present in one accession only; SoE_dub = *Sorrento* ecotype 6.

**Table 2:** Acceptability mean values on tasting and the mean rank for visual preference for each variety with indication of homogeneous groups (different letters correspond to significantly different means with *p* < 0.05).

Variety	Mean Acceptability (SD *)	Visual Mean Rank
Bleggiana	6.55 (1.70) ab	408.5 b
Chandler	6.38 (1.72) b	263.5
Blegette	6.95 (1.47) a	321.5 a
Lara	6.84 (1.59) a	512.5 c

* Standard deviation.

**Table 3:** Microsatellite loci used to characterize the walnut genotypes.

SSR	References	Size Range (bp)	Dye	Multiplex
WGA005	[19,76]	235–273	FAM	M1
WGA032	[19,76]	166–198	FAM	M2A
WGA069	[40,42,76]	159–181	FAM	M3
WGA089	[40,42,76]	212–222	HEX	M2B
WGA118	[40,42,76]	185–199	HEX	M3
WGA202	[40,42,76]	261–277	FAM	M3
WGA276	[40,42,76]	167–193	HEX	M2A
WGA321	[40,42,76]	223–248	FAM	M2B
WGA331	[42,76]	269–277	HEX	M5
WGA332	[42,76]	215–227	FAM	M5
WGA376	[42,76]	244–256	HEX	M5

## Data Availability

All additional data supporting reported results are provided in the Supplementary Materials.

## Supplementary Materials

The following supporting information are available online at: https://www.mdpi.com/article/10.3390/plants11151986/s1, Figure S1: Genotype accumulation curve; Figure S2: Boxplot for each VOC mass peak assessed by PTR-ToF-MS in three harvesting seasons (2017, 2018 and 2019) for three walnut cultivars (‘Bleggiana’, ‘Blegette’ and ‘Lara’); Table S1: List of the 42 walnut plants included in the genetic characterization and relative information; Table S2: Allelic profiles (bp) at the 11 SSR loci; Table S3: Phenolics and ellagitannins compounds, quantified by ultraperformance liquid chromatography coupled with tandem mass spectrometry (UPLC-MS/MS), in the whole raw kernel; Table S4: VOC mass peaks significantly different among production years and accessions. File S1: SNP dataset file in .csv format, including the genotypes of 42 walnut plants for 141,231 SNPs. Affy ID of each genotyped individual, accession name and plant provenance are also reported.
