# Supplementary material for: Valorization of Traditional Italian Walnut (Juglans regia L.) Production: Genetic, Nutritional and Sensory Characterization of Locally Grown Varieties in the Trentino Region
Source: Plants (Basel). 2022 Jul 30;11(15):1986. doi: 10.3390/plants11151986 (PMC9370163; doi:10.3390/plants11151986)
Supplement: Supplementary file 1 [file plants-11-01986-s001.zip › Supplementary_Files/Figure_S1.pdf]

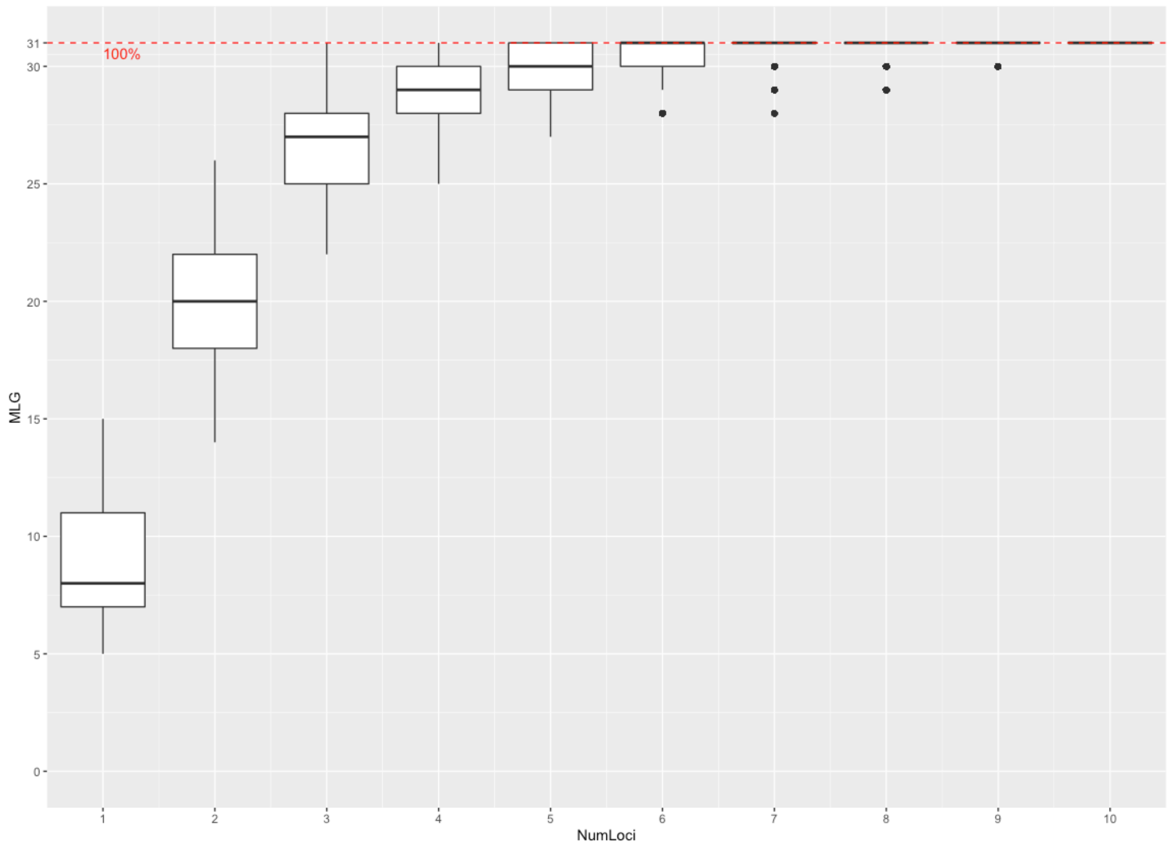

Figure S1. Genotype accumulation curve for 42 genotypes and 11 SSR loci produced using the function `genotype_curve` in *poppr* [78]. The horizontal axis represents the number of loci (NumLoci) randomly sampled without replacement up to n − 1 loci, the vertical axis shows the number of multilocus genotypes (MLG) observed, up to 31, the number of unique multilocus genotypes in the data set. The red dashed line represents 100% of the total observed multilocus genotypes. The curve reaches a plateau indicating the minimum number of markers covering all the genetic variance among samples, so that adding more markers to the analysis will not create very many new genotypes.
