# Supplementary material for: Valorization of Traditional Italian Walnut (Juglans regia L.) Production: Genetic, Nutritional and Sensory Characterization of Locally Grown Varieties in the Trentino Region
Source: Plants (Basel). 2022 Jul 30;11(15):1986. doi: 10.3390/plants11151986 (PMC9370163; doi:10.3390/plants11151986)
Supplement: Supplementary file 1 [file plants-11-01986-s001.zip › Supplementary_Files/Figure_S2.pdf]

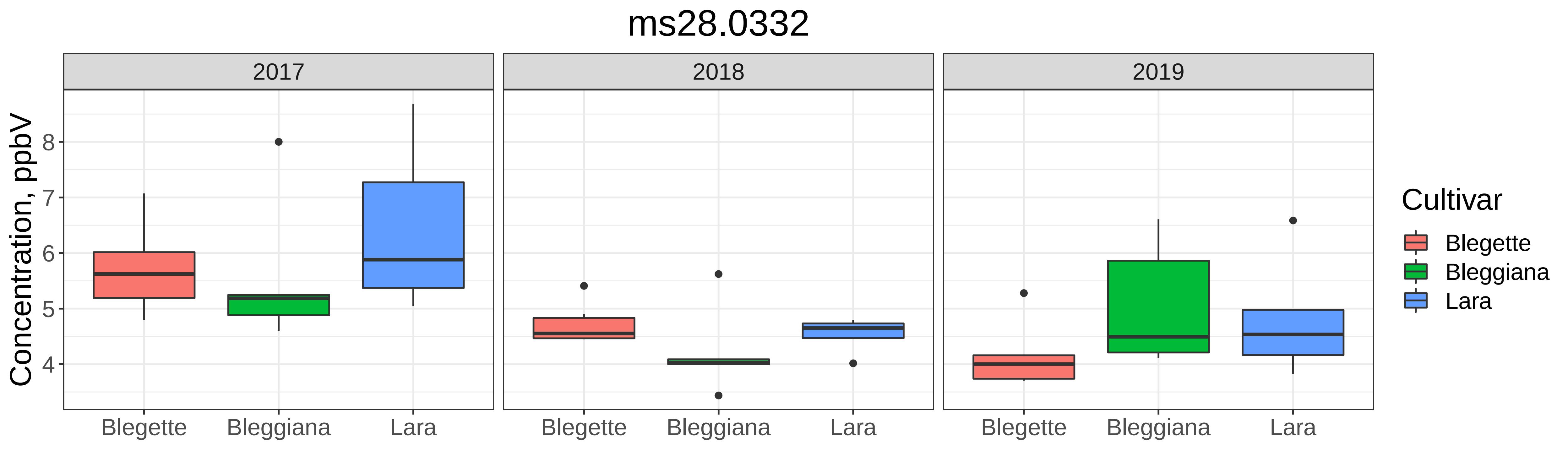

ms28.0332
2017
2018
2019
Concentration, ppbV
8
7
6
5
4
Blegette
Bleggiana
Lara
Cultivar
Blegette
Bleggiana
Lara

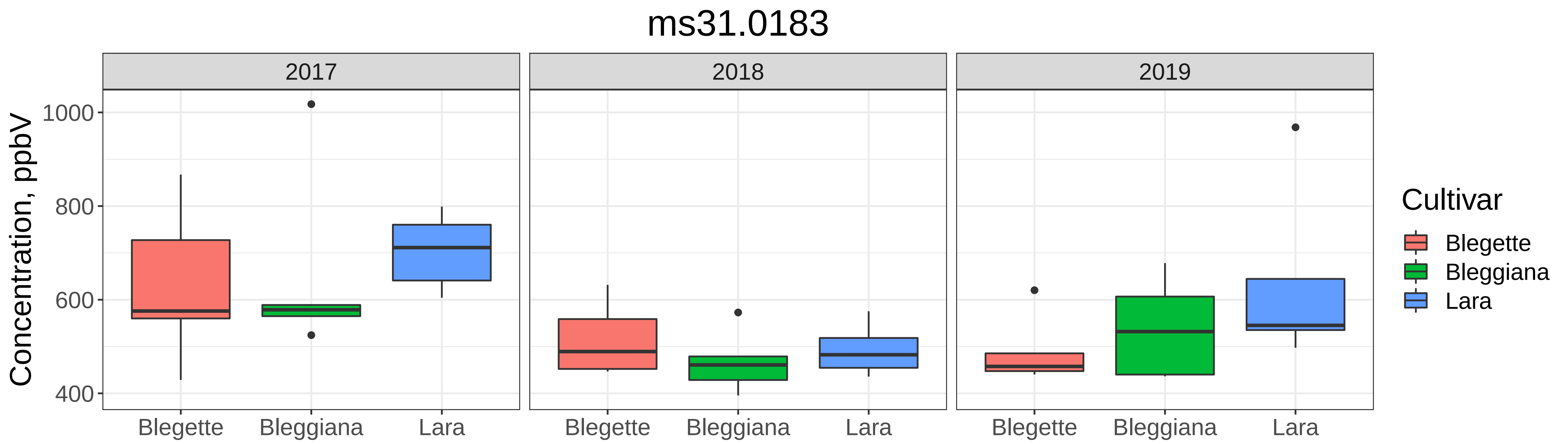

ms31.0183
2017
2018
2019
Concentration, ppbV
1000
800
600
400
Blegette
Bleggiana
Lara
Cultivar
Blegette
Bleggiana
Lara

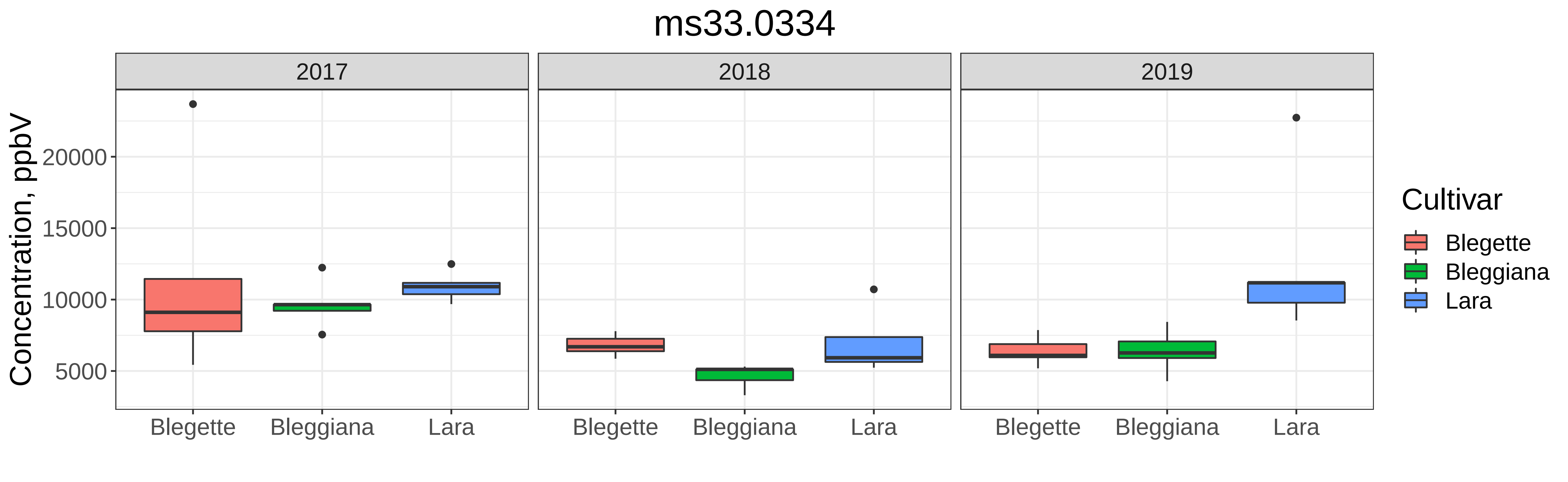

ms33.0334
2017
2018
2019
Concentration, ppbV
20000
15000
10000
5000
Blegette
Bleggiana
Lara
Cultivar
Blegette
Bleggiana
Lara

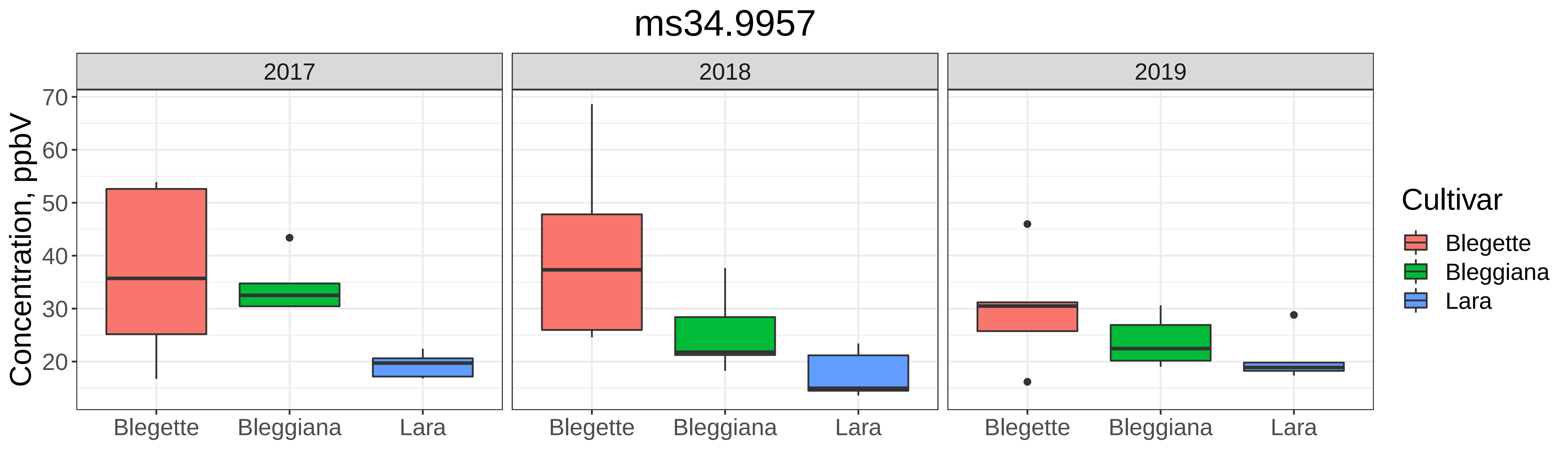

ms34.9957
2017
2018
2019
Concentration, ppbV
70
60
50
40
30
20
Blegette
Bleggiana
Lara
Cultivar
Blegette
Bleggiana
Lara

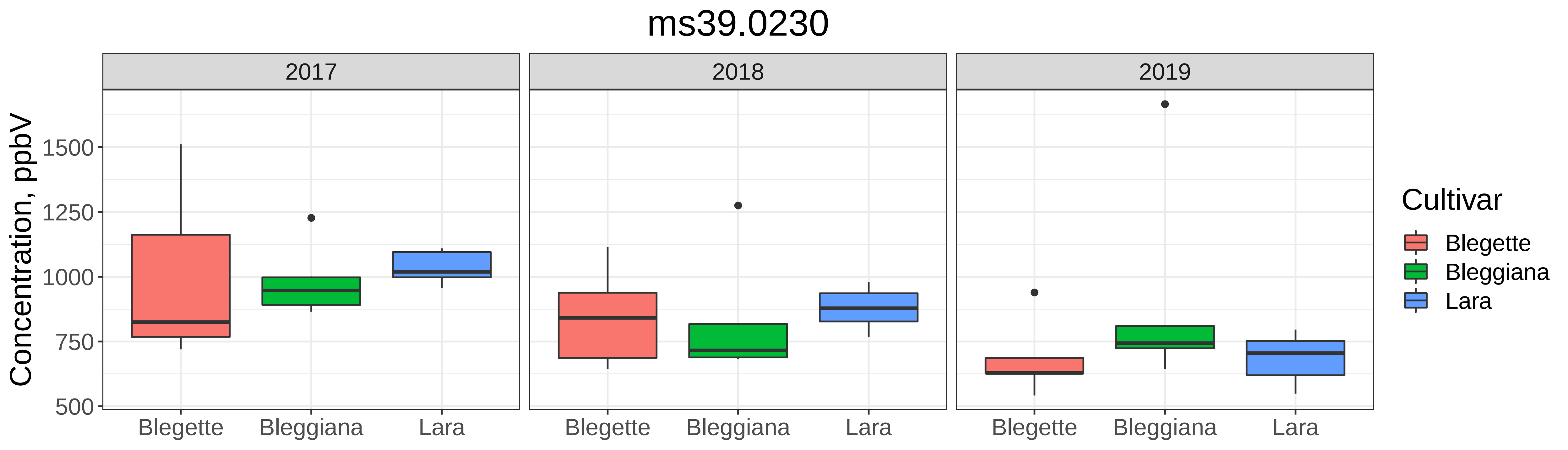

ms39.0230
2017
2018
2019
Concentration, ppbV
1500
1250
1000
750
500
Blegette
Bleggiana
Lara
Cultivar
Blegette
Bleggiana
Lara

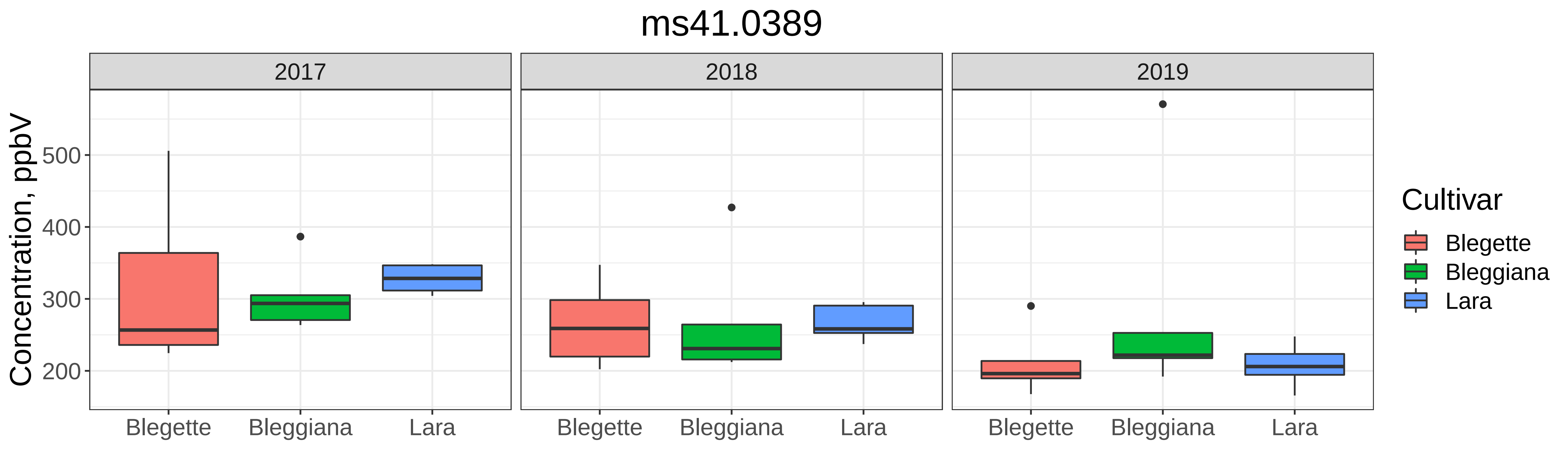

ms41.0389
2017
2018
2019
Concentration, ppbV
500
400
300
200
Blegette
Bleggiana
Lara
Cultivar
Blegette
Bleggiana
Lara

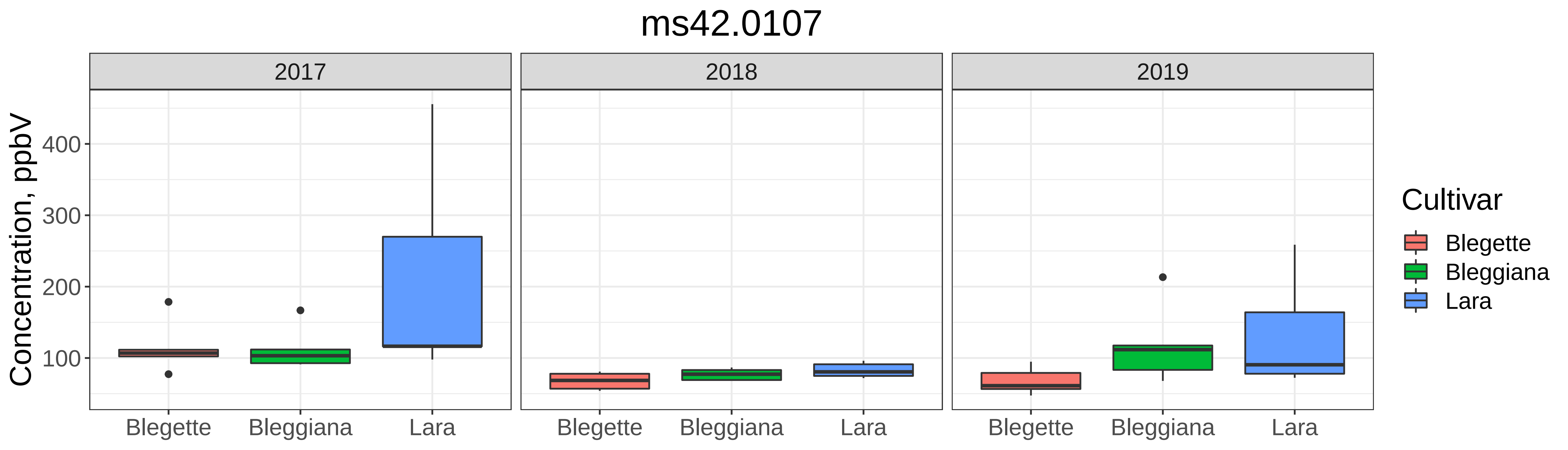
ms42.0107
2017
2018
2019
Concentration, ppbV
400
300
200
100
Blegette
Bleggiana
Lara
Cultivar
Blegette
Bleggiana
Lara

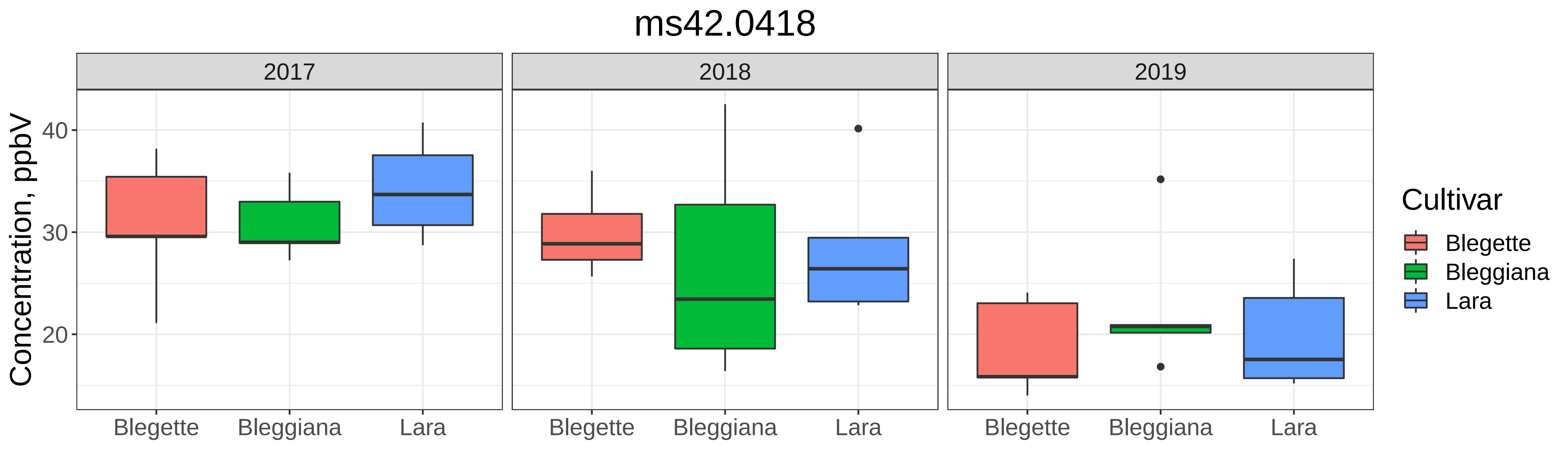
ms42.0418
2017
2018
2019
Concentration, ppbV
40
30
20
Blegette
Bleggiana
Lara
Cultivar
Blegette
Bleggiana
Lara

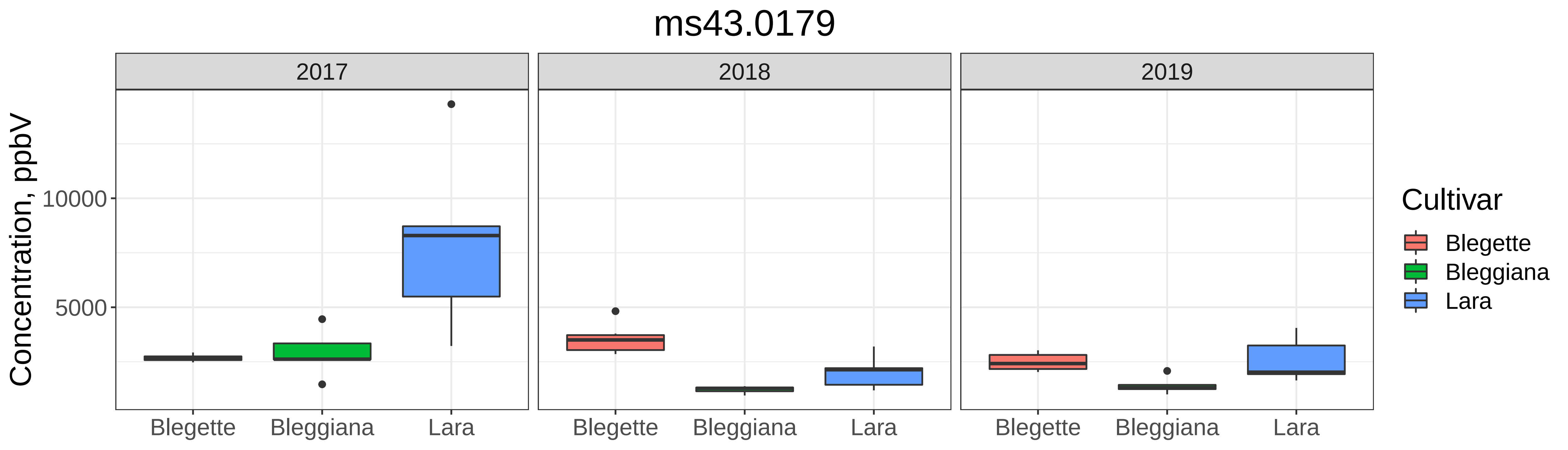

ms43.0179
2017
2018
2019
Concentration, ppbV
10000
5000
Blegette
Bleggiana
Lara
Blegette
Bleggiana
Lara
Blegette
Bleggiana
Lara
Cultivar
Blegette
Bleggiana
Lara

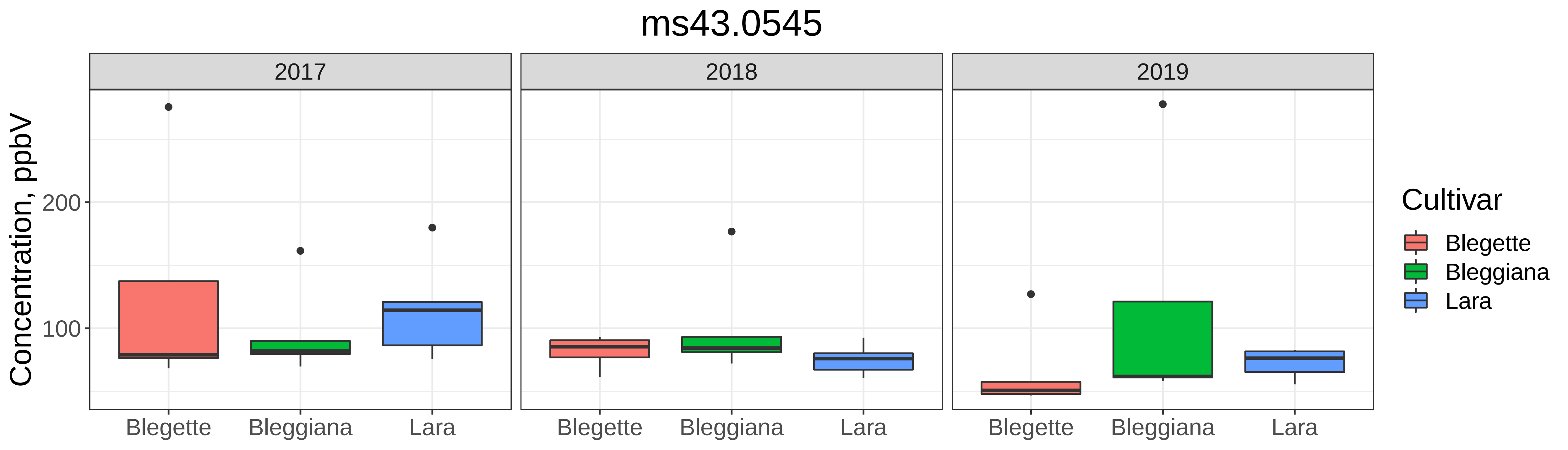

ms43.0545
2017
2018
2019
Concentration, ppbV
200
100
Blegette
Bleggiana
Lara
Cultivar
Blegette
Bleggiana
Lara

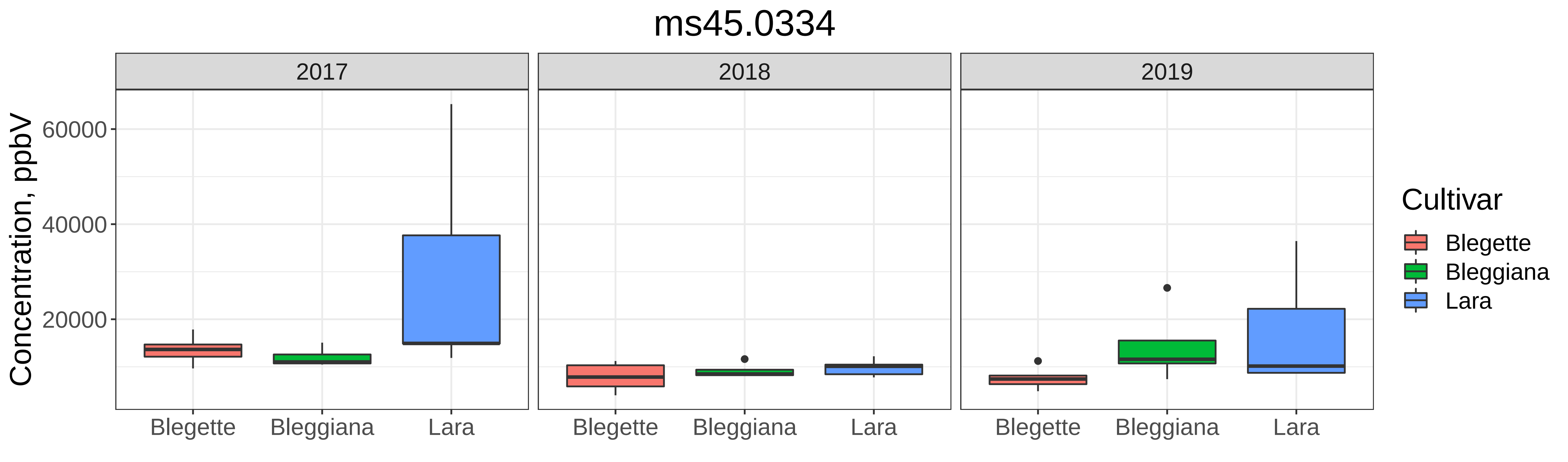

ms45.0334
2017
2018
2019
Concentration, ppbV
60000
40000
20000
Blegette
Bleggiana
Lara
Cultivar
Blegette
Bleggiana
Lara

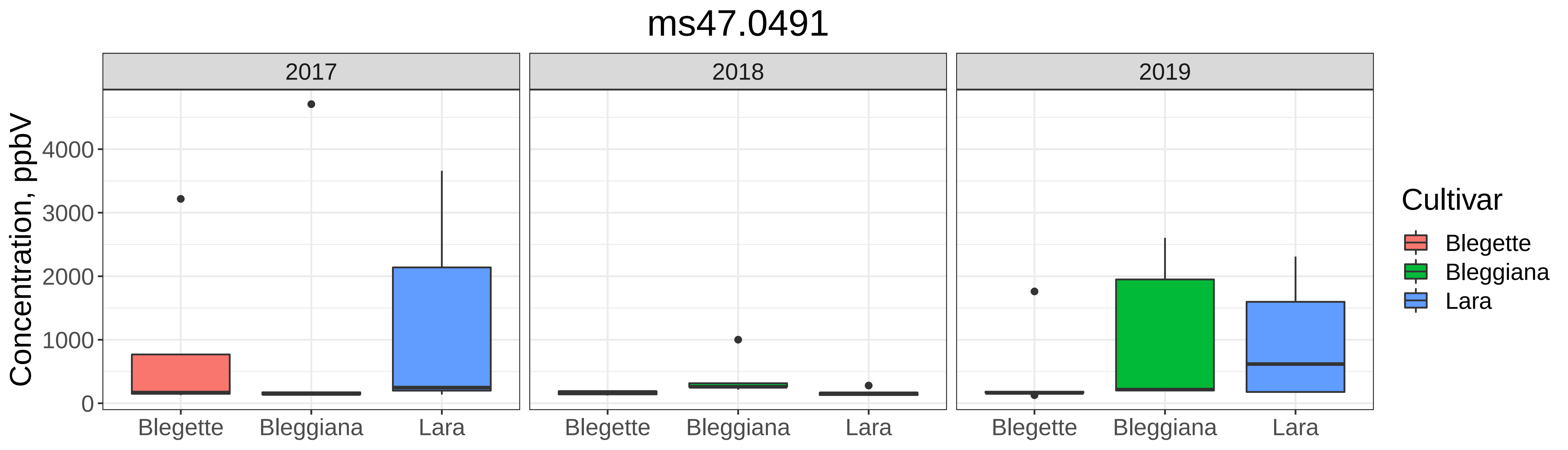

ms47.0491
2017
2018
2019
Concentration, ppbV
0
1000
2000
3000
4000
Blegette
Bleggiana
Lara
Cultivar
Blegette
Bleggiana
Lara

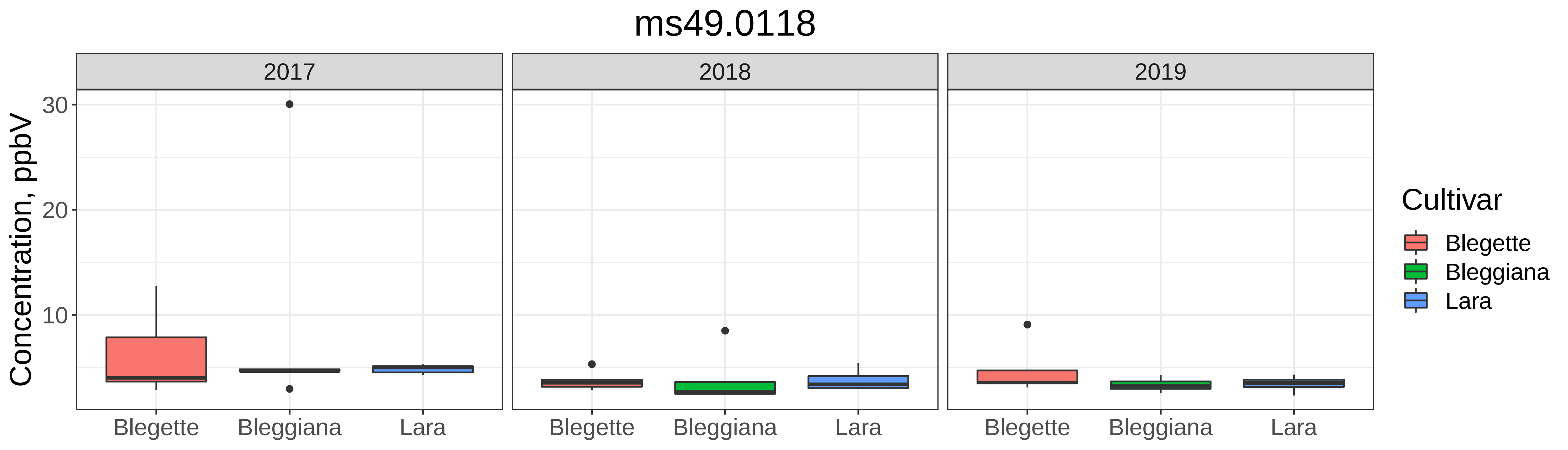

ms49.0118
2017
2018
2019
Concentration, ppbV
30
20
10
Blegette
Bleggiana
Lara
Cultivar
Blegette
Bleggiana
Lara

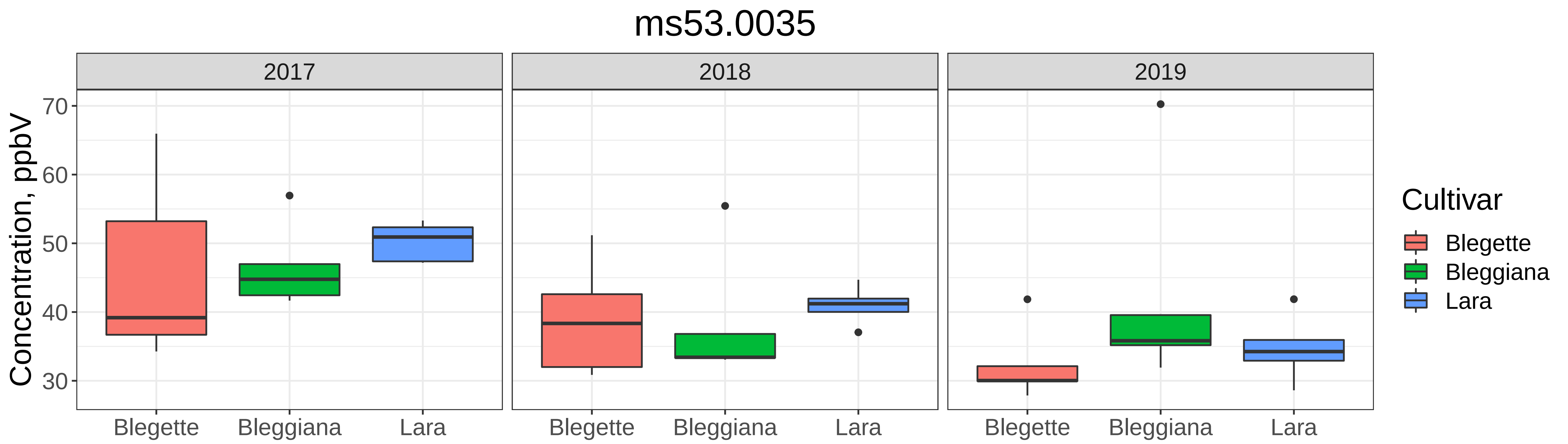
ms53.0035
2017
2018
2019
Concentration, ppbV
70
60
50
40
30
Blegette
Bleggiana
Lara
Cultivar
Blegette
Bleggiana
Lara

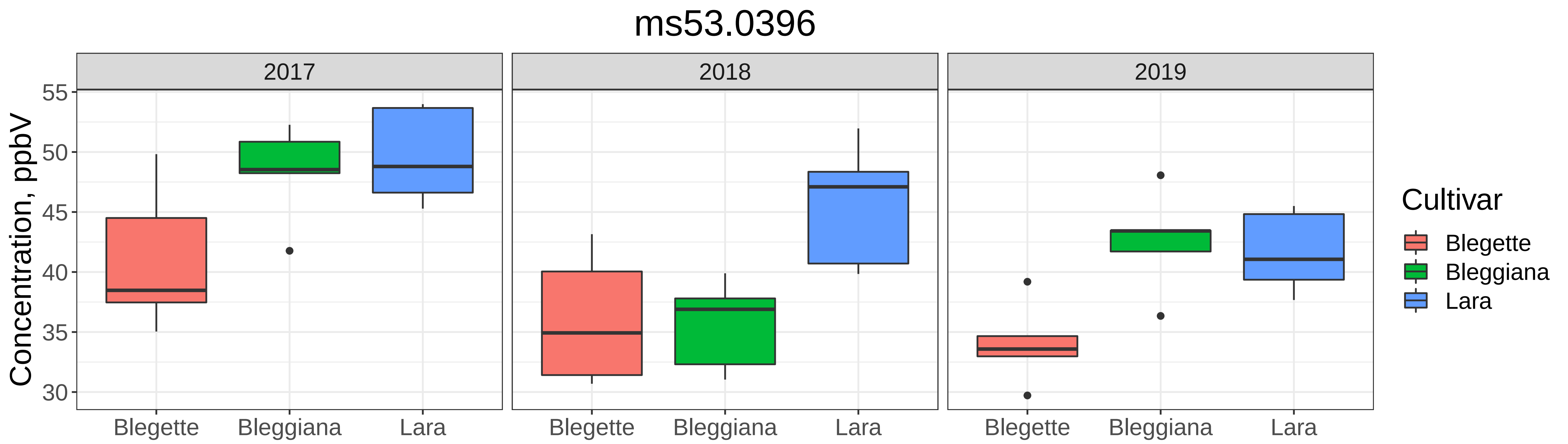
ms53.0396
2017
2018
2019
Concentration, ppbV
55
50
45
40
35
30
Blegette
Bleggiana
Lara
Cultivar
Blegette
Bleggiana
Lara

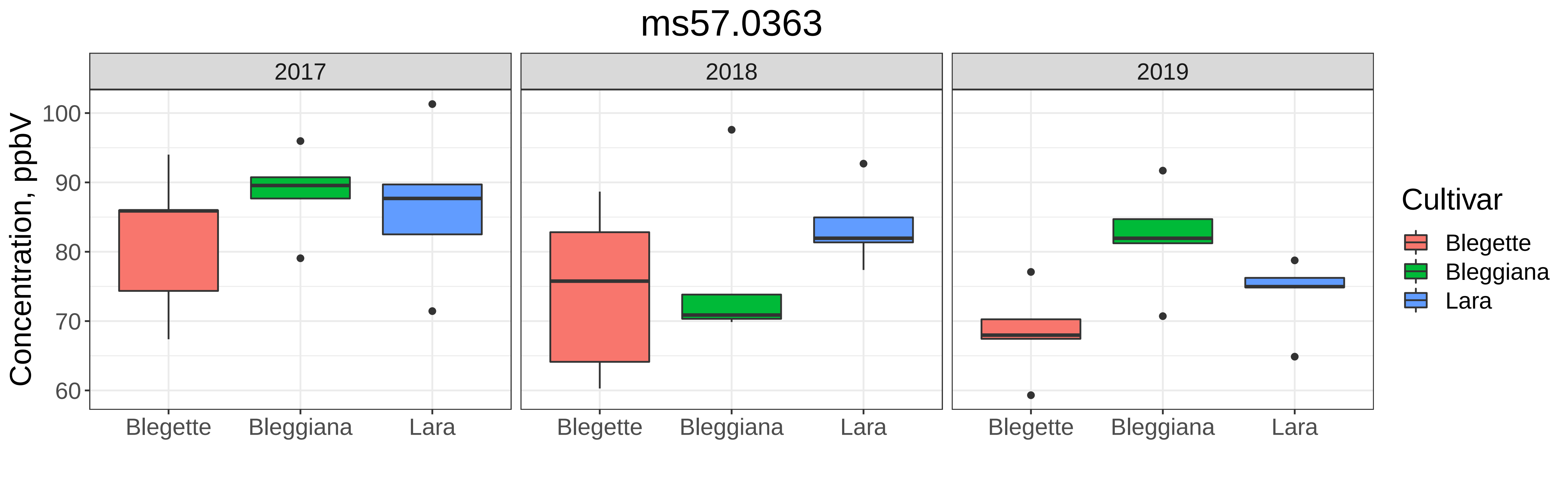
ms57.0363
2017
2018
2019
Concentration, ppbV
100
90
80
70
60
Blegette
Bleggiana
Lara
Cultivar
Blegette
Bleggiana
Lara

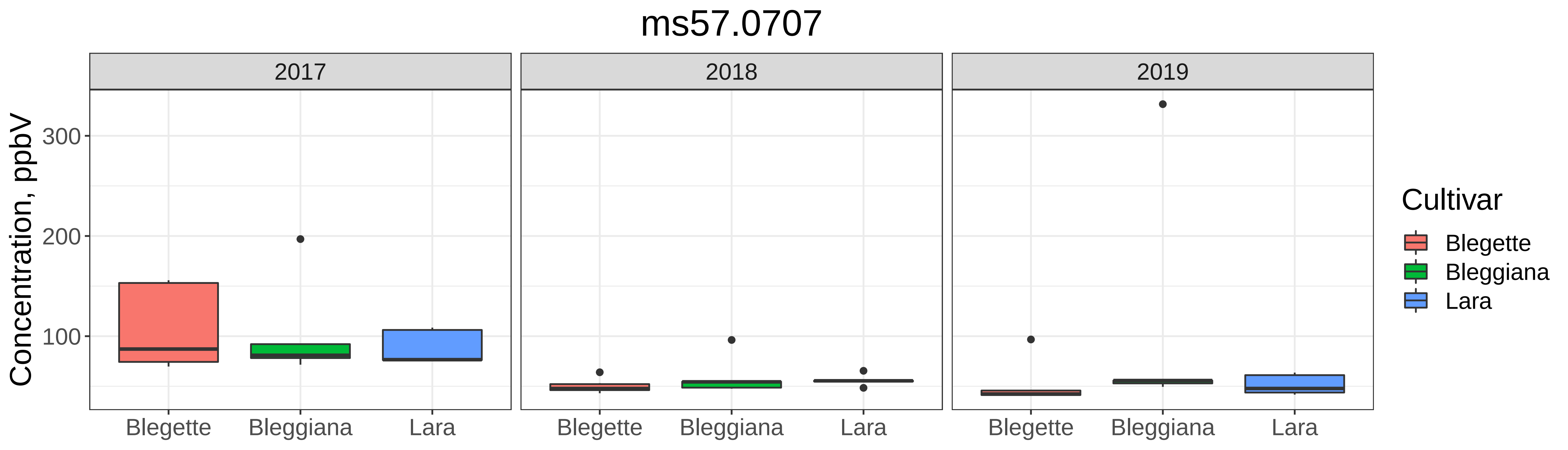
ms57.0707
2017
2018
2019
Concentration, ppbV
300
200
100
Blegette
Bleggiana
Lara
Cultivar
Blegette
Bleggiana
Lara

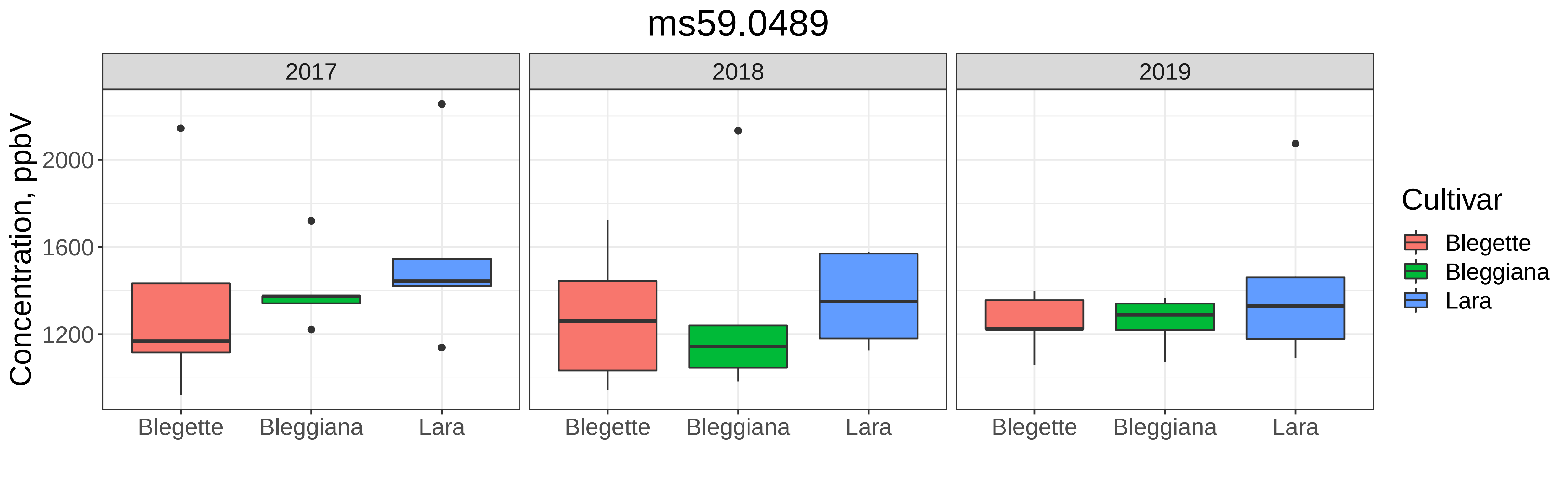

ms59.0489
2017
2018
2019
Concentration, ppbV
2000
1600
1200
Blegette
Bleggiana
Lara
Cultivar
Blegette
Bleggiana
Lara

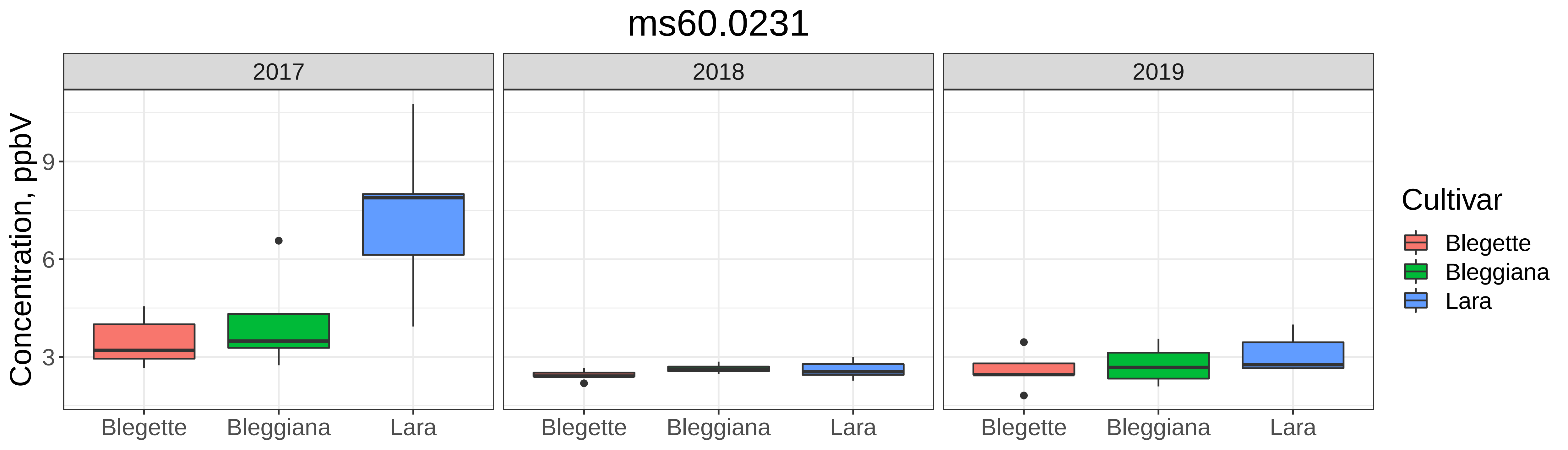
ms60.0231
2017
2018
2019
Concentration, ppbV
9
6
3
Blegette
Bleggiana
Lara
Cultivar
Blegette
Bleggiana
Lara

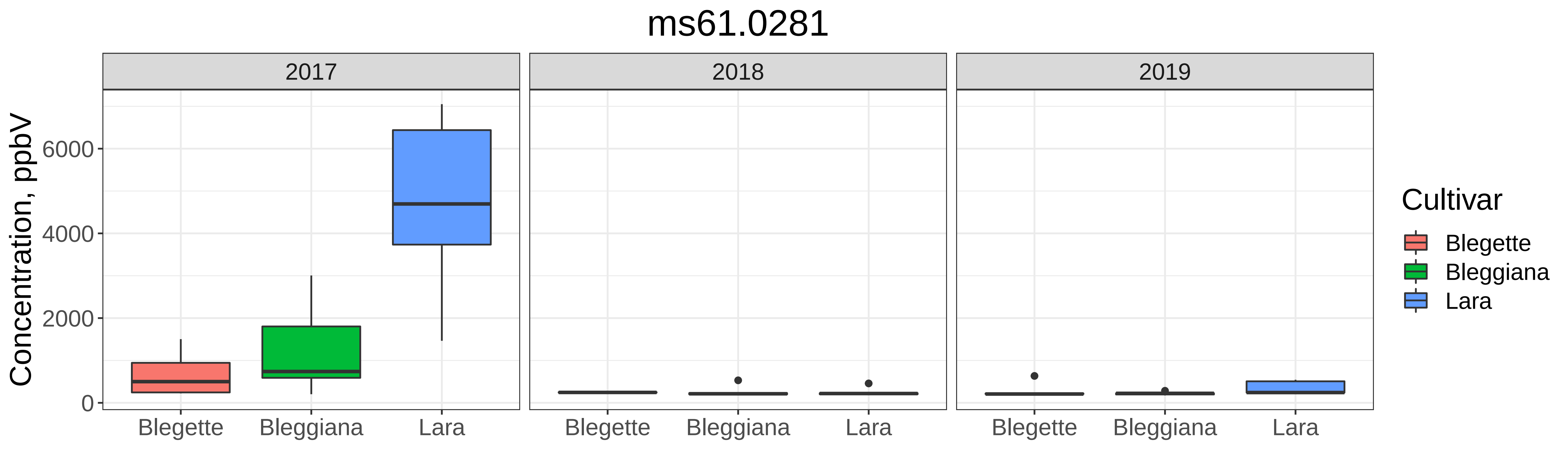

ms61.0281
2017
2018
2019
Concentration, ppbV
6000
4000
2000
0
Blegette
Bleggiana
Lara
Cultivar
Blegette
Bleggiana
Lara

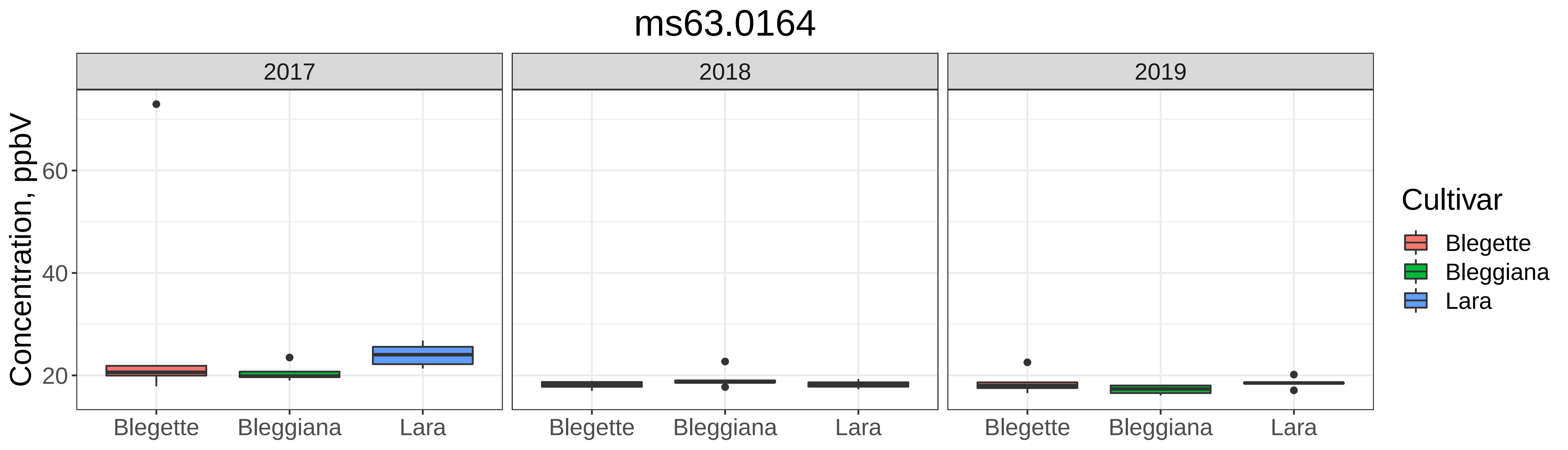
ms63.0164
2017
2018
2019
Concentration, ppbV
60
40
20
Blegette
Bleggiana
Lara
Cultivar
Blegette
Bleggiana
Lara

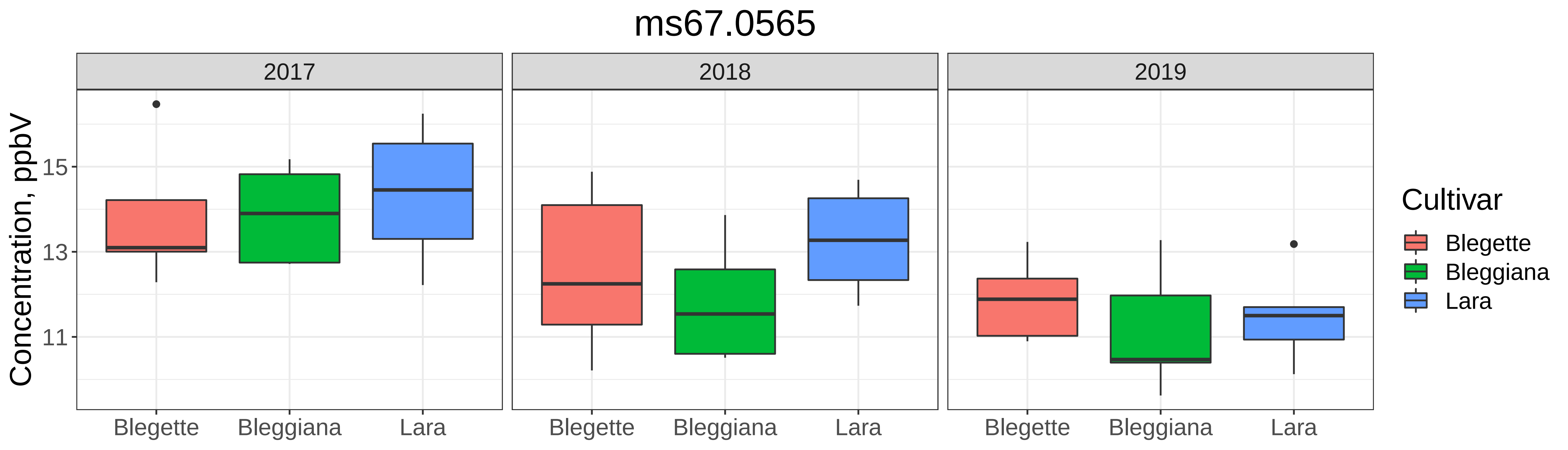

ms67.0565
2017
2018
2019
Concentration, ppbV
15
13
11
Blegette
Bleggiana
Lara
Cultivar
Blegette
Bleggiana
Lara

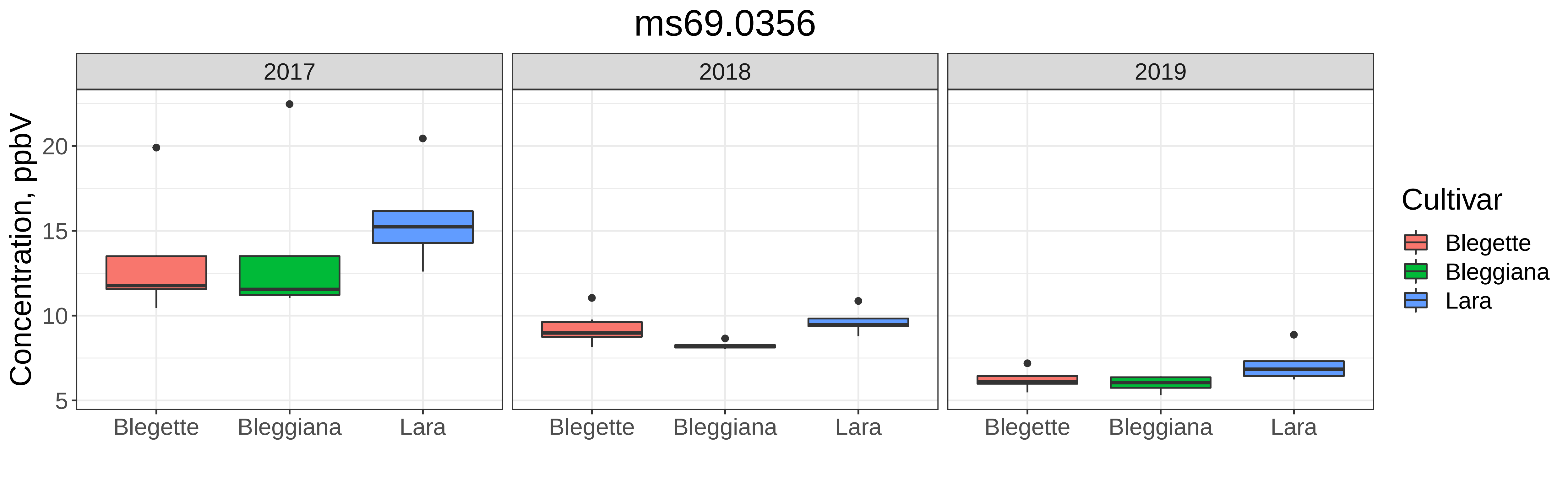

ms69.0356
2017
2018
2019
Concentration, ppbV
20
15
10
5
Blegette
Bleggiana
Lara
Cultivar
Blegette
Bleggiana
Lara

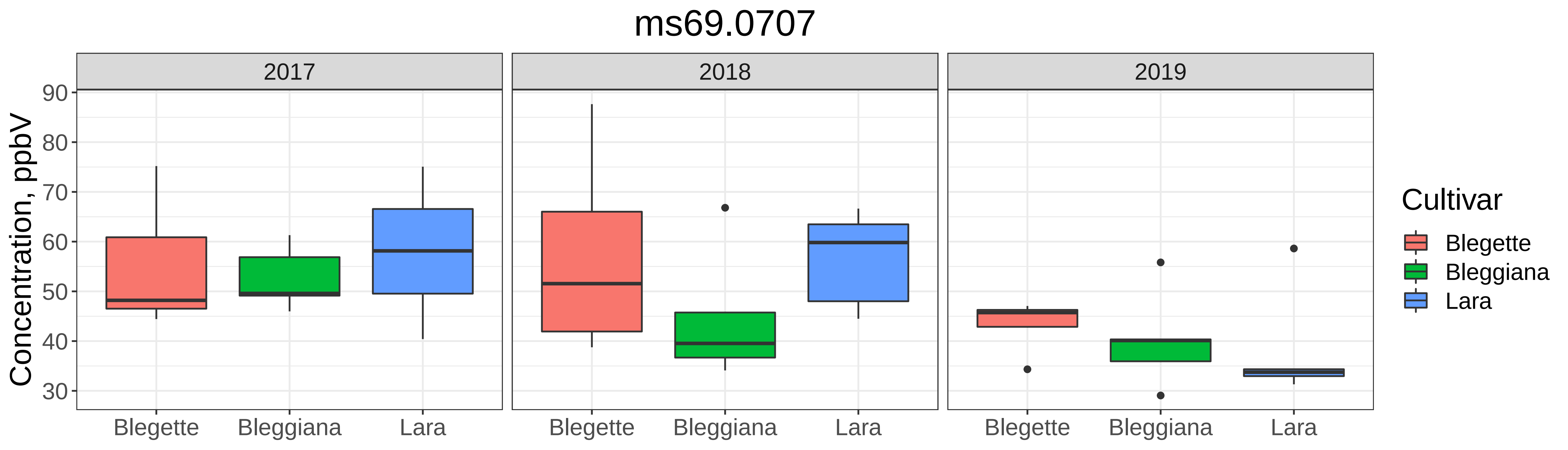

ms69.0707
2017
2018
2019
Concentration, ppbV
90
80
70
60
50
40
30
Blegette
Bleggiana
Lara
Cultivar
Blegette
Bleggiana
Lara

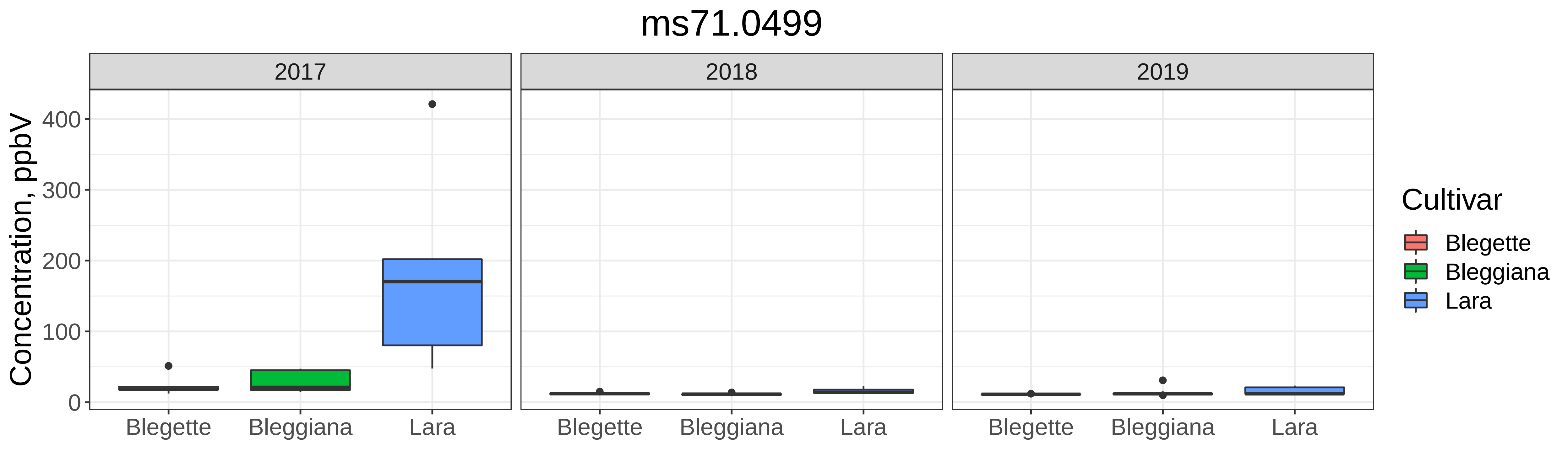

ms71.0499
2017
2018
2019
Concentration, ppbV
0
100
200
300
400
Blegette
Bleggiana
Lara
Cultivar
Blegette
Bleggiana
Lara

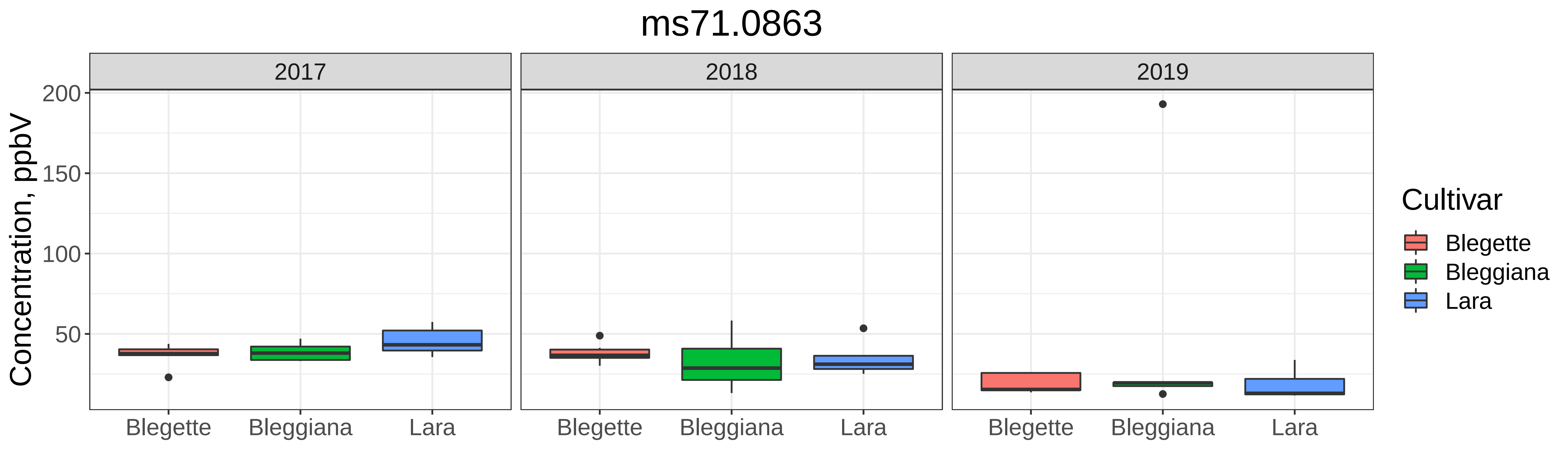

ms71.0863
2017
2018
2019
Concentration, ppbV
200
150
100
50
Blegette
Bleggiana
Lara
Cultivar
Blegette
Bleggiana
Lara

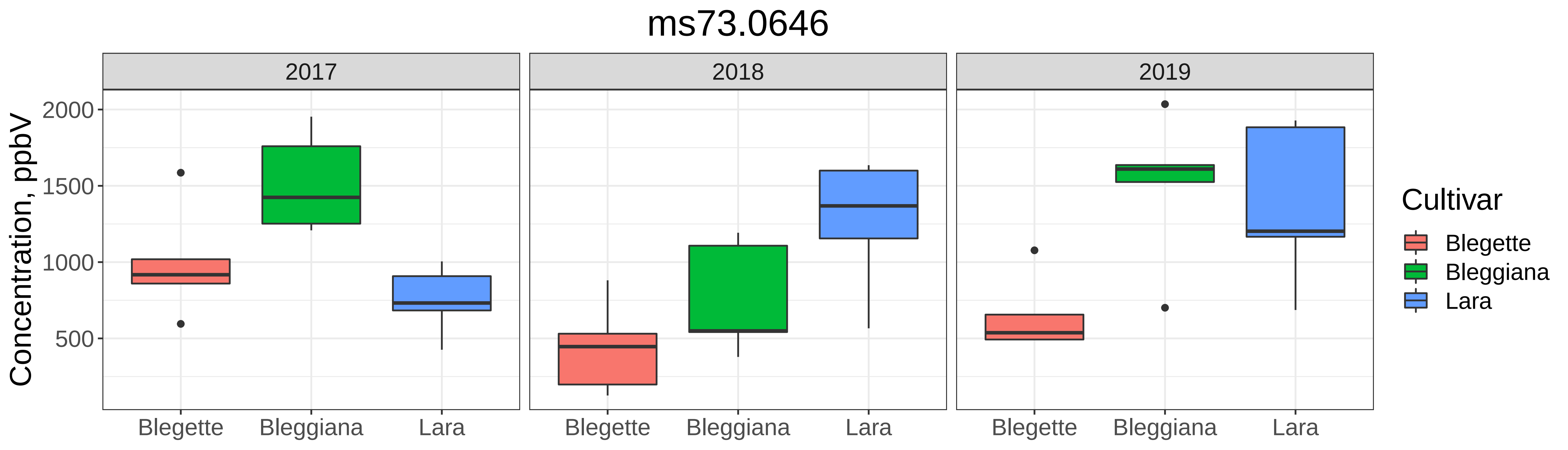

ms73.0646
2017
2018
2019
Concentration, ppbV
2000
1500
1000
500
Blegette
Bleggiana
Lara
Cultivar
Blegette
Bleggiana
Lara

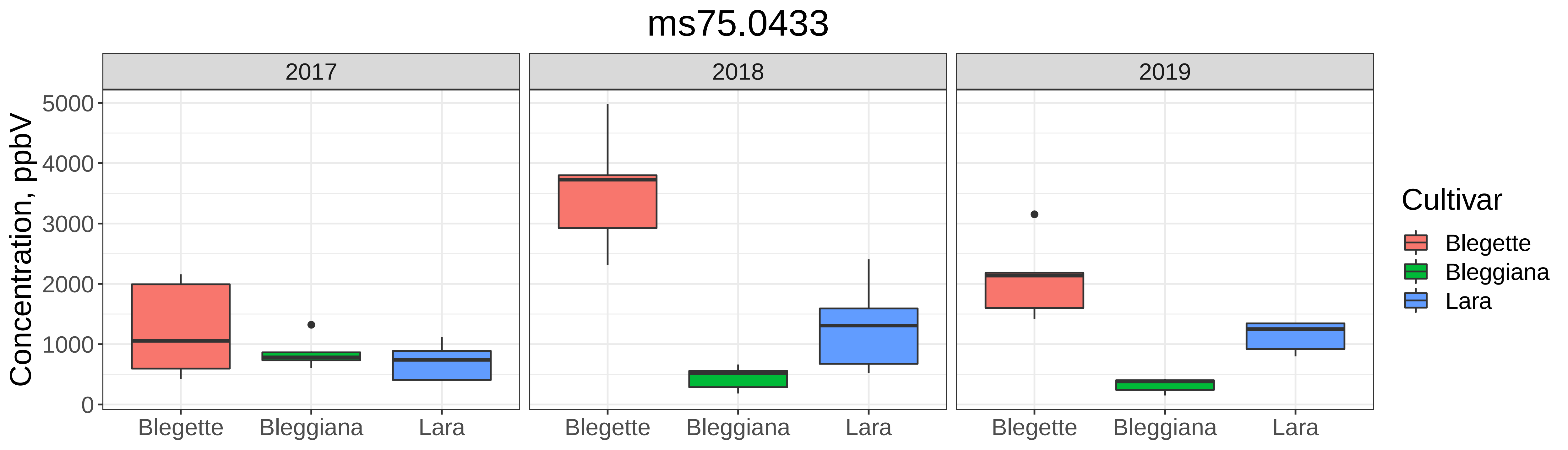
ms75.0433
2017
2018
2019
Concentration, ppbV
0
1000
2000
3000
4000
5000
Blegette
Bleggiana
Lara
Cultivar
Blegette
Bleggiana
Lara

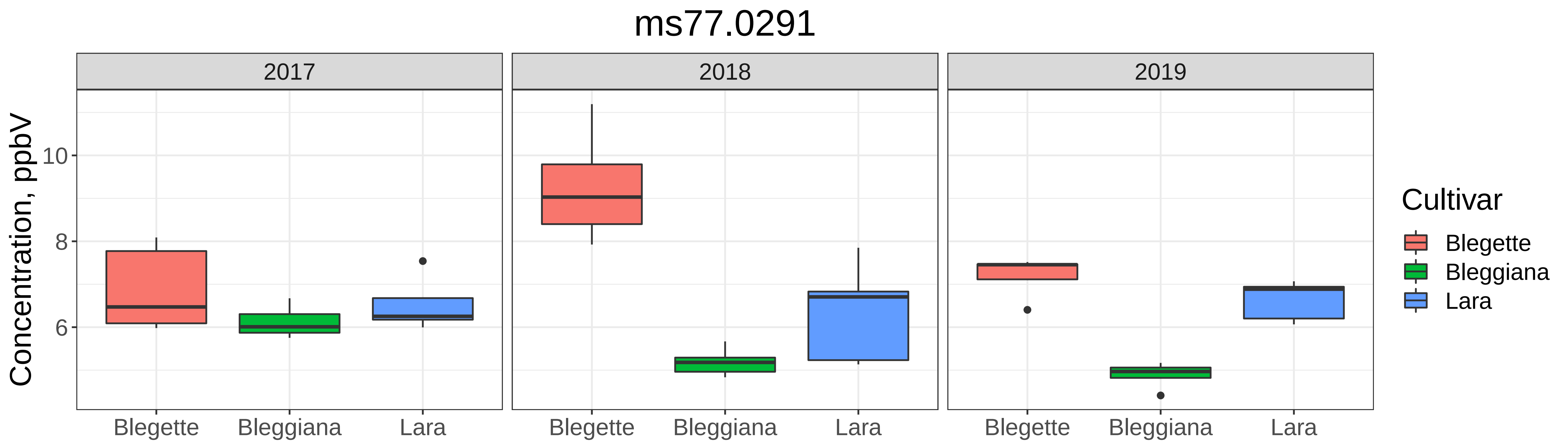
ms77.0291
2017
2018
2019
Concentration, ppbV
10
8
6
Blegette
Bleggiana
Lara
Cultivar
Blegette
Bleggiana
Lara

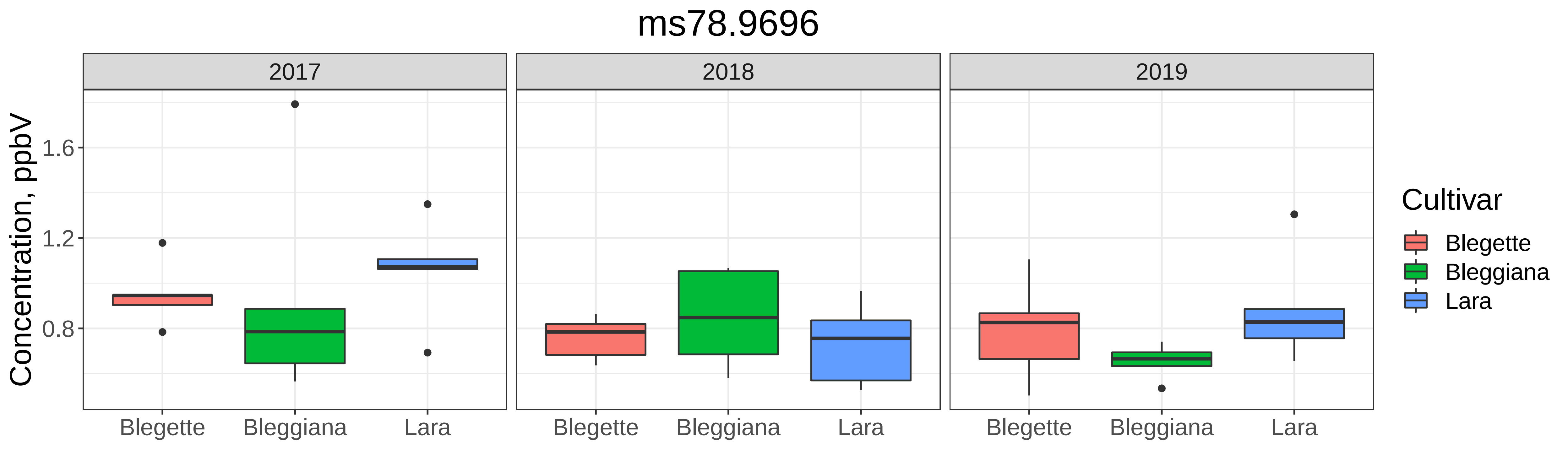

ms78.9696
2017
2018
2019
Concentration, ppbV
1.6
1.2
0.8
Blegette
Bleggiana
Lara
Cultivar
Blegette
Bleggiana
Lara

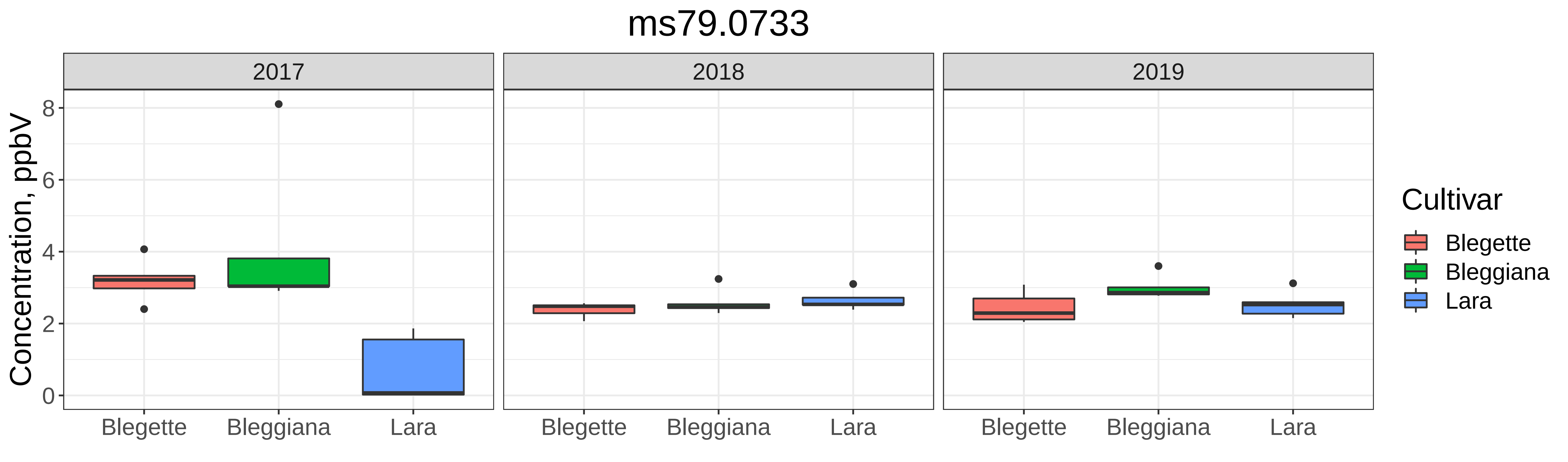

ms79.0733
2017
2018
2019
Concentration, ppbV
0
2
4
6
8
Blegette
Bleggiana
Lara
Blegette
Bleggiana
Lara
Blegette
Bleggiana
Lara
Cultivar
Blegette
Bleggiana
Lara

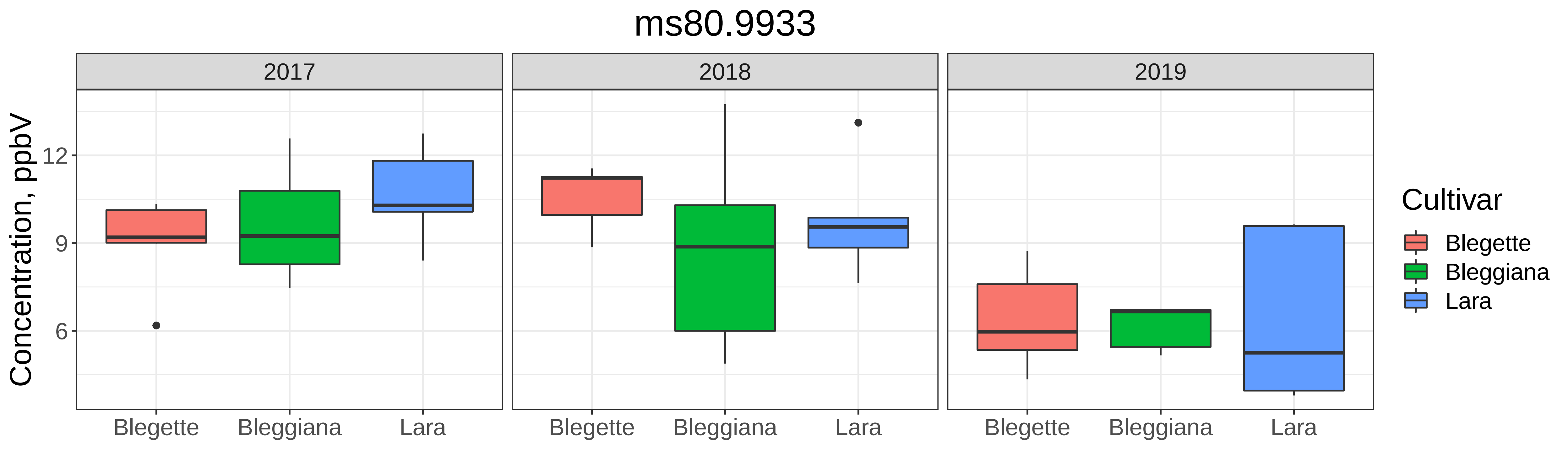

ms80.9933
2017
2018
2019
Concentration, ppbV
12
9
6
Blegette
Bleggiana
Lara
Cultivar
Blegette
Bleggiana
Lara

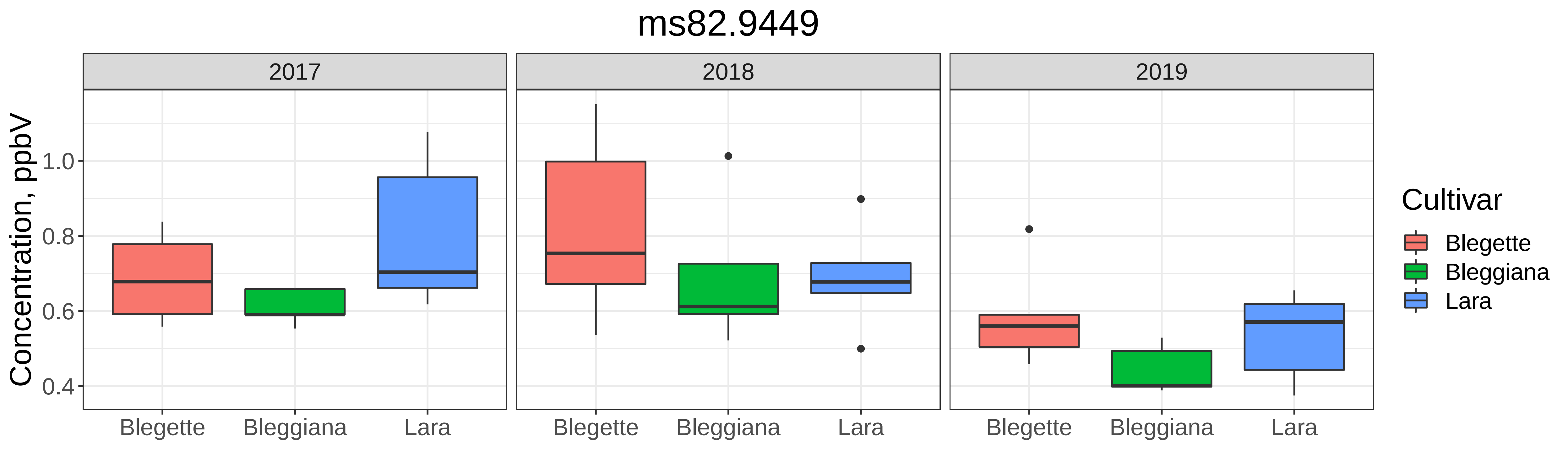
ms82.9449
2017
2018
2019
Concentration, ppbV
1.0
0.8
0.6
0.4
Blegette
Bleggiana
Lara
Cultivar
Blegette
Bleggiana
Lara

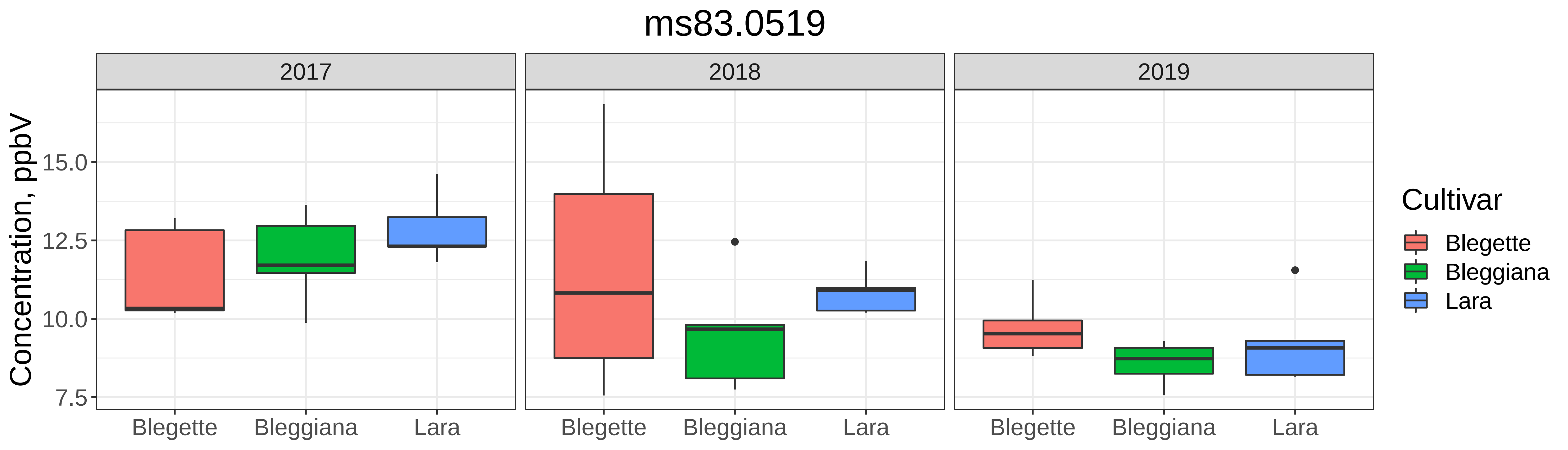

ms83.0519
2017
2018
2019
Concentration, ppbV
15.0
12.5
10.0
7.5
Blegette
Bleggiana
Lara
Cultivar
Blegette
Bleggiana
Lara

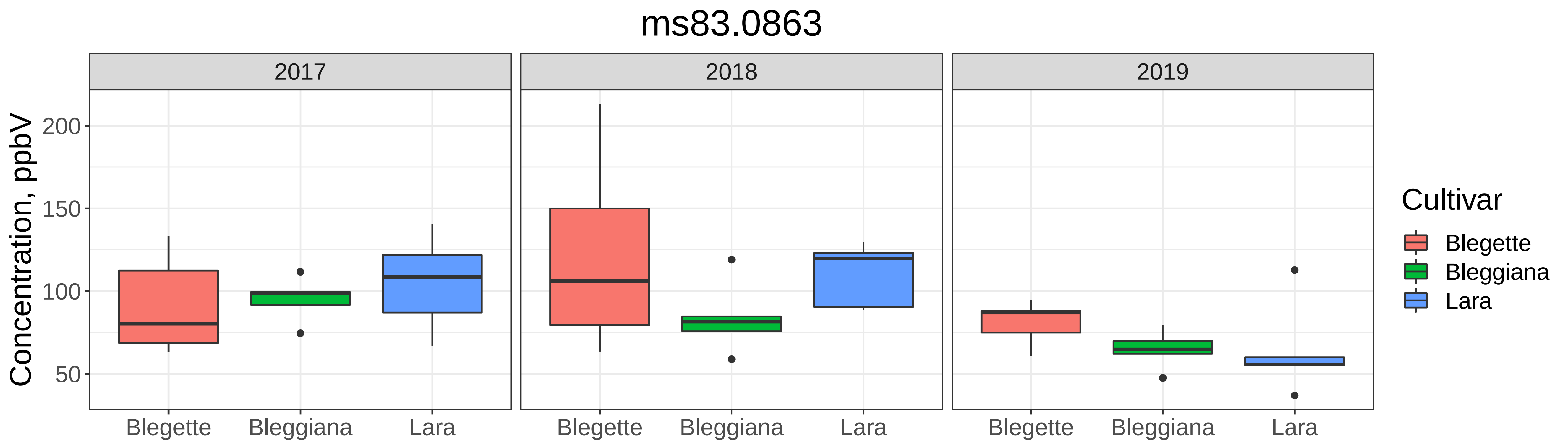

ms83.0863
2017
2018
2019
Concentration, ppbV
200
150
100
50
Blegette
Bleggiana
Lara
Cultivar
Blegette
Bleggiana
Lara

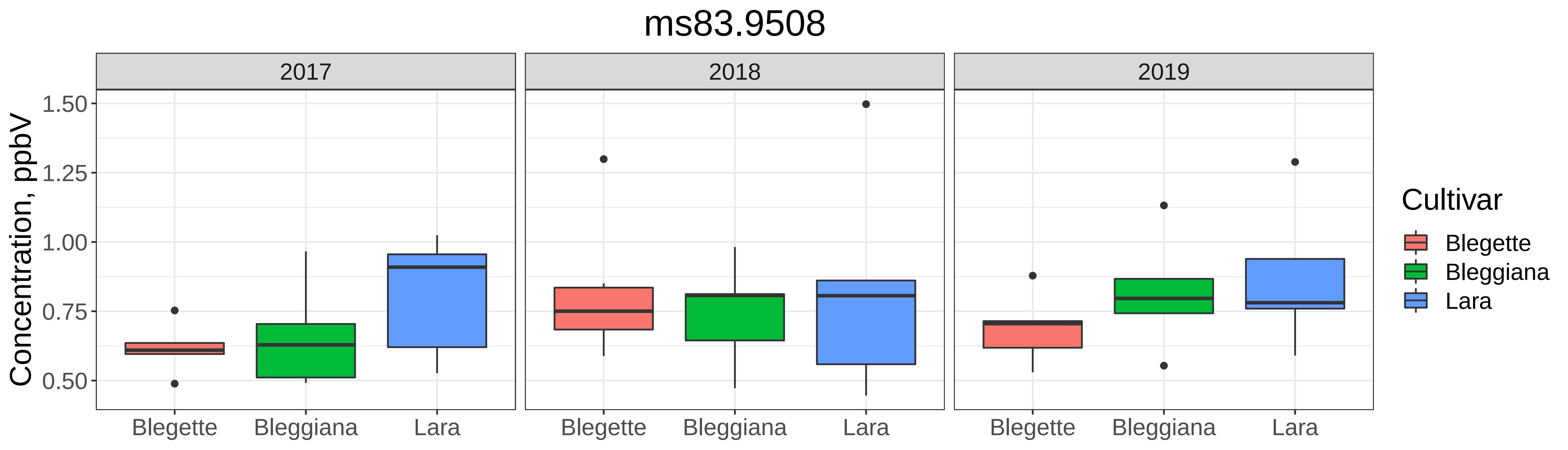

ms83.9508
2017
2018
2019
Concentration, ppbV
1.50
1.25
1.00
0.75
0.50
Blegette
Bleggiana
Lara
Cultivar
Blegette
Bleggiana
Lara

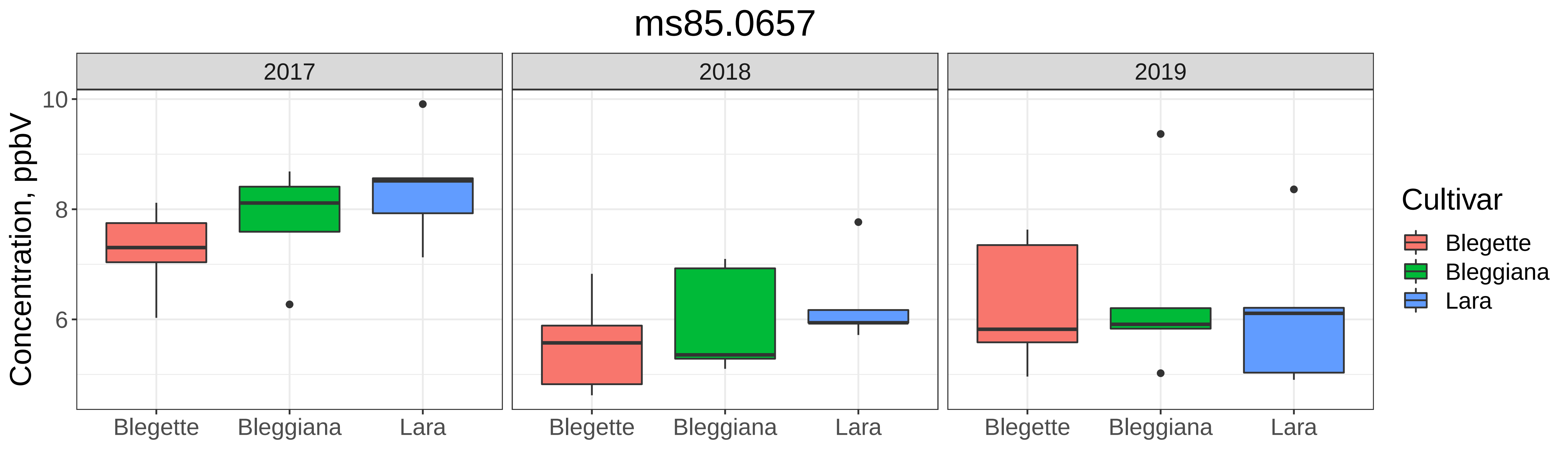

ms85.0657
2017
2018
2019
Concentration, ppbV
10
8
6
Blegette
Bleggiana
Lara
Cultivar
Blegette
Bleggiana
Lara

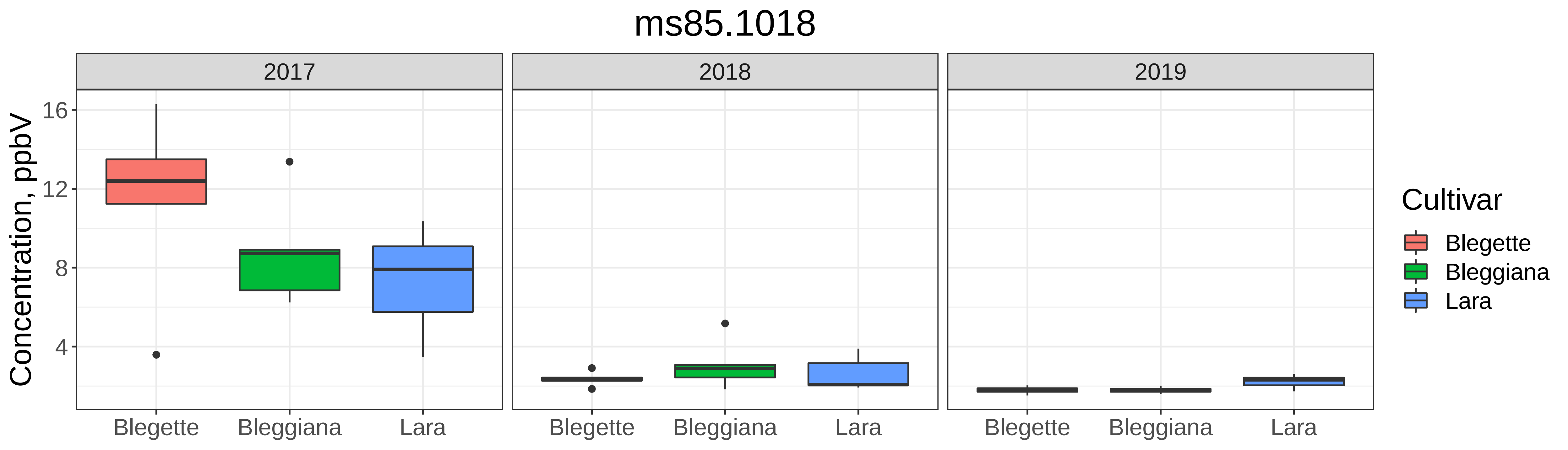

ms85.1018
2017
2018
2019
Concentration, ppbV
16
12
8
4
Blegette
Bleggiana
Lara
Cultivar
Blegette
Bleggiana
Lara

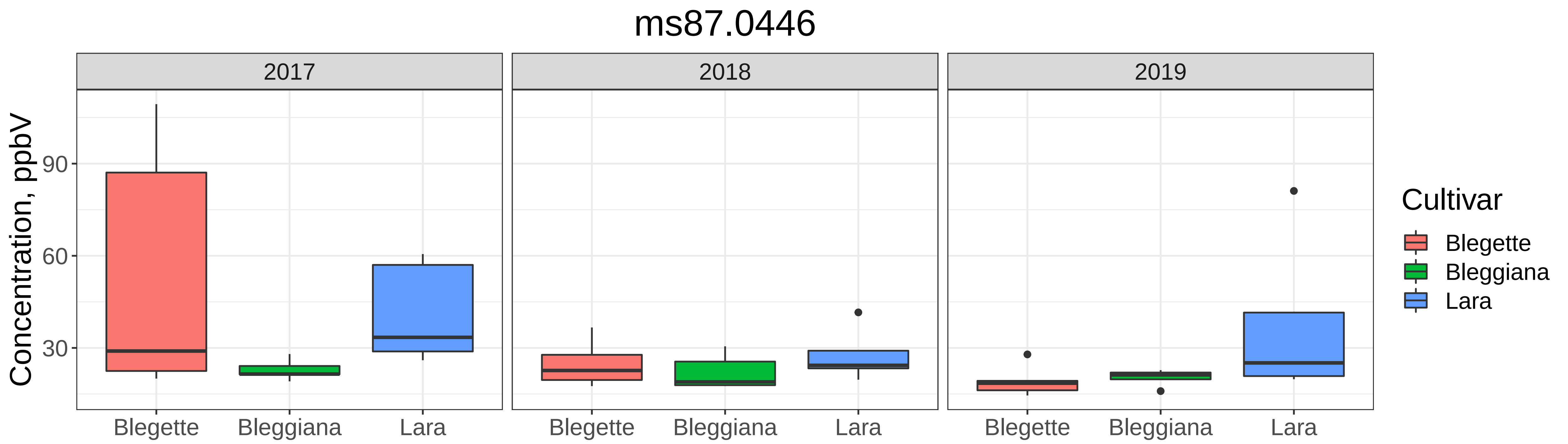
ms87.0446
2017
2018
2019
Concentration, ppbV
90
60
30
Blegette
Bleggiana
Lara
Cultivar
Blegette
Bleggiana
Lara

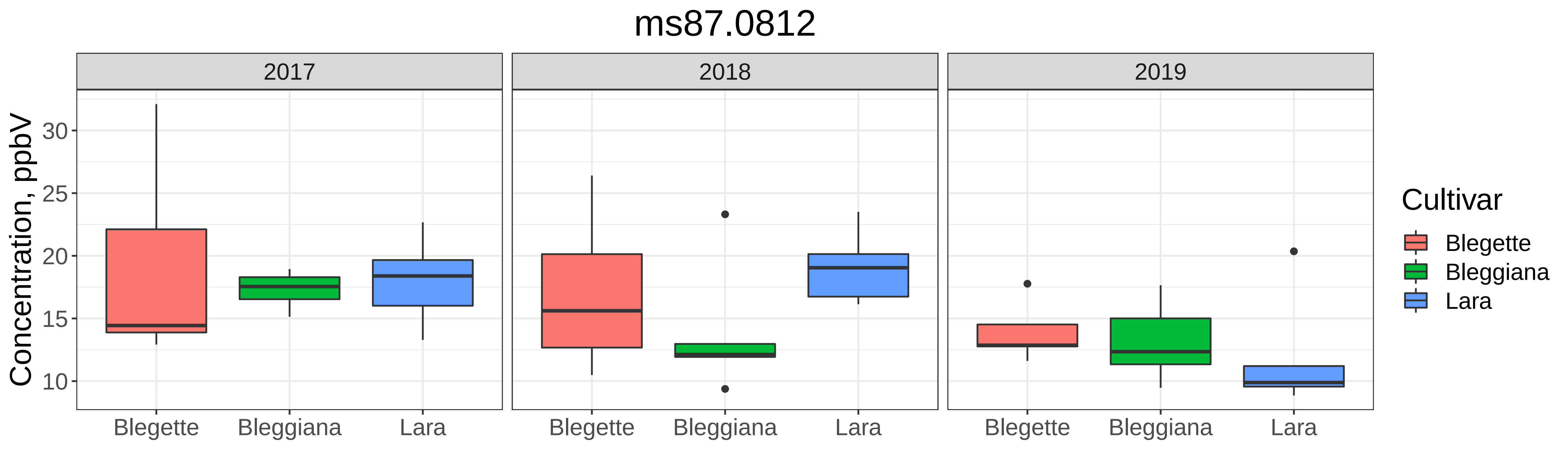
ms87.0812
2017
2018
2019
Concentration, ppbV
30
25
20
15
10
Blegette
Bleggiana
Lara
Cultivar
Blegette
Bleggiana
Lara

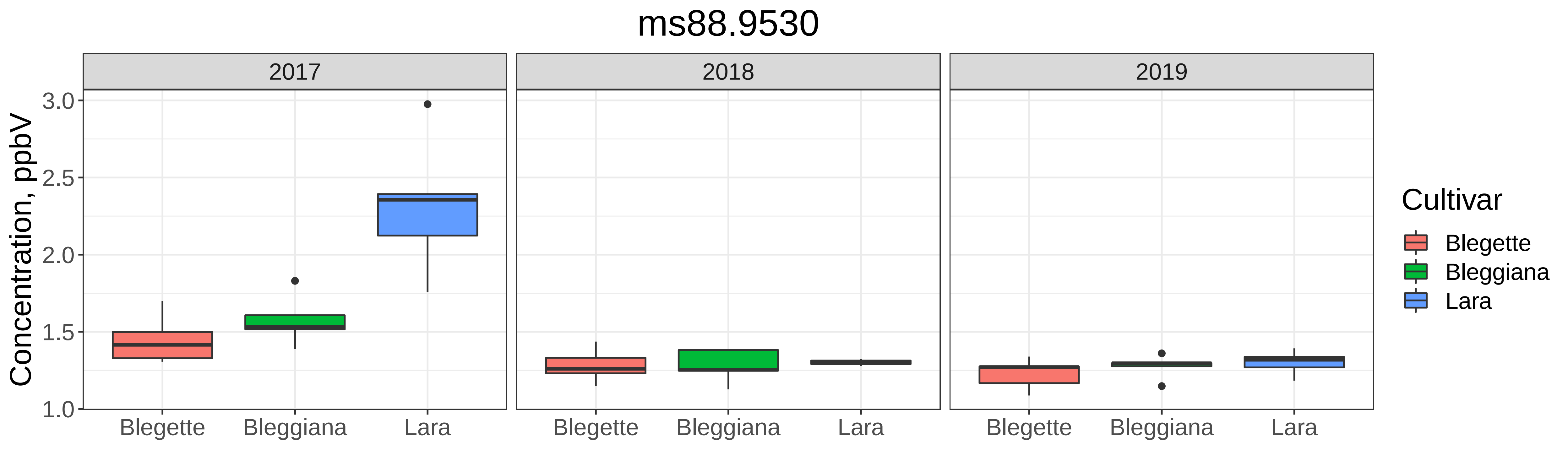

ms88.9530
2017
2018
2019
Concentration, ppbV
3.0
2.5
2.0
1.5
1.0
Blegette
Bleggiana
Lara
Blegette
Bleggiana
Lara
Blegette
Bleggiana
Lara
Cultivar
Blegette
Bleggiana
Lara

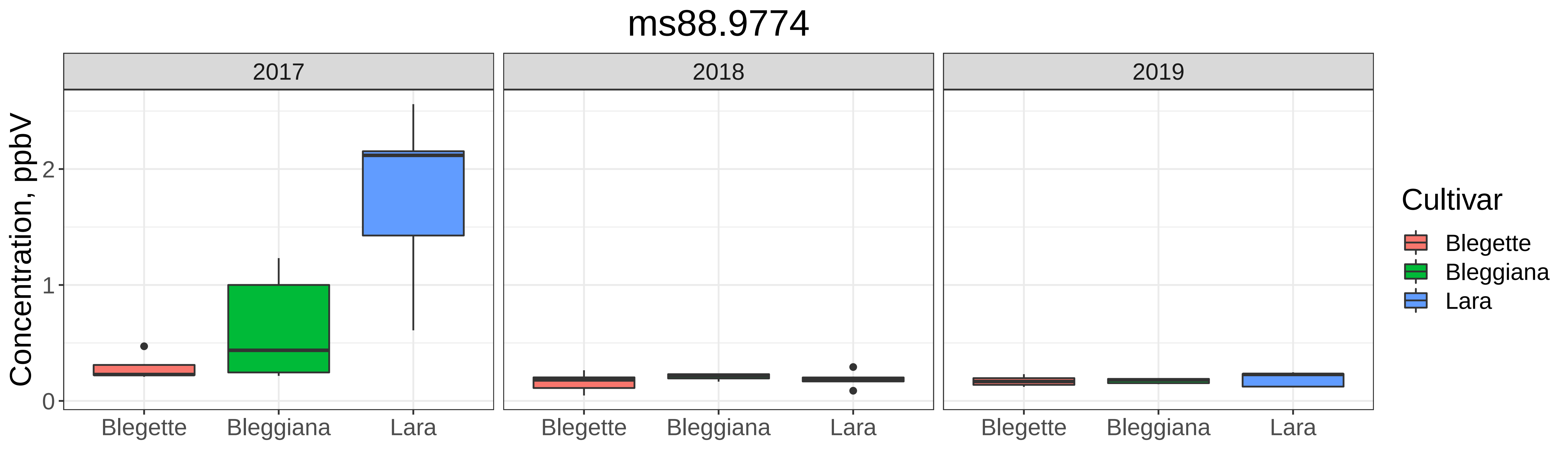
ms88.9774
2017
2018
2019
Concentration, ppbV
0
1
2
Blegette
Bleggiana
Lara
Cultivar
Blegette
Bleggiana
Lara

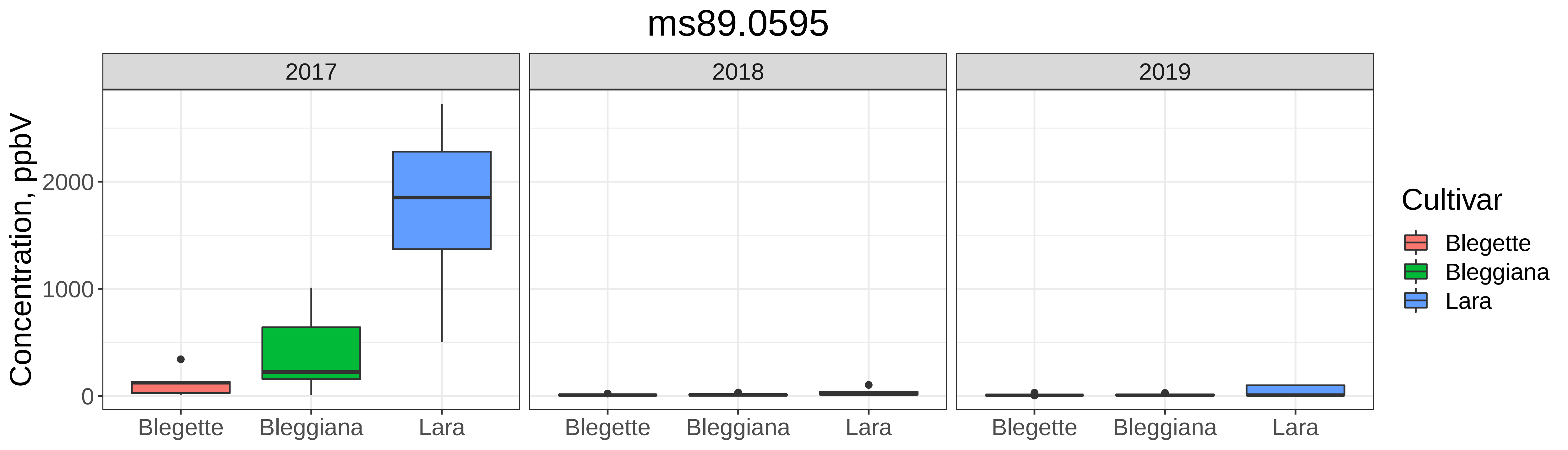

ms89.0595
2017
2018
2019
Concentration, ppbV
0
1000
2000
Blegette
Bleggiana
Lara
Cultivar
Blegette
Bleggiana
Lara

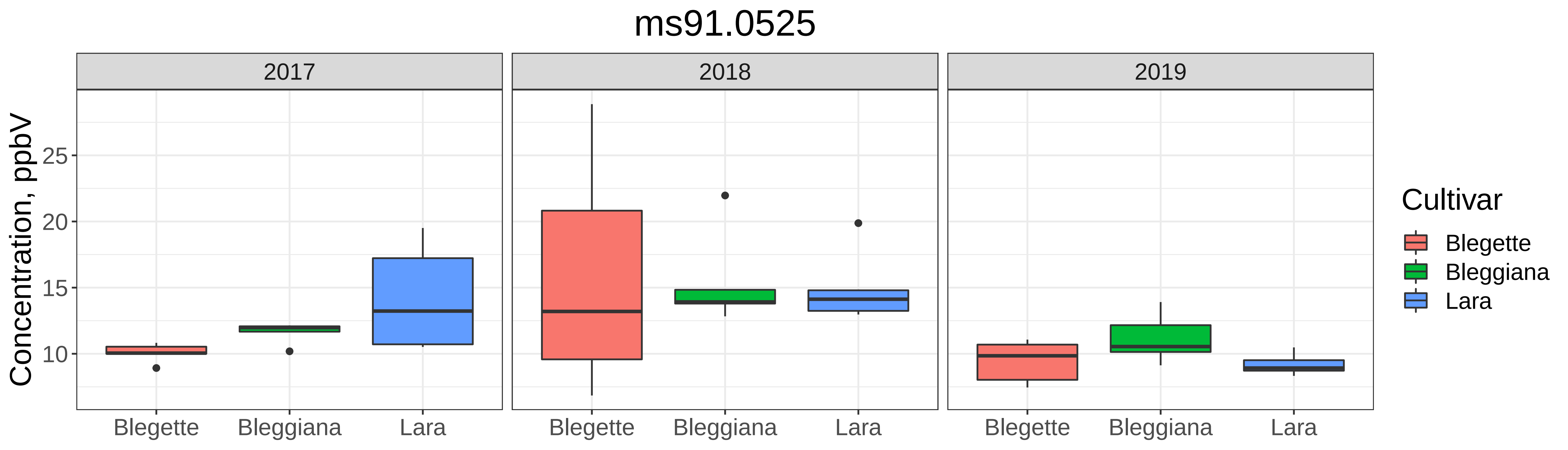
ms91.0525
2017
2018
2019
Concentration, ppbV
25
20
15
10
Blegette
Bleggiana
Lara
Cultivar
Blegette
Bleggiana
Lara

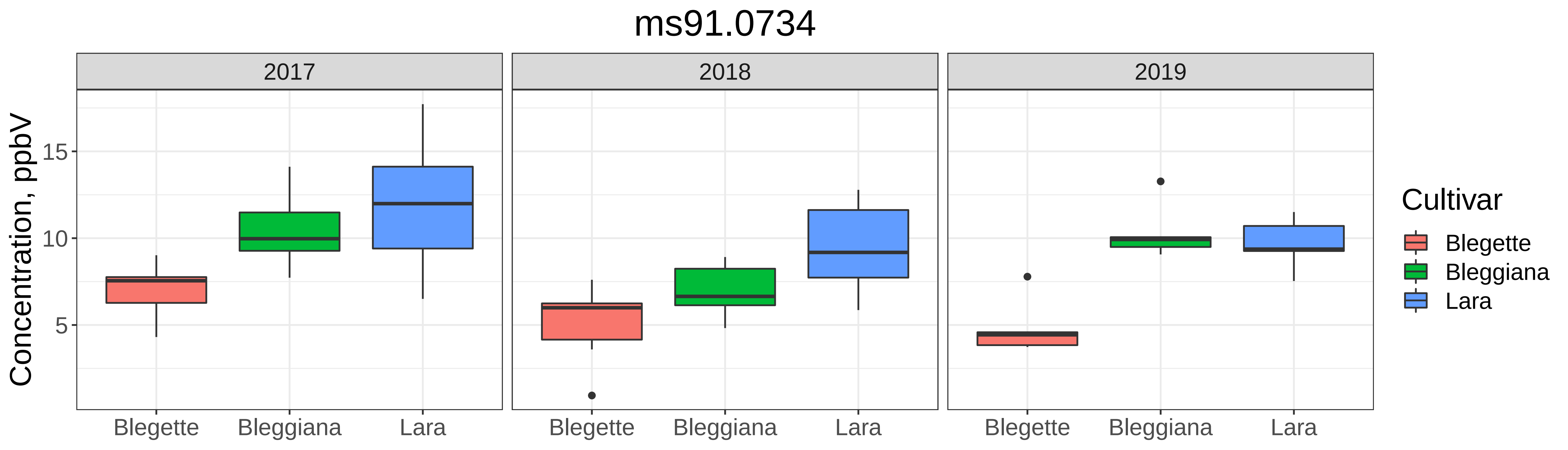

ms91.0734
2017
2018
2019
Concentration, ppbV
15
10
5
Blegette
Bleggiana
Lara
Cultivar
Blegette
Bleggiana
Lara

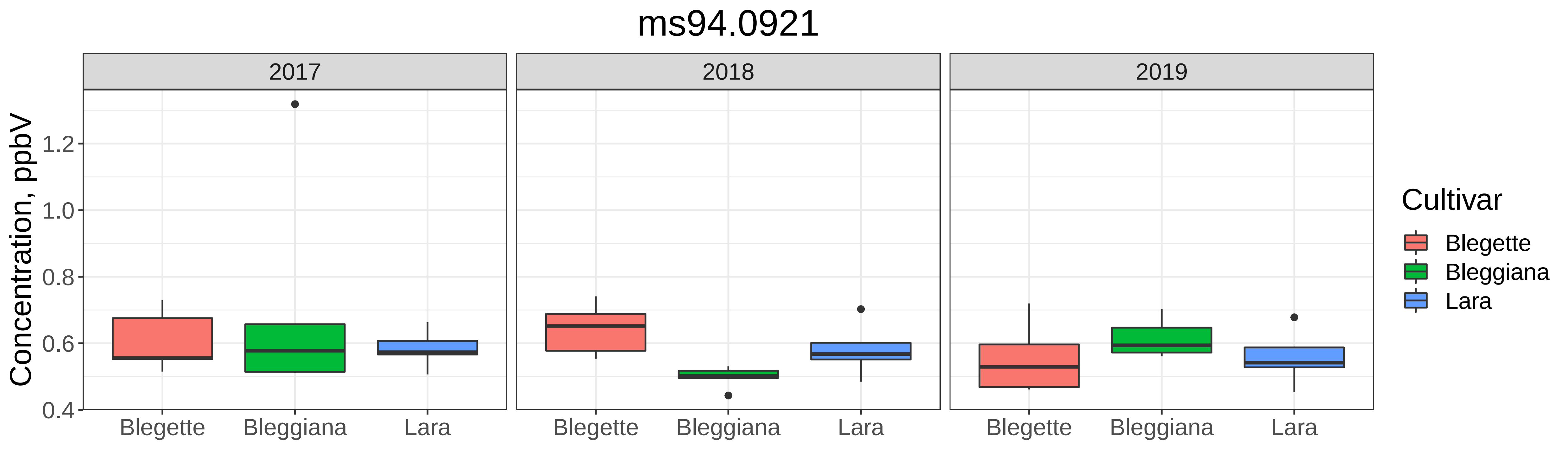

ms94.0921
2017
2018
2019
Concentration, ppbV
1.2
1.0
0.8
0.6
0.4
Blegette
Bleggiana
Lara
Cultivar
Blegette
Bleggiana
Lara

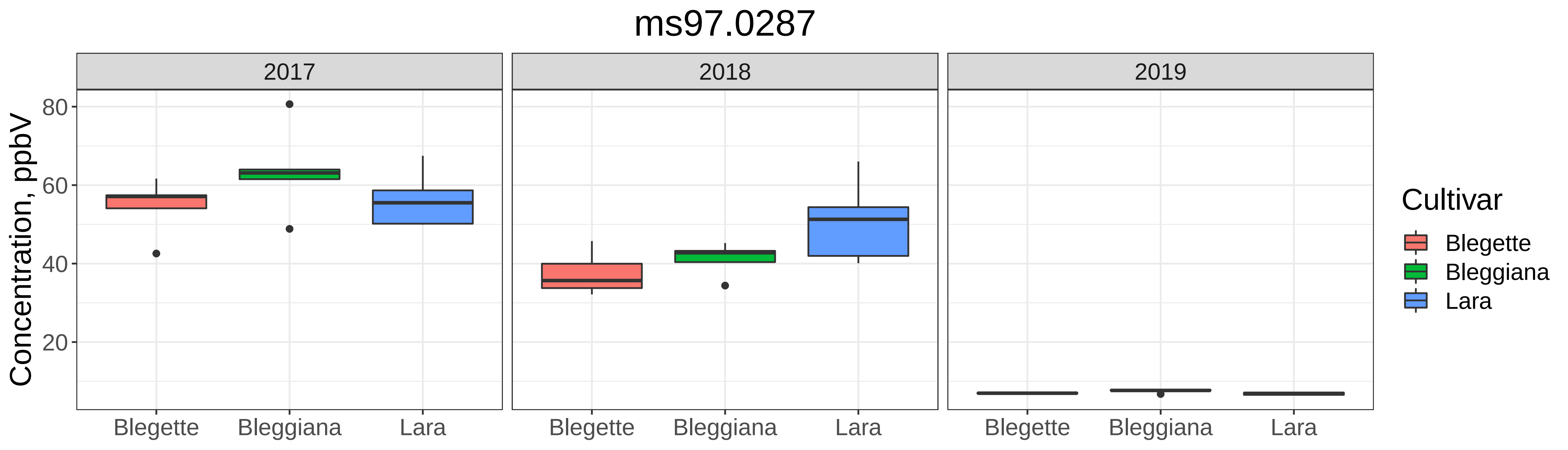
ms97.0287
2017
2018
2019
Concentration, ppbV
80
60
40
20
Blegette
Bleggiana
Lara
Cultivar
Blegette
Bleggiana
Lara

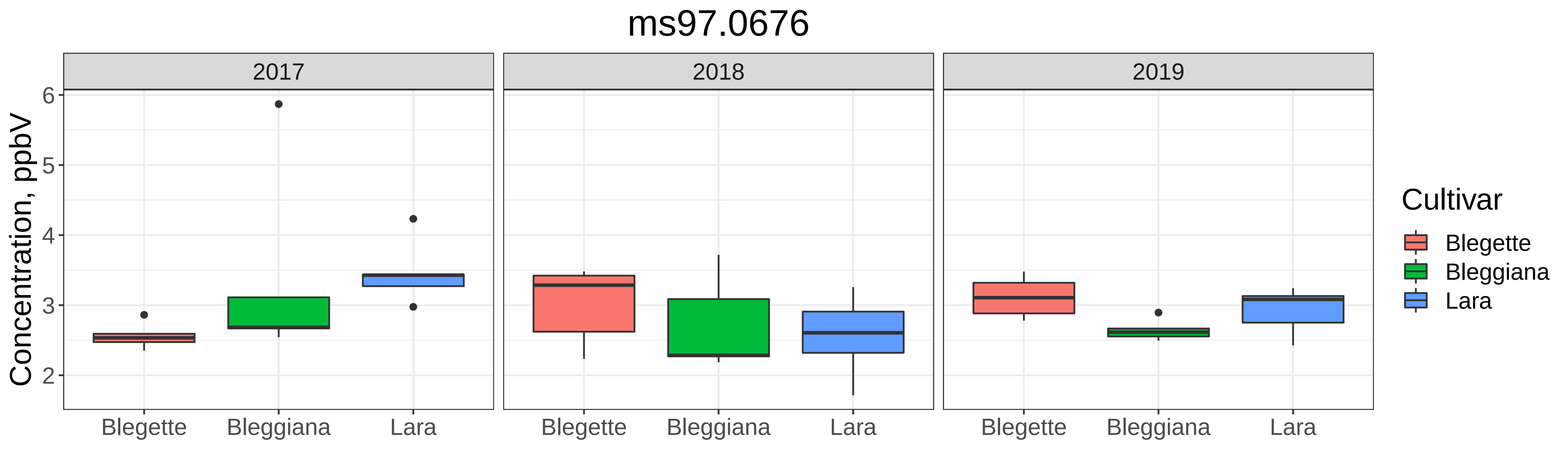

ms97.0676
2017
2018
2019
Concentration, ppbV
6
5
4
3
2
Blegette
Bleggiana
Lara
Cultivar
Blegette
Bleggiana
Lara

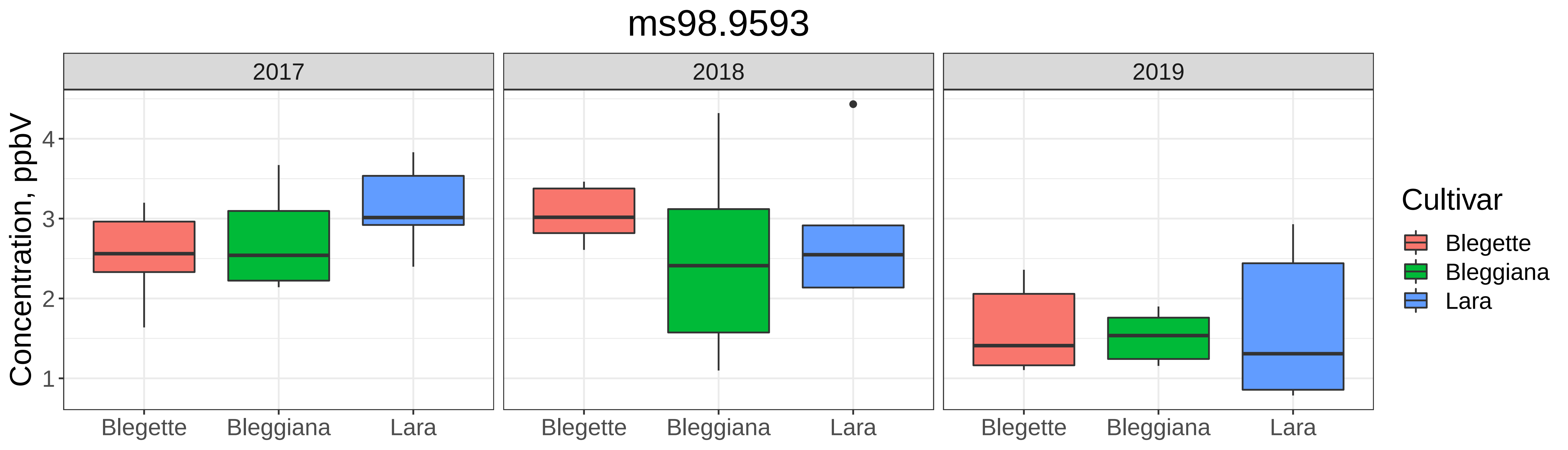

ms98.9593
2017
2018
2019
Concentration, ppbV
4
3
2
1
Blegette
Bleggiana
Lara
Blegette
Bleggiana
Lara
Blegette
Bleggiana
Lara
Cultivar
Blegette
Bleggiana
Lara

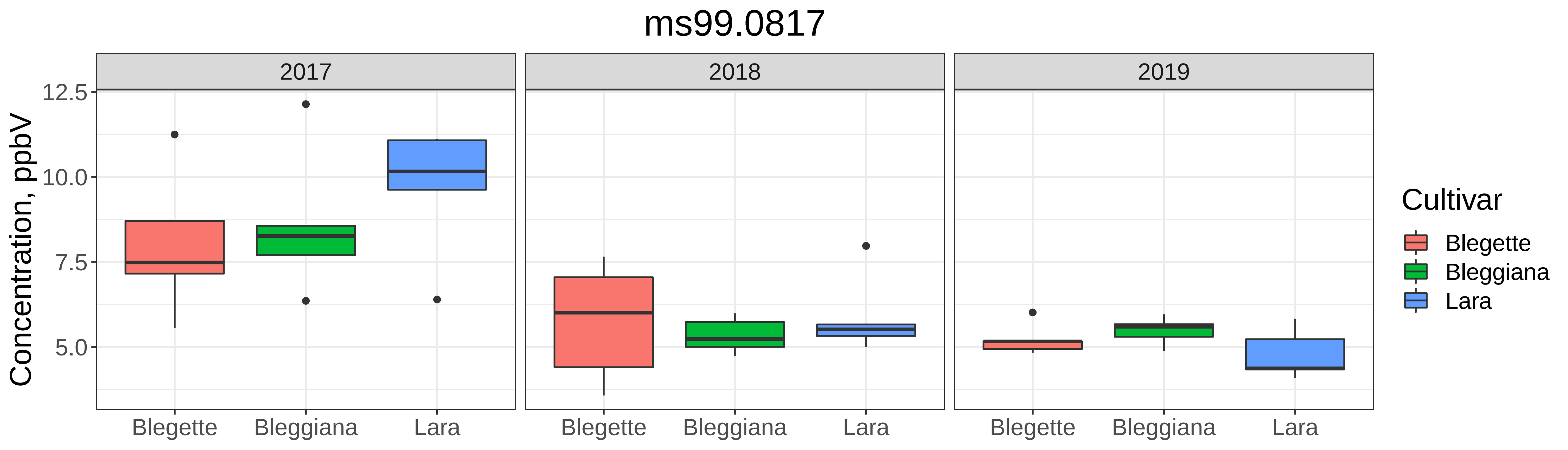
ms99.0817
2017
2018
2019
Concentration, ppbV
12.5
10.0
7.5
5.0
Blegette
Bleggiana
Lara
Cultivar
Blegette
Bleggiana
Lara

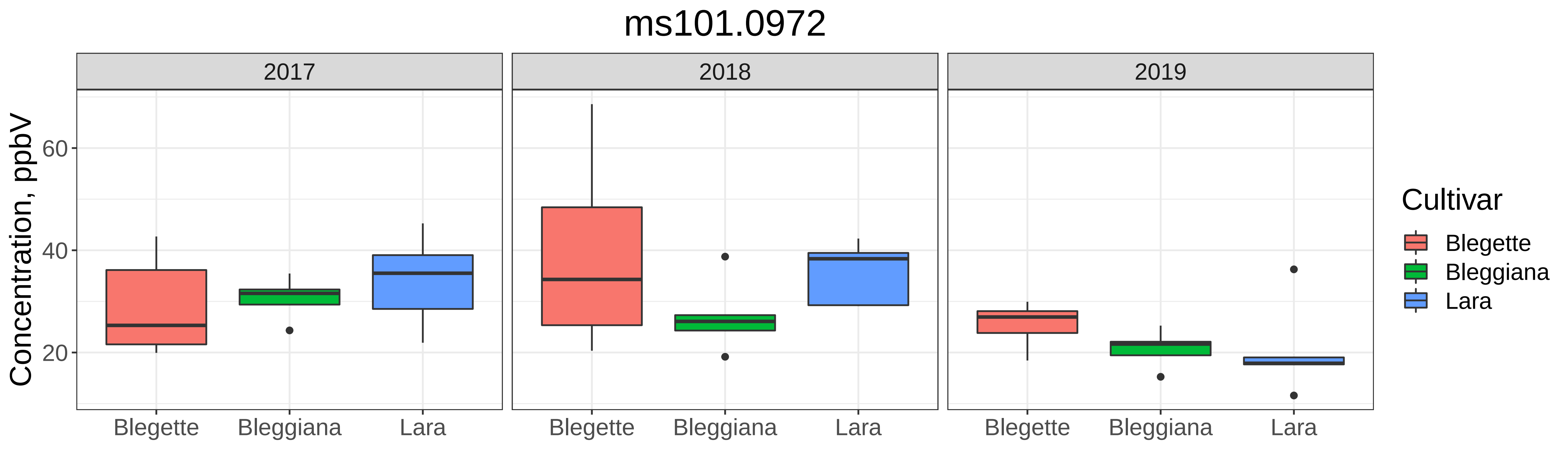
ms101.0972
2017
2018
2019
Concentration, ppbV
60
40
20
Blegette
Bleggiana
Lara
Cultivar
Blegette
Bleggiana
Lara

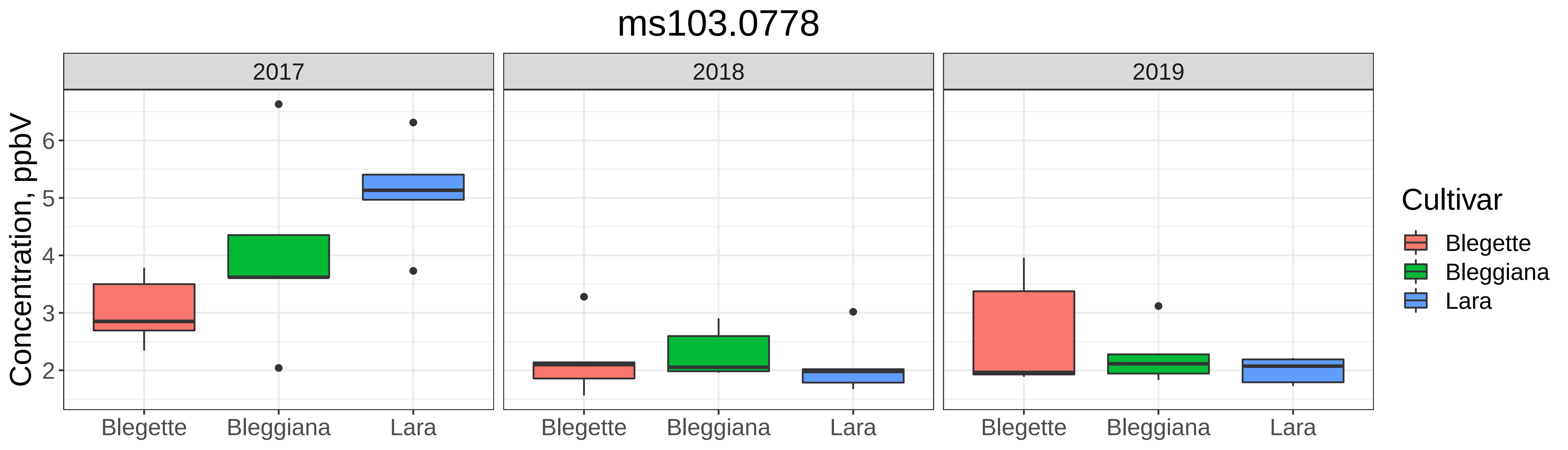

ms103.0778
2017
2018
2019
Concentration, ppbV
6
5
4
3
2
Blegette
Bleggiana
Lara
Cultivar
Blegette
Bleggiana
Lara

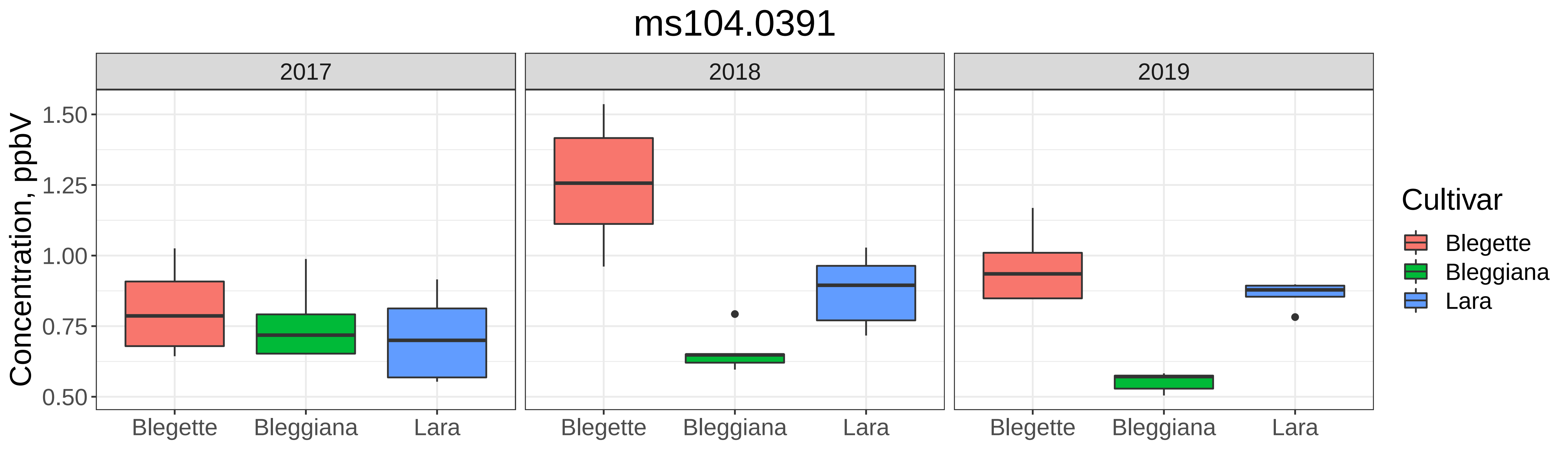
ms104.0391
2017
2018
2019
Concentration, ppbV
1.50
1.25
1.00
0.75
0.50
Blegette
Bleggiana
Lara
Cultivar
Blegette
Bleggiana
Lara

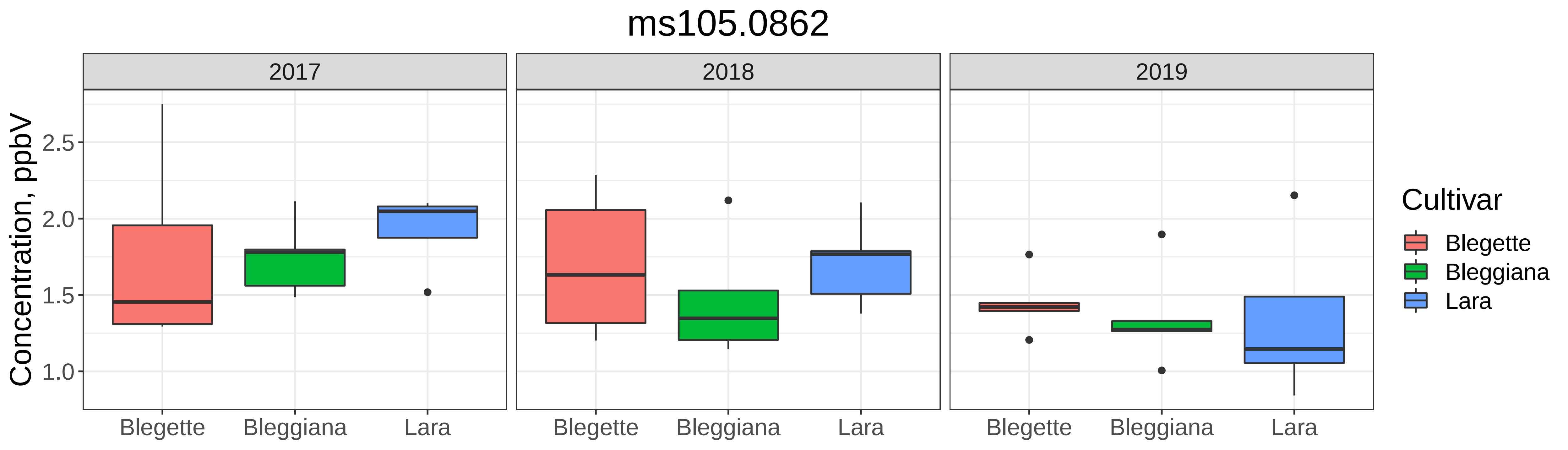

ms105.0862
2017
2018
2019
Concentration, ppbV
2.5
2.0
1.5
1.0
Blegette
Bleggiana
Lara
Cultivar
Blegette
Bleggiana
Lara

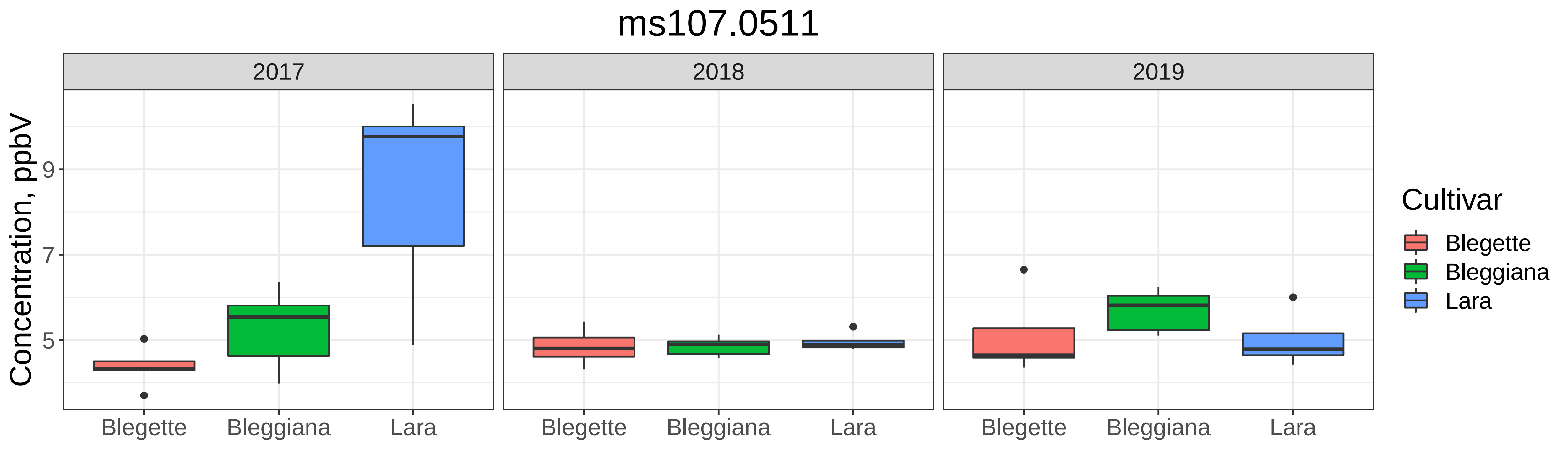
ms107.0511
2017
2018
2019
Concentration, ppbV
9
7
5
Blegette
Bleggiana
Lara
Cultivar
Blegette
Bleggiana
Lara

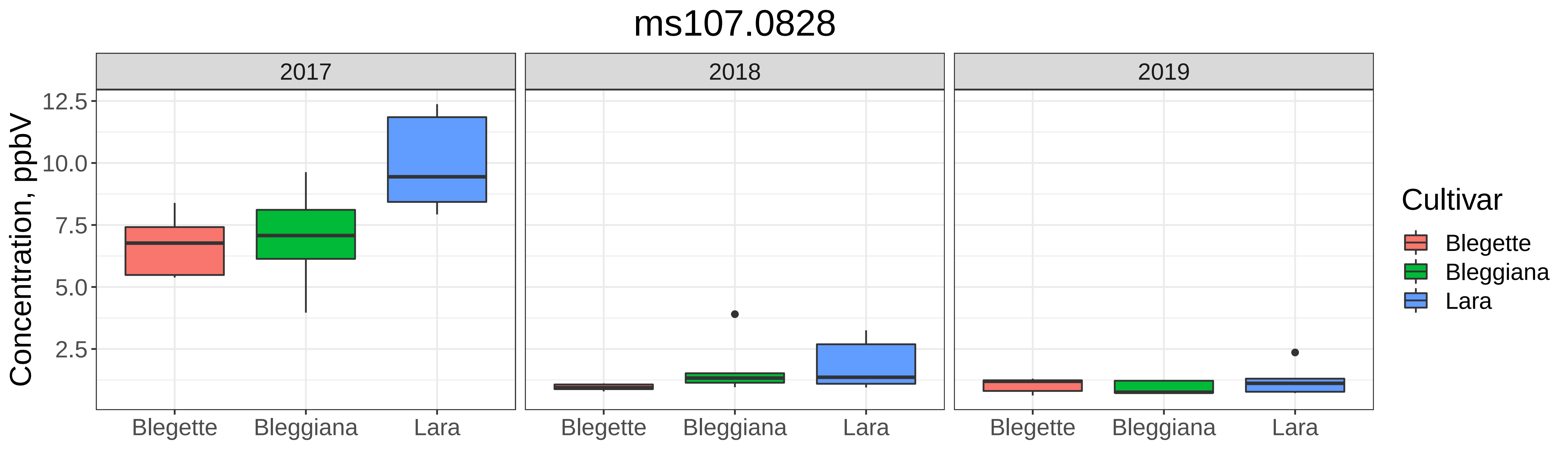

ms107.0828
2017
2018
2019
Concentration, ppbV
12.5
10.0
7.5
5.0
2.5
Blegette
Bleggiana
Lara
Cultivar
Blegette
Bleggiana
Lara

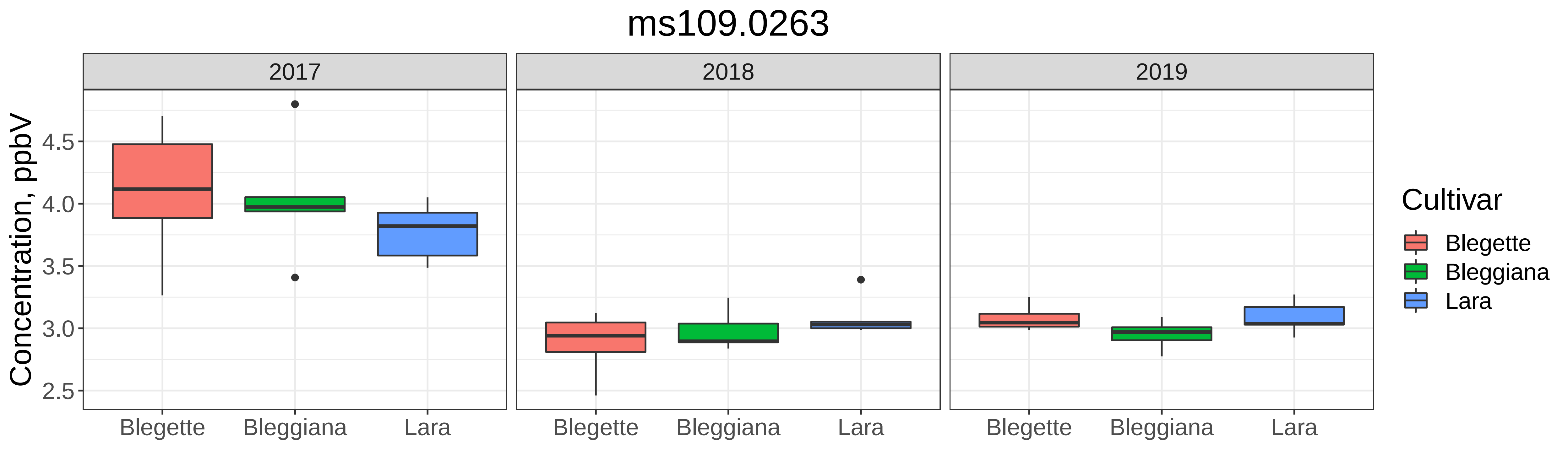
ms109.0263
2017
2018
2019
Concentration, ppbV
4.5
4.0
3.5
3.0
2.5
Blegette
Bleggiana
Lara
Cultivar
Blegette
Bleggiana
Lara

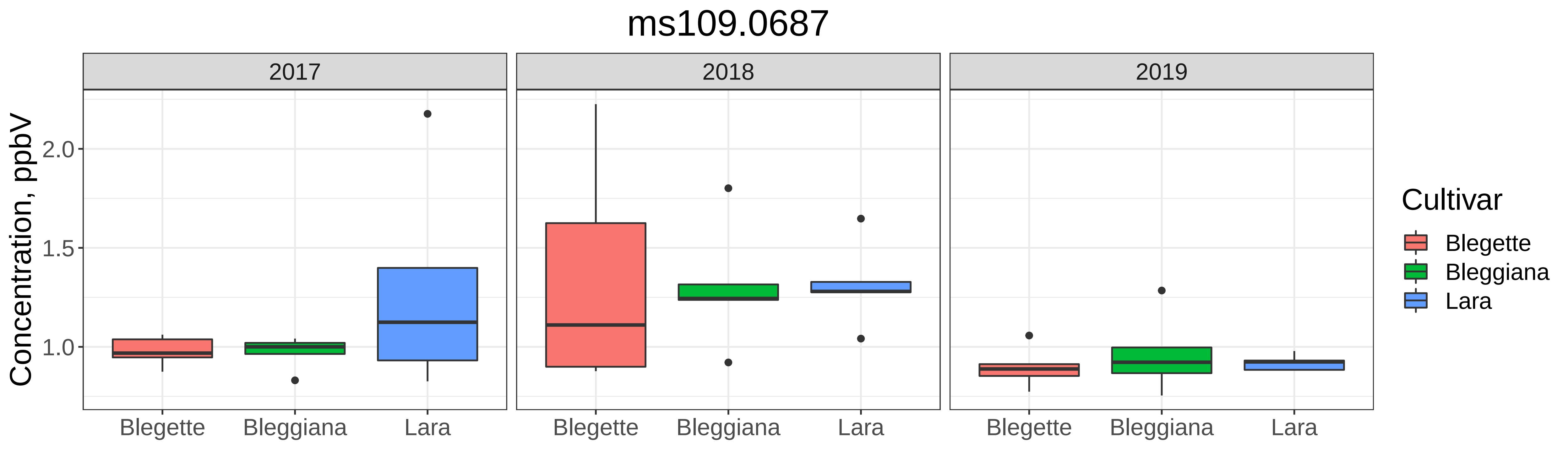

ms109.0687
2017
2018
2019
Concentration, ppbV
2.0
1.5
1.0
Blegette
Bleggiana
Lara
Cultivar
Blegette
Bleggiana
Lara

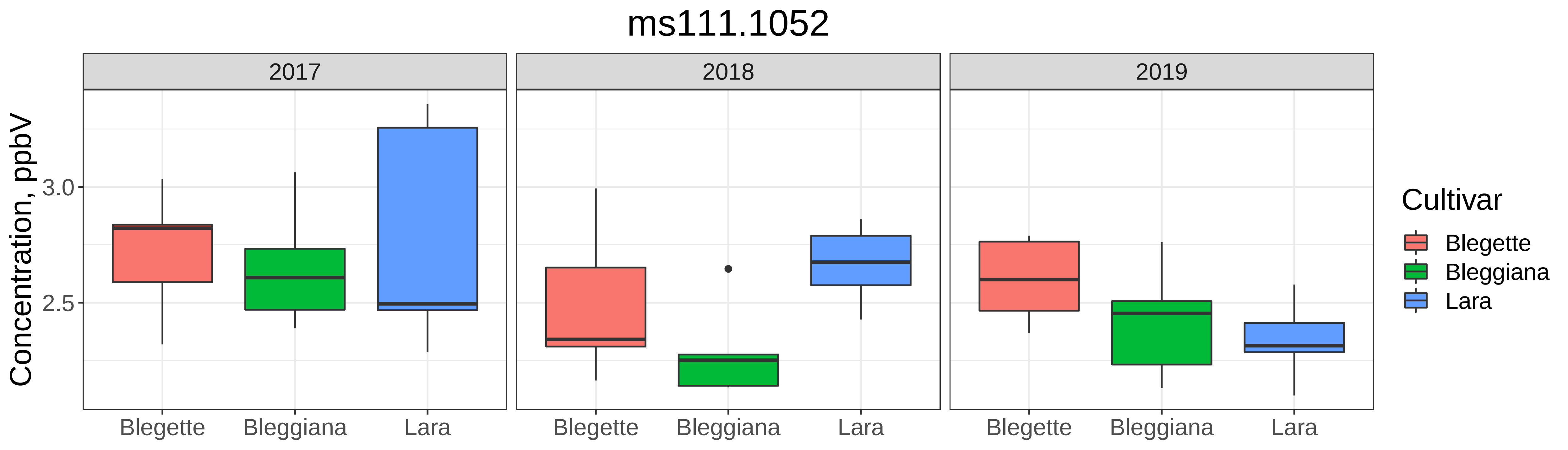

ms111.1052
2017
2018
2019
Concentration, ppbV
3.0
2.5
Blegette
Bleggiana
Lara
Cultivar
Blegette
Bleggiana
Lara

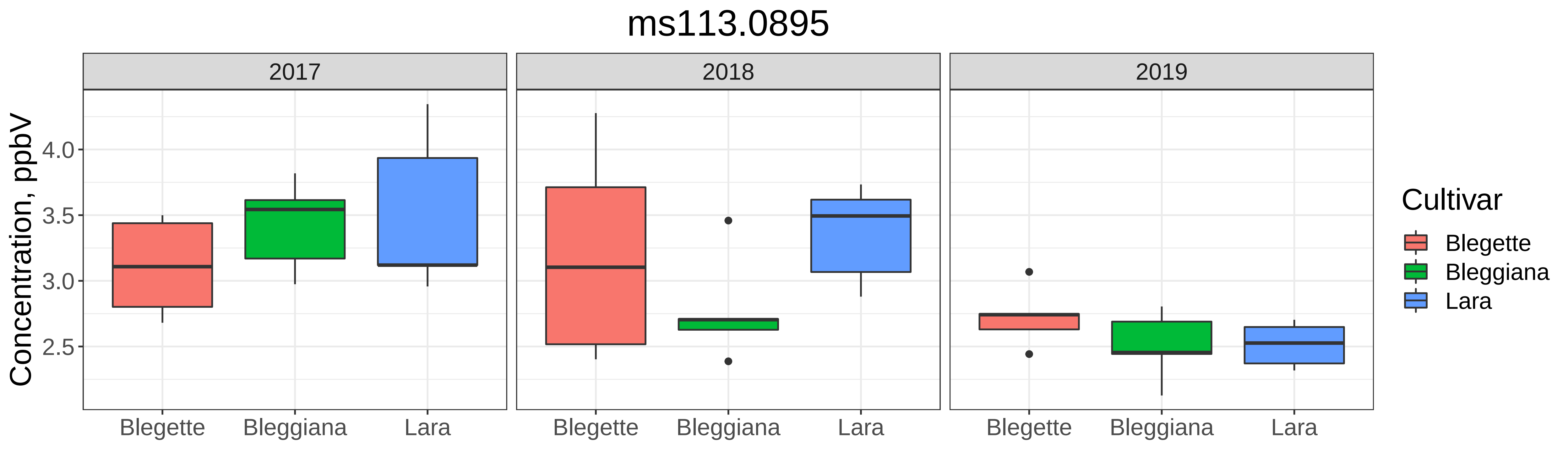

ms113.0895
2017
2018
2019
Concentration, ppbV
4.0
3.5
3.0
2.5
Blegette
Bleggiana
Lara
Cultivar
Blegette
Bleggiana
Lara

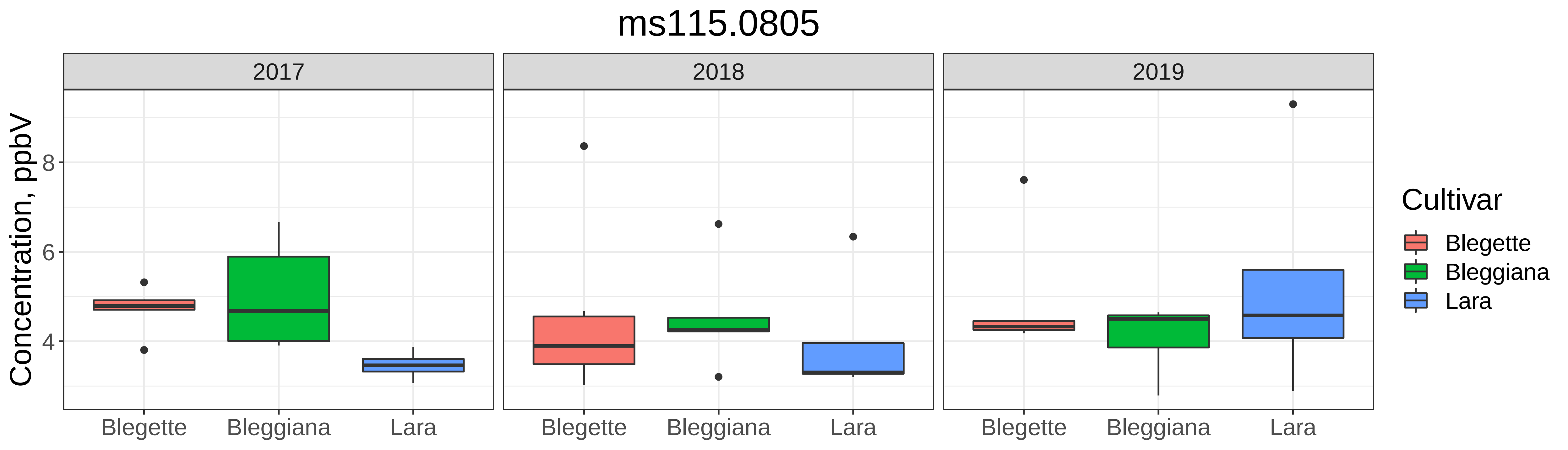
ms115.0805
2017
2018
2019
Concentration, ppbV
8
6
4
Blegette
Bleggiana
Lara
Cultivar
Blegette
Bleggiana
Lara

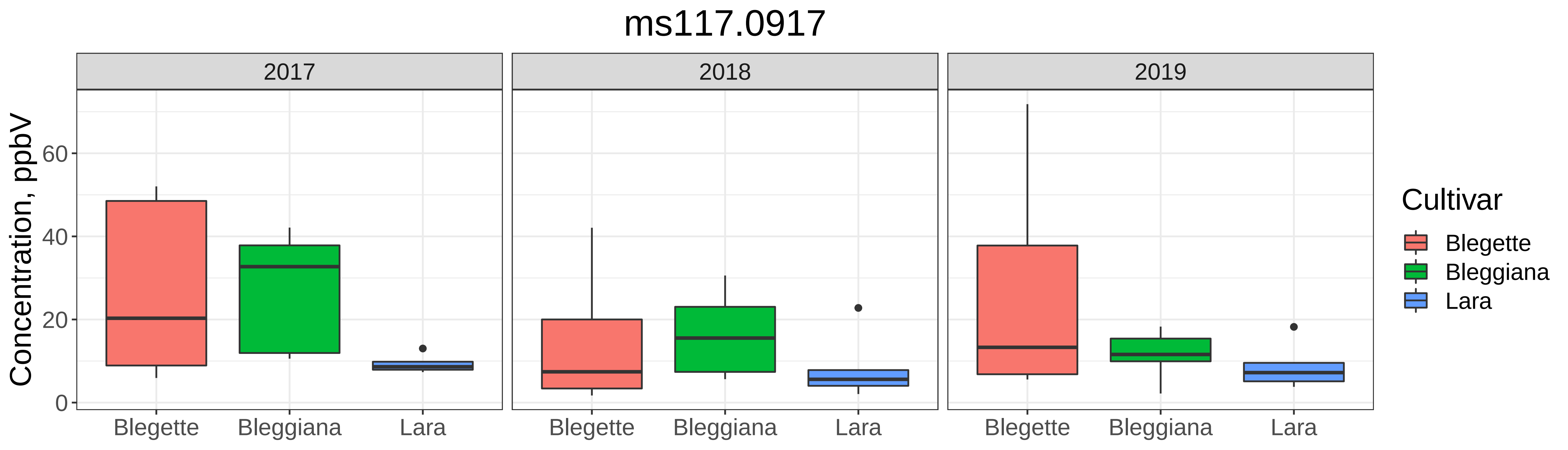

ms117.0917
2017
2018
2019
Concentration, ppbV
0
20
40
60
Blegette
Bleggiana
Lara
Cultivar
Blegette
Bleggiana
Lara

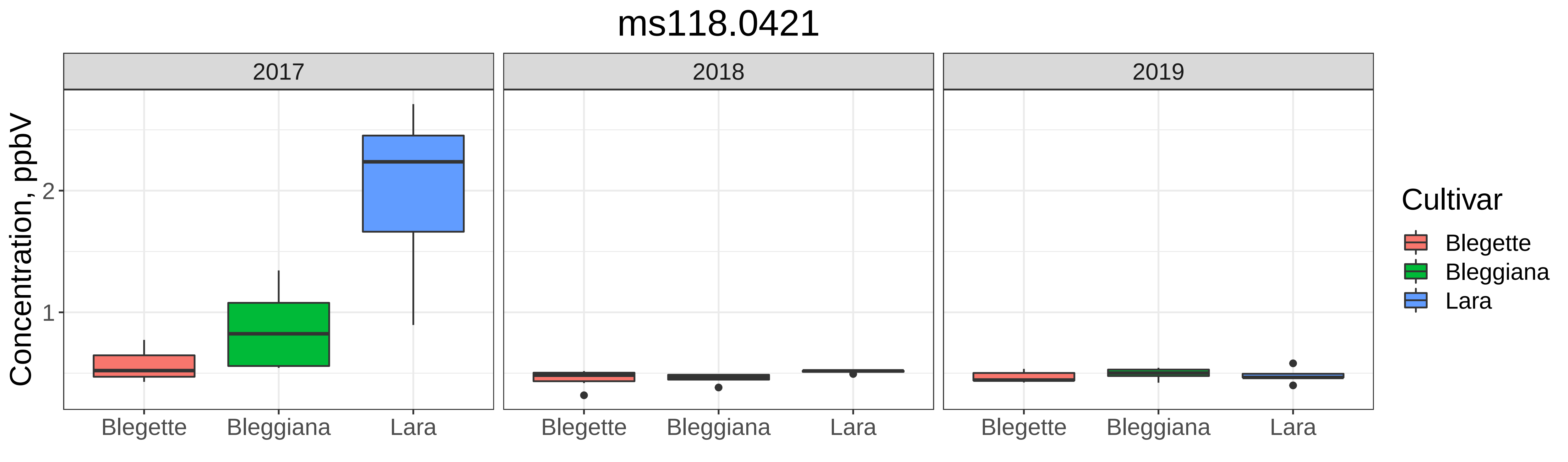

ms118.0421
2017
2018
2019
Concentration, ppbV
2
1
Blegette
Bleggiana
Lara
Cultivar
Blegette
Bleggiana
Lara

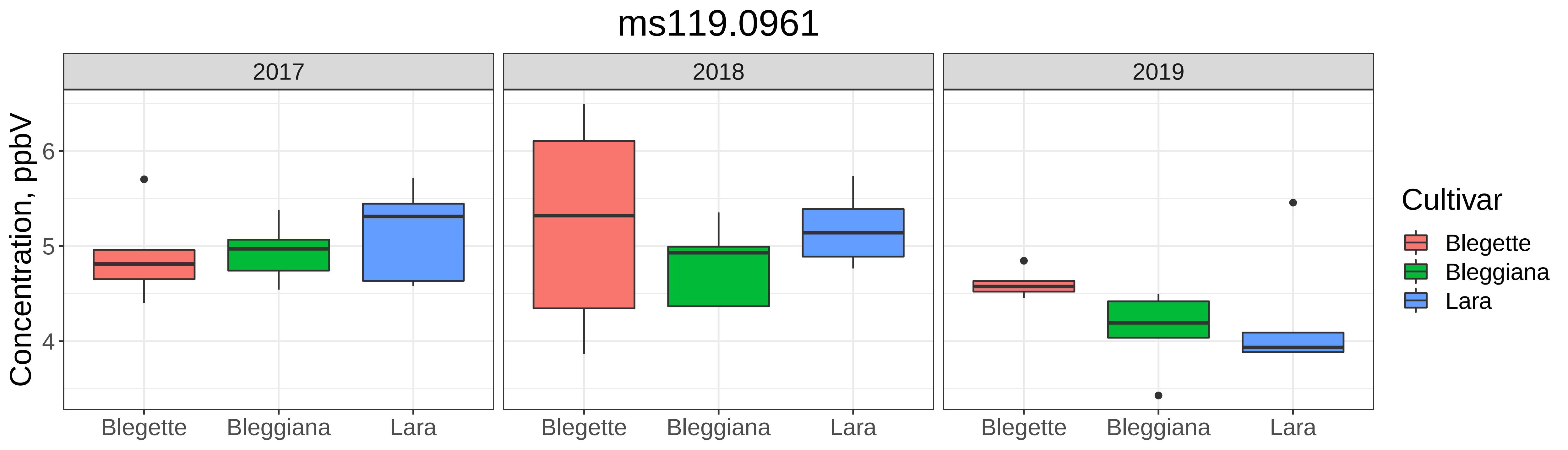

ms119.0961
2017
2018
2019
Concentration, ppbV
6
5
4
Blegette
Bleggiana
Lara
Cultivar
Blegette
Bleggiana
Lara

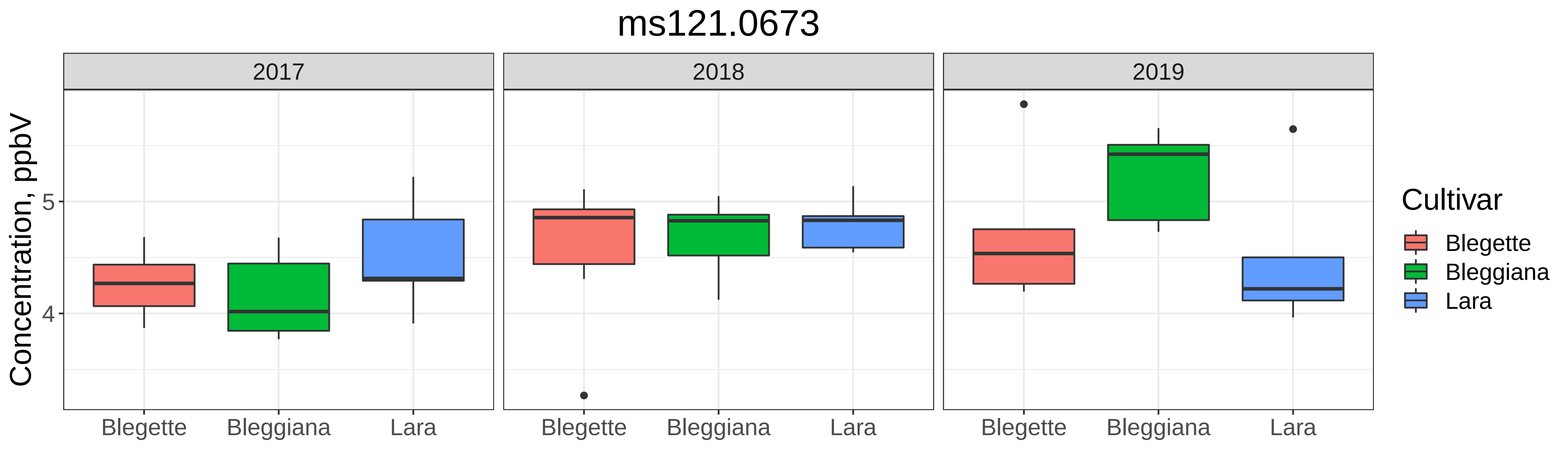

ms121.0673
2017
2018
2019
Concentration, ppbV
5
4
Blegette
Bleggiana
Lara
Cultivar
Blegette
Bleggiana
Lara

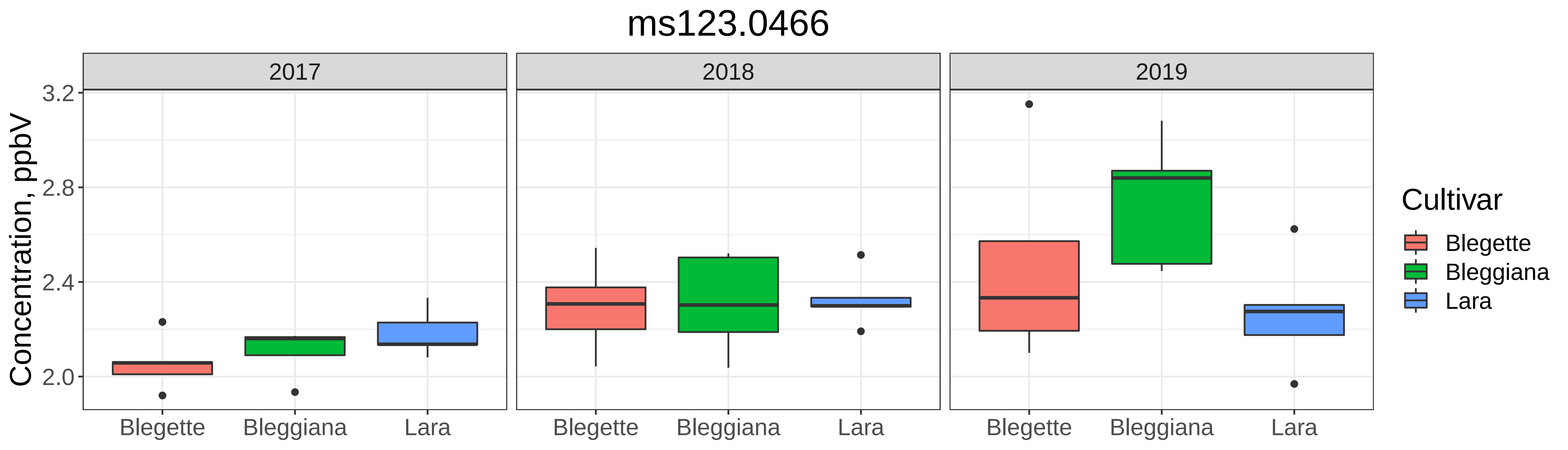

ms123.0466
2017
2018
2019
Concentration, ppbV
3.2
2.8
2.4
2.0
Blegette
Bleggiana
Lara
Cultivar
Blegette
Bleggiana
Lara

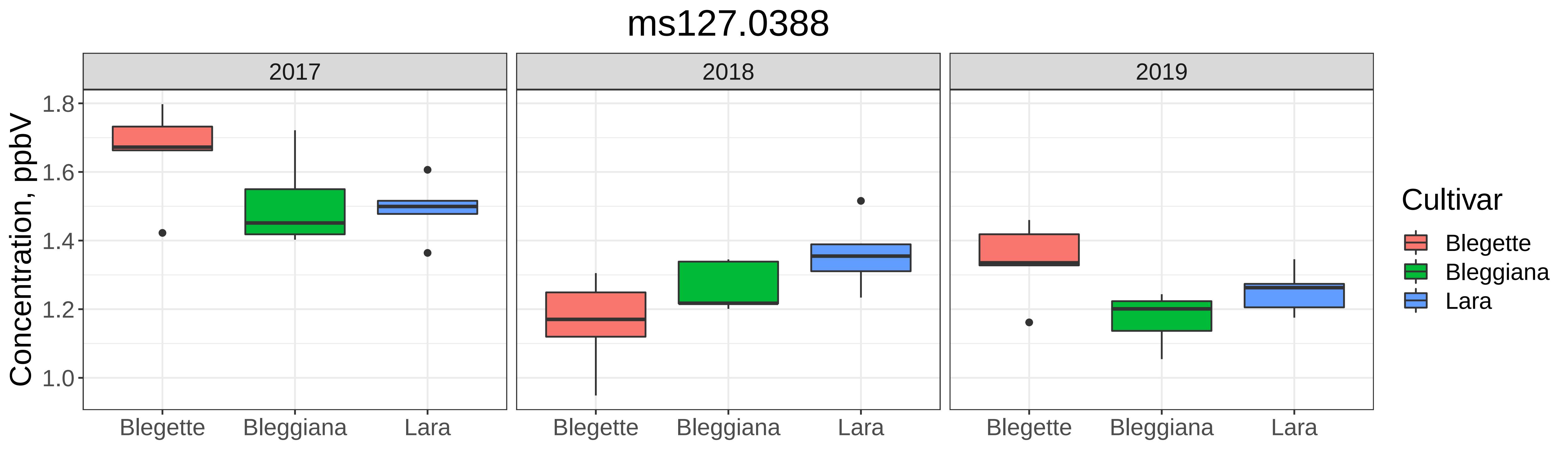

ms127.0388
2017
2018
2019
Concentration, ppbV
1.0
1.2
1.4
1.6
1.8
Blegette
Bleggiana
Lara
Cultivar
Blegette
Bleggiana
Lara

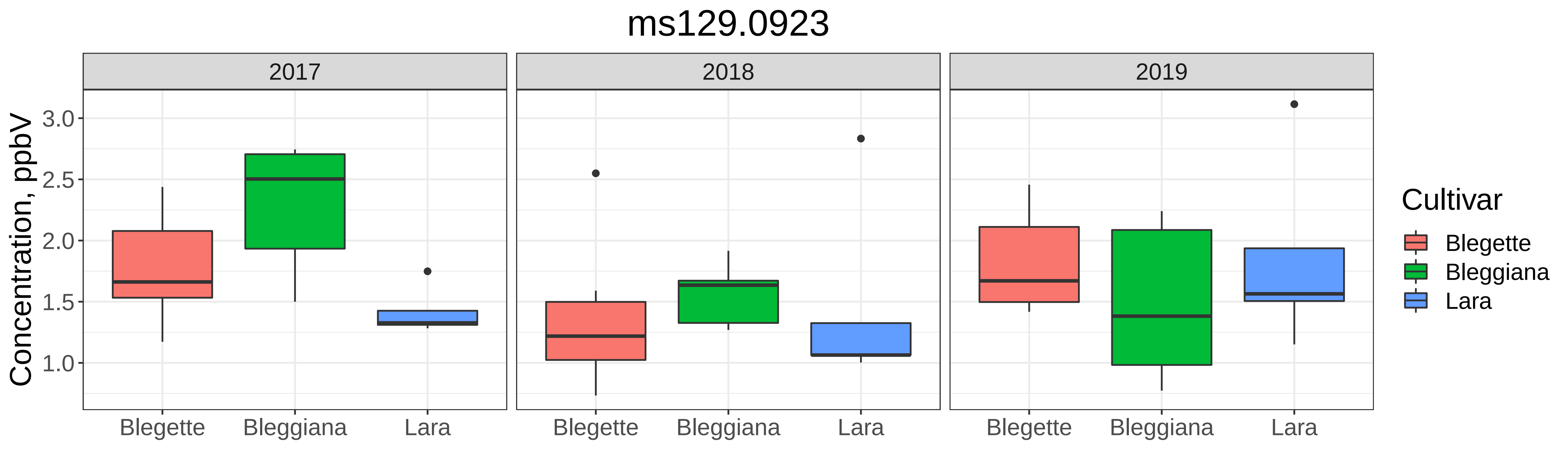

ms129.0923
2017
2018
2019
Concentration, ppbV
3.0
2.5
2.0
1.5
1.0
Blegette
Bleggiana
Lara
Cultivar
Blegette
Bleggiana
Lara

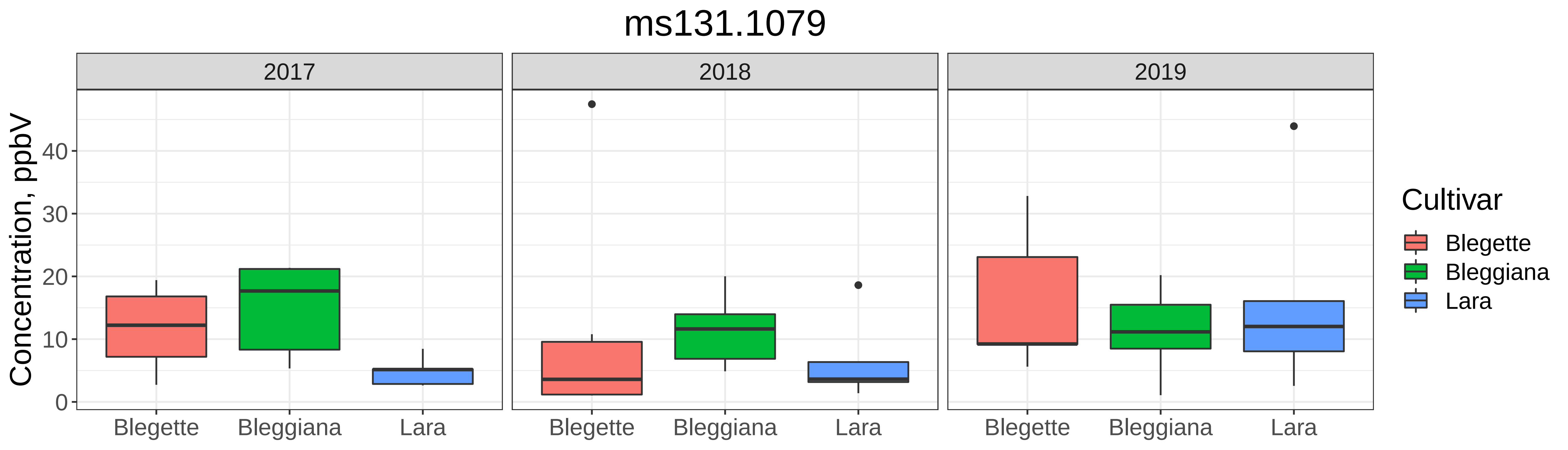

ms131.1079
2017
2018
2019
Concentration, ppbV
0
10
20
30
40
Blegette
Bleggiana
Lara
Cultivar
Blegette
Bleggiana
Lara

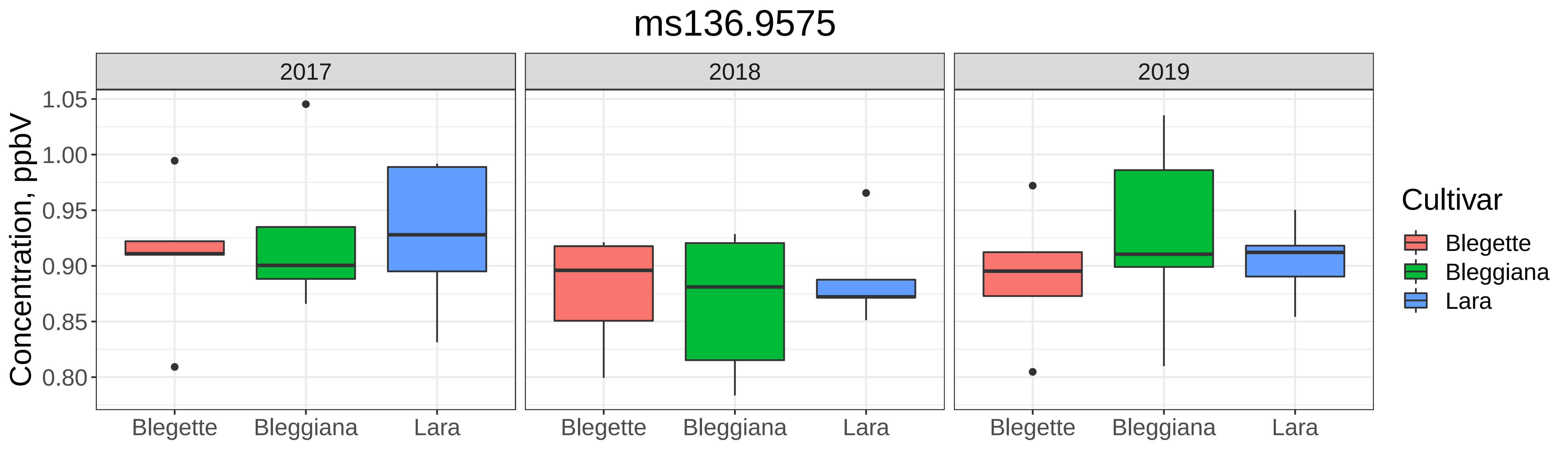

ms136.9575
2017
2018
2019
Concentration, ppbV
1.05
1.00
0.95
0.90
0.85
0.80
Blegette
Bleggiana
Lara
Cultivar
Blegette
Bleggiana
Lara

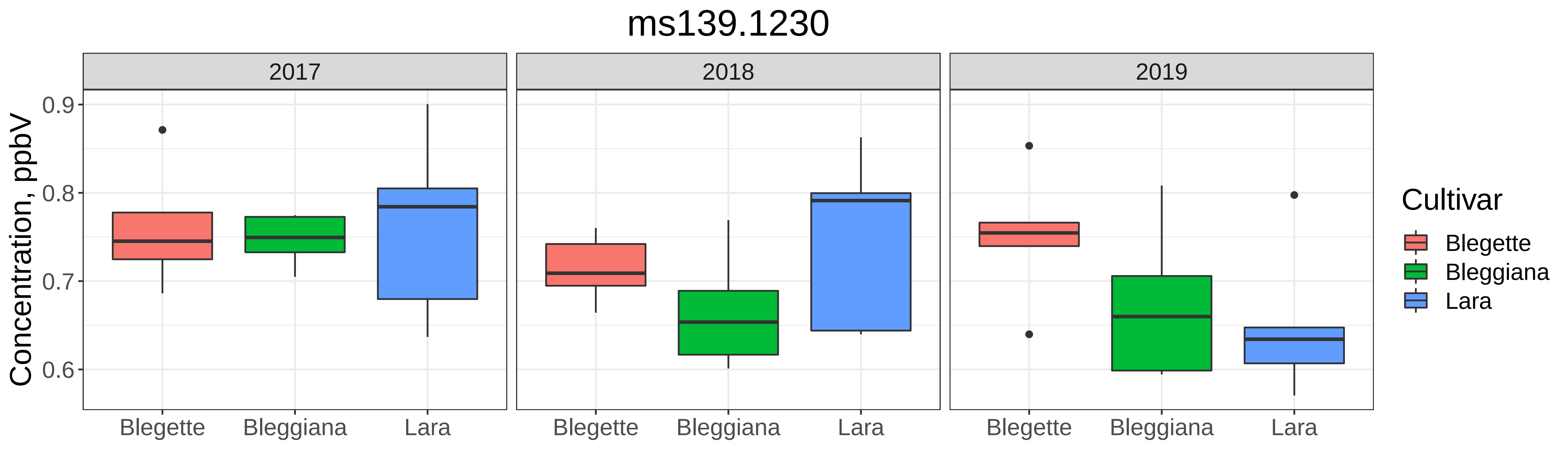
ms139.1230
2017
2018
2019
Concentration, ppbV
0.9
0.8
0.7
0.6
Blegette
Bleggiana
Lara
Cultivar
Blegette
Bleggiana
Lara

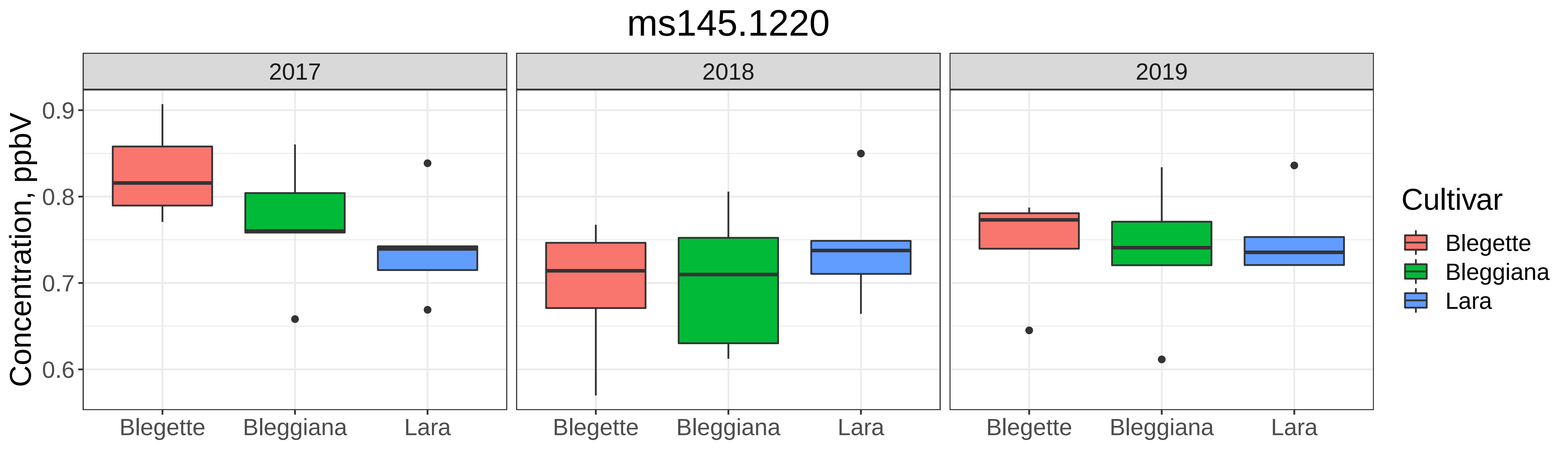

ms145.1220
2017
2018
2019
Concentration, ppbV
0.9
0.8
0.7
0.6
Blegette
Bleggiana
Lara
Cultivar
Blegette
Bleggiana
Lara

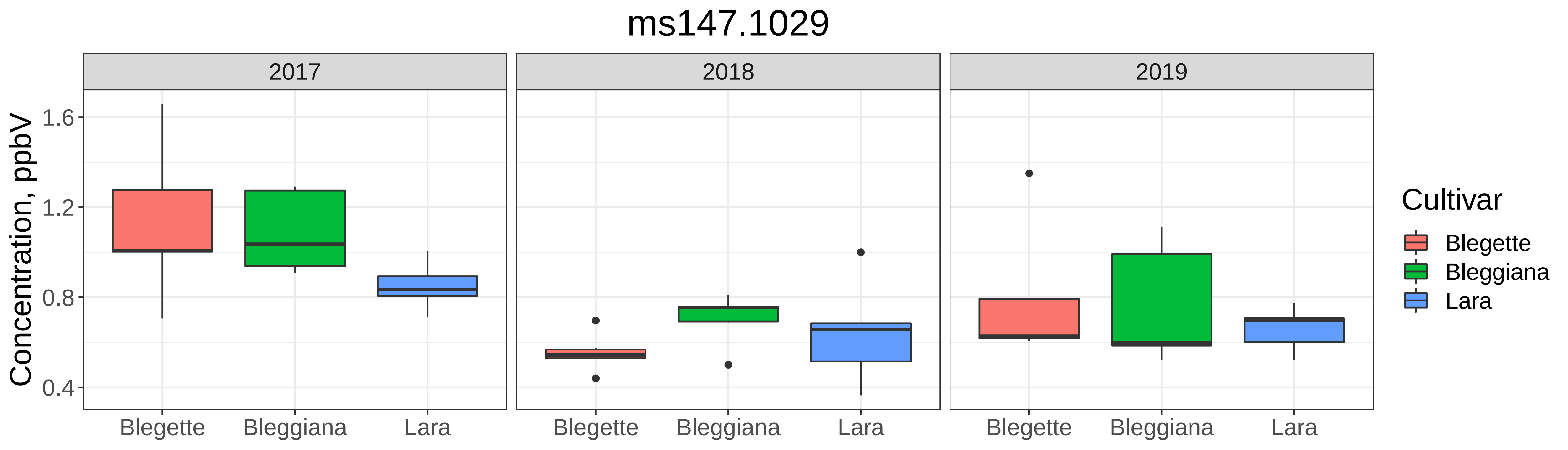

ms147.1029
2017
2018
2019
Concentration, ppbV
1.6
1.2
0.8
0.4
Blegette
Bleggiana
Lara
Cultivar
Blegette
Bleggiana
Lara
